# A burden of proof study of the effects of exposure to high fasting plasma glucose on the risk of seven types of cancer

**DOI:** 10.1038/s41598-025-13045-9

**Published:** 2025-08-07

**Authors:** Paula Portal Teixeira, Yvonne Yiru Xu, Aleksandr Aravkin, Peng Zheng, Lisa M. Force, Jonathan Kocarnik, Susan McLaughlin, Theo Vos, Hailey Lenox, Simon I. Hay, Bruce Bartholow Duncan, Verônica Colpani, Chris Murray, Fernando Gerchman, Kanyin Liane Ong

**Affiliations:** 1 Institute for Health Metrics and Evaluation, University of Washington, Seattle, WA USA; 2 Universidade Federal do Rio Grande do Sul, Porto Alegre, RS 90035-903 Brazil; 3 Centre of Health Technology Assessment, Hospital Sírio-Libanês, São Paulo, SP Brazil

**Keywords:** Cancer, Endocrinology, Risk factors

## Abstract

Cancer is a major public health problem strongly influenced by genetic factors and aging; however, a proportion of its burden can be attributed to potentially modifiable risk factors. We undertook a systematic review and meta-analysis of the existing evidence to quantify and assess the relationship between high fasting plasma glucose (FPG) levels and the risk of developing and dying for seven types of cancer (selected for having attributable burden from FPG in GBD). Using Burden of Proof methods that provide a conservative interpretation of the evidence, we found moderate relationships between high FPG and the risk of breast, pancreatic, and colorectal cancer. Weak relationships were observed with bladder, liver, ovarian, and tracheal, bronchus and lung cancers. These findings should galvanize the global community’s efforts in addressing the increasing burden of high blood sugar and inform the potential impact of different hypoglycemic treatments on reducing the burden of cancer.

**Supplementary Information:**

The online version contains supplementary material available at 10.1038/s41598-025-13045-9.

## Introduction

Cancer is a major public health problem and one of the leading causes of death globally, responsible for 9.83 million (95% uncertainty interval (UI) 9.07–10.52) deaths and 252.09 million (236.33–269.34) disability-adjusted life years (DALYs) in 2021^1,2^. Between 2010 and 2019, the number of new cancer cases and deaths globally increased by 26.3% and 20.9%^3^, respectively, and, based on the GLOBOCAN estimates produced by the International Agency for Research on Cancer, a 47% increase in new cases is projected by 2040 if the rise is not checked^4^. According to the Global Burden of Diseases, Injuries, and Risk Factors Study (GBD) from 2021, the five leading causes of cancer-related DALYs were tracheal, bronchus and lung cancer, colorectal cancer (CRC), stomach cancer, breast cancer, and esophageal cancer^1,2^. Although cancer risk is strongly influenced by genetic factors and aging, a significant proportion of cancer burden can be attributed to potentially modifiable risk factors, such as smoking, excess body weight, and dietary factors^5^.

Based on recent GBD estimates from 2021, high levels of fasting plasma glucose (FPG) had an increasing global impact on health. High FPG and high body mass index (BMI) were the only two risk factors that increased in age-standardized attributable DALYs rates between 2000 and 2021, which contrasted with decreases over the same period for all the other leading 25 GBD Level 3 risk factors^6^. High FPG has been shown to be associated with an increased risk of cancer and contribute to its progression and mortality^7,8^with the estimated magnitude of association varying by cancer type^9^. Studies have suggested that liver and pancreatic cancers have a higher incidence and higher risk of mortality in people with diabetes mellitus (DM)^8,10^. In 2021, high FPG was one of the top five leading risk factors for cancer, contributing 2.8% (0.3–5.4) of risk-attributable cancer DALYs^1,2^. However, while most studies assessing the potential effect of glucose levels on cancer burden have highlighted DM- or prediabetes-associated cancer incidence and mortality^11^there are inconsistent findings related to whether there is a dose-response relationship between increasing levels of FPG and the risk of different cancer types^12^.

In this study, which is part of GBD 2021, we conducted a systematic review and meta-analysis using Burden of Proof (BoP) methods^13^ to quantify and describe the relationship between FPG levels along the blood glucose continuum and the risk of seven types of cancer: bladder cancer; liver cancer; tracheal, bronchus and lung cancer; breast cancer; colon and rectum (colorectal) cancer; pancreatic cancer; and ovarian cancer. The BoP methodology generates a mean relative-risk (RR) function using meta-regression techniques designed to capture the potentially non-linear shape of the risk-outcome relationship, trims data outliers, tests and adjusts for known heterogeneity across input study characteristics, and quantifies and incorporates the remaining unexplained between-study heterogeneity into uncertainty estimates to compute a burden of proof risk function (BPRF). The BPRF represents a highly conservative estimate of the excess risk (here, one of seven different types of cancer) associated with exposure to a specified risk factor (here, elevated FPG levels). This helps to understand which health recommendations may have the biggest impact, allowing both individuals and policymakers to understand the strength of evidence behind a risk factor and a health outcome. For ease of interpretation and comparability across risk-outcome pairs, we converted the BPRF into a risk-outcome score (ROS) and mapped it onto a star-rating scale, yielding a single metric reflecting both the magnitude of association and strength of underlying evidence between elevated FPG levels and each type of cancer.

## Results

### Overview

**Fig. 1:**
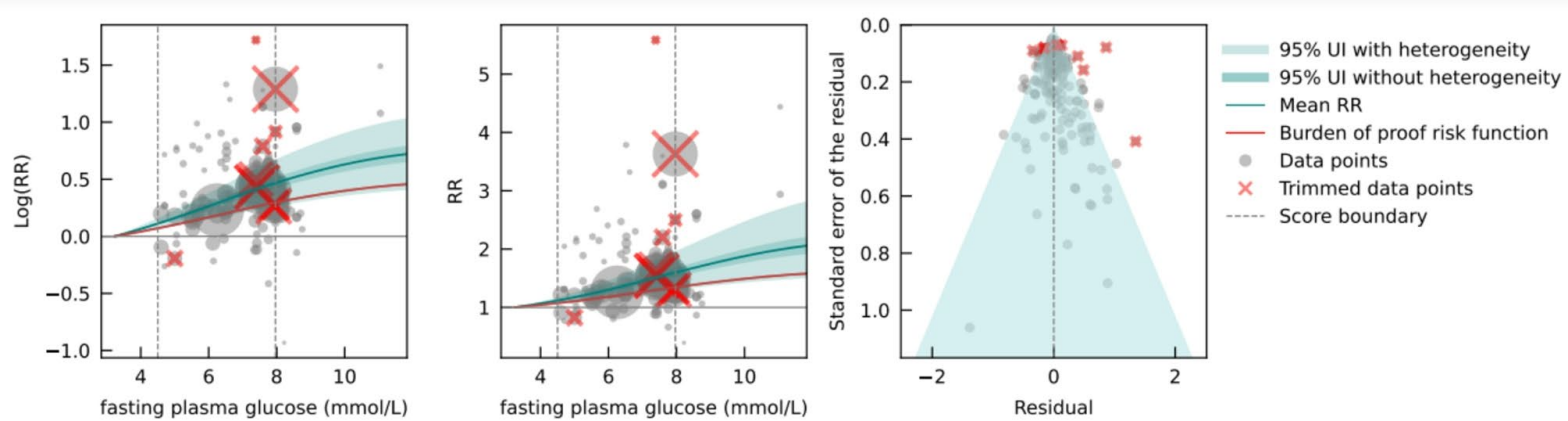
Fasting plasma glucose levels and colorectal cancer log relative risk (**a**), relative risk (**b**), and residuals by estimated standard deviation (**c**).

**Fig. 2:**
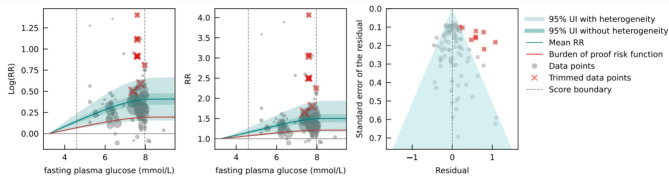
Fasting plasma glucose levels and breast cancer log relative risk (**a**), relative risk (**b**), and residuals by estimated standard deviation (**c**).

**Fig. 3:**
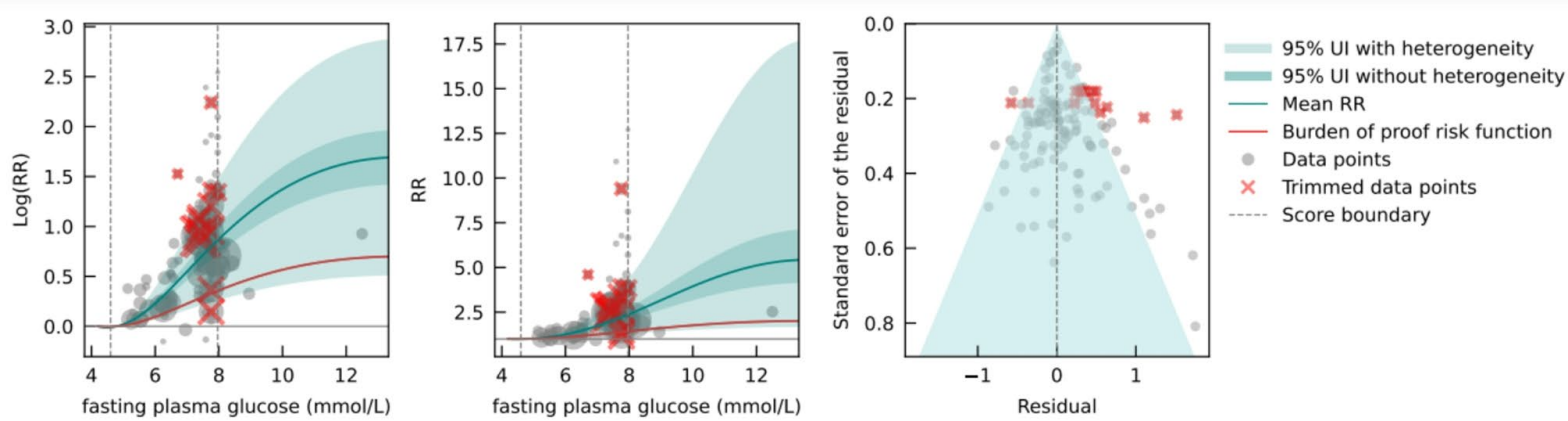
Fasting plasma glucose levels and pancreas cancer log relative risk (**a**), relative risk (**b**), and residuals by estimated standard deviation (**c**).

We conducted a systematic review of published studies on the relationship between FPG and seven selected types of cancer, according to Preferred Reporting Items for Systematic Reviews and Meta-Analyses (PRISMA) guidelines^14^. A PRISMA diagram delineating our updated systematic review is available in Supplementary Fig. 1, as well as a detailed description of reasons for exclusion at each phase of the selection process at Supplementary Material, Sect. 2. In previous iterations of the GBD we identified 113 publications between 1970 and 2019 that fulfilled our inclusion criteria. We then updated the systematic review (September 2023), yielding 4,098 relevant records published after 2020, of which 69 studies were selected after title and abstract screening and full text analysis. A total of 147 studies were then included, 58 with data on colorectal cancer^12,15–71^49 on breast cancer^12,15,17,25,29–35,37–41,43,44,46,51,54–60,62,68,72–91^, 50 on liver cancer^12,15,25,29–32,34–42,44,46,51,54,56–60,62,65,68,86,92–112^, 55 on pancreatic cancer^12,15,29–32,34–42,46,51,55,56,58,59,62,68,86,98,108,113–141^, 32 on tracheal, bronchus, and lung cancer^12,15,25,30,31,33–36,38–41,43,44,46,51,55–58,60,62,68,86,88,142–147^, 34 on bladder cancer^15,29–31,34–42,46,54–56,58–60,62,68,88,148–158^and 22 on ovarian cancer^29,30,32,34,37,54–56,58–60,62,68,86,88,112,159–164^. In our analyses for each of these seven types of cancer, we extracted and combined data reporting both cancer incidence and mortality. In each analysis, we included a mortality-specific covariate but did not create separate incidence and mortality models. Details on each study such as author, year of publication, study name and design, location, age range, endpoint, disease ascertainment, sample size and number of events can be found in Sect. 3 of Supplementary Material. A summary of numerical results, BPRF values, ROS values, star ratings, and covariates adjusted in the analysis for each cancer type are available in Table 1. Figures 1 and 2, and 3 present FPG-cancer relative risk functions for those risk-outcome pairs for which the association received a rating of three stars or higher, and the remaining risk curves can be accessed in the Supplementary Material (Figures S2-S5).

**Table 1:** Strength of the evidence for the relationship between exposure to high FPG levels and the seven health outcomes analyzed.

Health outcome	85th percentile of FPG (mmol/L)	RR (95% UI) without γ	RR (95% UI) with γ	Exposure-averaged BPRF	Average risk increase (%)	ROS	Star rating	Pub. bias	No. of studies	Selected bias covariates
Colon and rectum cancer	7.96	1.6 (1.53–1.67)	1.6 (1.3–1.96)	1.20	20%	0.18	***	No	58	Incident DM; Different DM definitions; Mortality-specific analysis; Study outcome consistent with ICD-10 code for colorectal cancer
Breast cancer	7.96	1.5 (1.4–1.61)	1.49 (1.16–1.92)	1.15	15%	0.14	***	No	49	Different DM definitions; Mortality-specific analysis; Geographically representative sample
Pancreatic cancer	7.96	2.36 (2.08–2.72)	2.37 (1.3–4.32)	1.16	16%	0.14	***	Yes	55	FPG levels imputed; Mortality specific analysis; Sex-specific analysis; Geographically representative sample; Study outcome consistent with ICD-10 code for pancreatic cancer
Liver cancer	7.96	2.31 (2.09–2.59)	2.31 (0.99–5.41)	1.05	5%	0.05	**	No	50	Different DM definition; Sex-specific analysis; Geographically representative sample
Tracheal, bronchus, and lung cancer	7.78	1.25 (1.14–1.38)	1.25 (1.02–1.52)	1.04	4%	0.04	**	Yes	32	Incident DM
Bladder cancer	7.96	1.55 (1.34–1.81)	1.55 (0.96–2.52)	1.02	2%	0.02	**	No	34	FPG levels imputation; Different DM definition; Study outcome consistent with ICD-10 code for bladder cancer
Ovarian cancer	7.95	1.09 (1.02–1.17)	1.09 (0.87–1.38)	0.94	N/A	-0.06	*	No	22	Study outcome consistent with ICD-10 code for ovarian cancer

The reported relative risk (RR) and its 95% uncertainty interval (UI) reflect the risk an individual who has been exposed to FPG has of developing the outcome of interest relative to that of someone who has not been exposed to FPG. Gamma (γ) is the estimated between-study heterogeneity. We report the 95% UI when not incorporating between-study heterogeneity (γ) − “95% UI without γ” − and when accounting for between-study heterogeneity − “95% UI with γ.” The Burden of Proof Risk Function (BPRF) is calculated for risk-outcome pairs that were found to have significant relationships at an 0.05 level of significance when not incorporating between-study heterogeneity (i.e., the lower bound of the 95% UI without γ does not cross the null RR value of one). The BPRF corresponds to the 5th quantile estimate of relative risk, when additionally accounting for between-study heterogeneity closest to the null for each risk–outcome pair, and it reflects the most conservative estimate of excess risk associated with FPG that is consistent with the available data. Negative ROSs indicate that the association’s evidence is weak and inconsistent. For ease of interpretation, we have transformed the ROS and BPRF into a star rating (0–5) with a higher rating representing a larger effect with stronger evidence. The potential existence of publication bias, which, if present, would affect the validity of the results, was tested using Egger’s Regression. Included studies represent all available relevant data identified through our systematic reviews from January 1970 through May 2023. The selected bias covariates were chosen for inclusion in the model using an algorithm that systematically detects bias covariates that correspond to significant sources of bias in the observations included. If selected, the observations were adjusted to better reflect the gold standard values of the covariate. See the Supplementary Material for more information about the definition and how each bias covariates that were extracted for in each model.

For harmful risk factors, the BPRF is defined as the 5th percentile RR curve – inclusive of the uncertainty derived from unexplained between-study heterogeneity – closest to the null (the function representing a relationship in which change in risk exposure has no effect on the specified health outcome). The ROS is the signed value of the log BPRF, averaged across the 15th to 85th percentiles of FPG exposure (as observed in the input data). A higher ROS corresponds to a stronger risk-outcome association and/or more consistent underlying evidence. Star ratings range from one, reflecting a weak association and/or inconsistent evidence, to five, reflecting a strong association and/or consistent evidence (Table 2).

**Table 2:** Star rating categories for risk-outcome scores.

	Percentage of increase in risk of outcome in those exposed to harmful risk factors	Risk-outcome score (ROS) range	Health outcomes
One star	0%	< 0.00	Ovarian cancer
Two stars	0–15%	0.00 to 0.14	Bladder cancer, tracheal, bronchus and lung cancer, and liver cancer
Three stars	> 15–50%	> 0.15 to 0.41	Pancreas cancer, breast cancer and colorectal cancer
Four stars	> 50–85%	> 0.41 to 0.62	None
Five stars	> 85%	> 0.62	None

### Colorectal cancer

We identified 58 studies assessing the relationship between FPG levels and the risk of colorectal cancer with sample sizes ranging from 135 to 25,709,497 participants. The studies identified 283,222 incident cases and 85,054 deaths. Most studies were prospective cohorts (*n* = 34), followed by those with a case-control design (*n* = 12), retrospective cohorts (*n* = 9), and case-cohorts (*n* = 3). Twenty studies were conducted in Europe, 20 in Asia, 15 in North America, and three in Australasia. In most studies (*n* = 34), FPG exposure was identified by the presence/absence of DM based on administrative records, treatment use, or self-reported physician diagnosis, while 20 studies defined FPG cutoffs for DM diagnosis or presented FPG exposure by categorical levels. The remaining four studies defined blood glucose based on HbA1C.

The analysis was adjusted for the different definitions of DM in the included studies, for the presence of studies that considered only incident cases of DM as the exposure, for whether the study outcome was consistent with the definition of colorectal cancer according to the International Classification of Diseases [ICD] – 10: C18.0 – C21.9, and for studies with mortality-specific analyses (all listed in Table 2). The mean risk curve showed that, at an FPG level of 7.96 mmol/L (equivalent to the 85th percentile of exposure observed in the data) in comparison to a reference level of 3.23 mmol/L, the relative risk (RR) for colorectal cancer was 1.60 (1.30–1.96, all 95% UIs reported are inclusive of between-study heterogeneity)​. The exposure-averaged BPRF measure was equal to 1.20, which indicates that – when incorporating unexplained between-study heterogeneity that remains after accounting for known variation in study design characteristics – there was at least a 20% average increase in colorectal cancer risk at FPG levels across the 15th to 85th percentiles (4.50 to 7.96 mmol/L) of the exposure range (Fig. 1). This corresponds to a ROS of 0.18 and a three-star rating, indicating that the association between high FPG and colorectal cancer risk is characterized by moderate effect size and/or evidence strength, based on the available evidence. No evidence of publication bias was identified by Egger’s regression test or from visual inspection of the funnel plot (Fig. 1c). Sensitivity analysis, including only cohort studies, showed similar results in terms of ROS and star rating, showing robustness of the findings (Supplementary Table S19).

We also estimated the mean risk of colorectal cancer at two different clinically meaningful FPG levels: (1) 6.1 mmol/L, which represents the cutoff for defining “prediabetes” according to the American Diabetes Association (ADA) Statements^165^; and (2) 7.0 mmol/L, the cutoff for defining DM according to the ADA. At FPG levels of prediabetes, we observed a mean RR of colorectal cancer of 1.32 (1.17–1.48), which increased to 1.45 (1.23–1.71 UIs inclusive of between-study heterogeneity) at FPG levels consistent with DM (Supplementary Table S10).

### Breast cancer

We identified 49 studies assessing the relationship between FPG levels and the risk of breast cancer in sample sizes ranging from 64 to 25,709,497 participants. Overall, 113,264 new cases and 32,946 deaths from breast cancer were reported. The majority of studies were prospective cohorts (*n* = 29), followed by those with a case-control design (*n* = 13), retrospective cohorts (*n* = 6), and one case-cohort (*n* = 1). Most of the studies were conducted in the Americas (*n* = 17), 14 in Europe, 16 were from Asia, and the remaining two from New Zealand. In 67.3% (*n* = 33) of the studies, FPG exposure was identified the presence/absence of DM based on administrative records, treatment use, or self-reported physician diagnosis, while 13 studies defined FPG cutoffs for DM diagnosis or presented the exposure as FPG categories, and the remaining four studies presented data on HbA1C.

The analysis – adjusted for significant bias covariates (different DM definitions across studies, mortality-specific analysis, and whether the study sample was geographically representative) – showed that, in comparison to the reference FPG level of 3.23 mmol/L, the mean RR for breast cancer at 7.96 mmol/L (the 85th percentile of FPG exposure) was 1.49 (1.16–1.92)​. The BPRF was 1.15, indicating that FPG levels across the 15th to 85th percentiles (4.59 to 7.96 mmol/L) were significantly associated with at least a 15% average increase in risk of breast cancer (Fig. 2). This corresponds, on a log scale, to a ROS of 0.14 and a three-star rating, also indicating a moderate effect size and/or certainty of evidence for the association between high FPG and the risk of breast cancer. No evidence of publication bias was identified by Egger’s regression test and by the funnel plot. Sensitivity analysis including only cohort studies (*n* = 36) resulted in a ROS of 0.13, which reduced the star rating to two stars, corresponding to a weak effect size and/or certainty of evidence. Notably, however, the estimated ROS is very close to the bound of 0.1398 that differentiates two from three-star ratings (Supplementary Table S9).

In the analysis assessing the risk of breast cancer at prediabetes and DM thresholds, at FPG levels of 6.1 mmol/L, which corresponds to a prediabetic state, we observed a significant mean RR of breast cancer of 1.34 (1.12–1.60), which increased to 1.44 (1.15–1.80) at FPG levels consistent with DM (Supplementary Table S10).

### Pancreatic cancer

We identified 55 studies assessing the relationship between FPG levels and the risk of pancreatic cancer in sample sizes ranging from 188 to 25,709,497 participants. Overall, approximately 710,277 new cases of pancreatic cancer and 55,443 deaths from pancreatic cancer occurred. The majority of studies were prospective cohorts (*n* = 31), followed by those with a case-control design (*n* = 13), retrospective cohorts (*n* = 7), nested case-control (*n* = 3), and case-cohort (*n* = 1). Twenty studies were conducted in Europe, 21 in Asia, 11 in North America and the remaining three in Oceania, two in New Zealand, and one in Australia. In most studies (*n* = 38), FPG exposure was identified by the presence/absence of DM based on administrative records, treatment use, or self-reported physician diagnosis, while 16 studies defined FPG cutoffs for DM diagnosis or presented FPG exposure levels, and one study reported data on HbA1C levels.

Compared to a reference FPG level of 4.20 mmol/L, the mean RR for pancreatic cancer at 7.96 mmol/L (the 85th percentile of FPG exposure) was 2.37 (1.30–4.32).​ The BPRF was 1.16, suggesting that FPG levels across the 15th to 85th percentiles (4.59 to 7.96 mmol/L) were significantly associated with at least 16% higher risk of pancreatic cancer (Fig. 3), corresponding to a ROS of 0.14 and a three-star rating. Based on our analysis, we therefore found a moderate effect size and/or certainty of evidence for the association between high FPG levels and the risk of pancreatic cancer. This analysis was adjusted for the need for FPG imputation, the presence of mortality and sex-specific analyses, whether the study sample was geographically representative, and whether the study outcome was consistent with the definition of pancreatic cancer according to the International Classification of Diseases [ICD] – 10: C25.0 – C25.9. Egger’s regression test and the funnel plot indicated the presence of publication bias (*p* = 0.04). Sensitivity analysis including only studies with cohort design (Supplementary Table S9) yielded similar results to the full analysis, with no changes in the interpretation regarding the effect size and strength of evidence.

When evaluating the risk of pancreas cancer at prediabetes and DM thresholds, at FPG levels of 6.1 mmol/L (prediabetes), we observed a significant mean RR of pancreatic cancer of 1.30 (1.08–1.55), which increased to 1.73 (1.18–2.54) at FPG levels consistent with DM (Supplementary Table S10).

### Liver cancer

We identified 50 studies assessing the relationship between FPG levels and the risk of liver cancer in sample sizes ranging from 30 to 25,709,497 participants. Overall, approximately 94,378 new cases of liver cancer and 84,513 cases of deaths due to liver cancer occurred. The majority of studies were prospective cohorts (*n* = 34), followed by those with a case-control design (*n* = 7), retrospective cohorts (*n* = 6), nested case-control (*n* = 2), and case-cohort (*n* = 1). 23 studies were conducted in Asia, 15 in Europe, 10 in the Americas, and the remaining two in New Zealand. In most studies (*n* = 30), FPG exposure was characterized by the presence/absence of DM based on administrative records, treatment use, or self-reported physician diagnosis. 17 studies defined FPG cutoffs for DM diagnosis or presented the exposure as FPG categories, and the remaining three reported data on HbA1C levels.

After adjusting for the different definitions of DM in the included studies, for the presence of sex-specific analysis, and for whether the study sample was geographically representative, the mean RR for liver cancer was 2.31 (0.99–5.31) at an FPG level of 7.96 mmol/L (the 85th percentile of exposure) compared to a reference level of 2.50 mmol/L​. However, the exposure-averaged BPRF was equal to 1.05, indicating that FPG levels across 4.59 to 7.96 mmol/L (15th to 85th percentiles of exposure) were associated with an average of at least 5% higher risk of liver cancer (Figure S2). This corresponds to a ROS of 0.05 and a two-star rating, indicating a weak effect size and/or certainty of evidence for the association. No evidence of publication bias was identified, based on Egger’s regression and visual inspection of the funnel plot (Figure S2c). Sensitivity analysis including only cohort studies yielded similar results to the full analysis in terms of mean RR and star rating but reduced the ROS to 0.04 (Supplementary Table S9).

When assessing the risk of liver cancer at prediabetes and DM thresholds, at FPG levels corresponding to prediabetes (6.1 mmol/L), we observed a nonsignificant mean RR of liver cancer of 1.28 (1.00–1.63), which increased to 1.65 (0.99–2.75) at FPG levels consistent with DM, although the association remained non-significant (Supplementary Table S10).

### Tracheal, bronchus and lung cancer

We identified 32 studies assessing the relationship between FPG levels and the risk of lung cancer in sample sizes ranging from 959 to 25,709,497 participants. Overall, approximately 191,107 new cases of lung cancer and 179,954 deaths due to lung cancer occurred. The majority of studies were prospective cohorts (*n* = 22), followed by retrospective cohorts (*n* = 6), case-control (*n* = 3), and one case-cohort. Thirteen studies were conducted in Europe, nine in Asia, seven in North America, and the remaining three in New Zealand. In most of the studies (*n* = 22), FPG exposure was identified by the presence/absence of DM based on administrative records, treatment use, or self-reported physician diagnosis, while seven studies defined FPG cutoffs for DM diagnosis or reported the exposure as FPG categories, two studies presented data on HbA1C levels, and one reported glucose levels after a 2 h-oral glucose tolerance test.

At an FPG level of 7.78 mmol/L (the 85th percentile of exposure), analysis adjusted for the presence of studies that considered only incident cases of DM as the exposure showed a mean RR for lung cancer of 1.25 (1.02–1.52) compared to a reference FPG level of 2.50 mmol/L​. The exposure-averaged BPRF was 1.04, indicating that FPG levels across 4.59 to 7.78 mmol/L (15th to 85th percentiles of exposure) were associated with an average increase of at least 4% in the risk of lung cancer (Figure S3), corresponding to a ROS of 0.04 and a two-star rating. The effect size and strength of evidence was therefore classified as weak. Publication bias was identified by Egger’s regression test (*p* = 0.02) and by the visual inspection of the funnel plot. When sensitivity analysis including only cohort studies was performed, the mean RR was 1.31 (0.87–1.97, inclusive of between-study heterogeneity), resulting in an ROS of -0.06, indicating weak evidence of association due to a one-star rating (Supplementary Table S9).

At FPG levels corresponding to prediabetes (6.1 mmol/L), the mean RR of lung cancer was 1.17 (1.02–1.35), which increased to1.23 (1.02–1.47) at FPG levels consistent with DM (Supplementary Table S10).

### Bladder cancer

We identified 34 studies assessing the relationship between FPG levels and the risk of bladder cancer in sample sizes ranging from 51 to 25,709,497 participants. Overall, approximately 71,430 new cases of bladder cancer and 27,891 deaths due to bladder cancer occurred. The majority of studies were prospective cohorts (*n* = 21), followed by those with a case-control design (*n* = 8), retrospective cohorts (*n* = 3), and case-cohorts (*n* = 2). Fourteen studies were conducted in Europe, 10 in Asia, eight in North America, and the remaining two in New Zealand. In most of studies (*n* = 27), FPG exposure was identified by the presence/absence of DM based on administrative records, treatment use, or self-reported physician diagnosis, while five studies defined FPG cutoffs for DM diagnosis or presented the exposure as FPG levels, and the remaining two studies reported levels of HbA1C.

The analysis – adjusted for the different DM definitions across studies, whether the study outcome was consistent with the definition of bladder cancer according to the International Classification of Diseases [ICD] – 10: C67.0 – C67.9, and the need for FPG imputation – resulted in a mean RR of 1.25 (1.02–1.52) for bladder cancer at a FPG level of 7.96 mmol/L (85th exposure percentile), in comparison to a reference level of 4.20 mmol/L. The exposure-averaged BPRF was equal to 1.02 and indicated that FPG levels across 5.53 to 7.96 mmol/L (15th to 85th percentiles of exposure) were associated with an average increase of at least 2% in the risk of bladder cancer (Figure S4), corresponding to a ROS of 0.02 and a two-star rating. This suggests that the magnitude and/or evidence of an association are weak, given the current evidence. We did not identify evidence of publication bias by Egger’s regression test and visual inspection of the funnel plot. As with lung cancer, sensitivity analysis including cohort studies yielded an RR of 1.31 (0.87–1.97) and an ROS of -0.08, which downgraded the star rating to one star (Supplementary Table S10).

When examining the risk of bladder cancer at prediabetes and DM thresholds, at FPG levels of 6.1 mmol/L, the estimated mean RR of bladder cancer was 1.29 (0.97–1.70). At FPG levels of 7.0 mmol/L, which corresponds to DM diagnosis, the RR was 1.44 (0.96–2.15), both showing non-significant relationships (Supplementary Table S10).

### Ovarian cancer

We identified 22 studies assessing the relationship between FPG levels and the risk of ovarian cancer in sample sizes ranging from 330 to 8,309,393 participants. Overall, approximately 469,056 new cases of ovarian cancer and 13,424 deaths due to ovarian cancer occurred. The majority of studies were prospective cohorts (*n* = 16), two were retrospective cohorts, three were case-control studies, and the remaining was a case-cohort study. The majority (*n* = 10) were conducted in Europe, five in Asia, five in North America, and the remaining two in New Zealand. In most of the studies (*n* = 13), the exposure was characterized as the presence/absence of DM based on administrative records, treatment use, or self-reported physician diagnosis, while six studies defined FPG cutoffs for DM diagnosis or presented FPG exposure levels, and three studies showed exposure data as HbA1C levels.

The model was adjusted for whether the study outcome was consistent with the definition of ovarian cancer according to the International Classification of Diseases [ICD] – 10: C56.0 – C56.9. At an FPG level of 7.95 mmol/L (85th percentile of exposure) relative to a reference level of 3.98 mmol/L, the analysis yielded a mean RR of 1.09 (0.87–1.38) for ovarian cancer (Figure S5)​. The ROS was − 0.06, yielding a one-star rating. There was no evidence of publication bias, as assessed by Egger’s regression test and visual inspection of the funnel plot. In sensitivity analysis including only cohort studies investigating the FPG-ovarian cancer relationship, the ROS rose in comparison to the full analysis to 0.01 (based on a BPRF of 1.01), increasing the star rating to two stars (Supplementary Table S9).

In the analysis assessing the risk of ovarian cancer at prediabetes and DM thresholds, at FPG levels of 6.1 mmol/L, which corresponds to a prediabetic state, we observed a non-significant mean RR of ovarian cancer of 1.04 (0.94–1.15), which was almost the same for DM thresholds, with 1.07 (0.90–1.28) at FPG levels consistent with DM (Supplementary Table S10).

## Discussion

In this study, we used the BoP framework to assess the effect size and strength of evidence for the association between FPG levels and the risk of seven types of cancer. Even with a particularly conservative interpretation of the evidence, which considered between-study heterogeneity remaining after accounting for known variation in input study characteristics, we showed positive and moderate relationships between elevated levels of FPG and the risk of breast, pancreas, and colorectal cancers, with exposure-averaged increases of at least 15%, 16% and 20%, respectively, over the 15th to 85th percentiles of observed FPG exposure. Weak relationships were observed with bladder, tracheal, bronchus and lung, and liver cancers based on positive ROSs that pointed to average risk increases of at least 2%, 4%, and 5%, respectively. Our approach yielded a negative ROS for high FPG and ovarian cancer, indicating a weak relationship characterized by a small effect size and/or inconsistent evidence, based on the data available.

This is, to our knowledge, the first systematic review reporting the risk of seven different types of cancer along the entire continuum of FPG levels, based on evidence from 147 different studies. Recently published meta-analyses have compared different levels of exposure to FPG and showed higher risks of pancreatic cancer^166^liver cancer^167^colorectal cancer^168^ and ovarian cancer^9,169^ when compared to our results derived from the BoP methodology, which is consistent with the BPRF and associated ROS and star rating representing the lowest estimate of excess health risk for a harmful risk factor based on the available data. When we estimated the risk of the seven types of cancer at clinically relevant FPG levels, corresponding to prediabetic and diabetic states according to the ADA Diagnostic Statements, significantly increased risk of breast, colorectal, and pancreatic cancer were observed at both prediabetes and DM thresholds, which is in accordance with previously published reviews^170^. For the other types of cancer (liver, lung, bladder, and ovarian cancer), we observed no significant association at both FPG cutoffs, which contradicts previously observed increases in the risk of bladder and lung cancer^9^.

The rise in DM is expected to continue with forecasts of DM prevalence exceeding 1 billion people by 2050^171^. Although reductions in FPG levels would be expected to decrease cancer risk by limiting the incidence of prediabetes and DM, the effect of treating hyperglycemia on cancer prevention is still unproven^172,173^ and should therefore be tested by further long-term studies. Behavioral risk factors that contribute to elevated FPG levels could also be a point of action, including efforts to promote healthier dietary patterns and smoking cessation, reduce alcohol consumption, boost physical activity levels, and control weight^9,174^. There is a need for future studies to investigate the effectiveness of population health approaches to tax nutrient-poor foods, increase access to nutrient-rich foods, and improve access to exercise opportunities on both reducing FPG levels and reducing cancer risk^174,175^; although numerous interventions to reduce glucose levels have been proposed, progress has been slow and long-term reductions of FPG at a population level have been limited.

Our study has several strengths. First, we were able to estimate the dose-response relationship between the entire continuum of FPG levels and the risk of seven different types of cancer by using imputation methods based on regional distribution of FPG values among individual-level microdata, even when studies included in this analysis reported results as categorical exposures (such as DM vs. no DM, or different categories of FPG). Second, our analytical framework allowed us to systematically adjust for potential bias covariates, allowing for the inclusion of many studies by accounting for heterogenous study designs, measures of effect, and study characteristics. Third, the BPRF (and associated ROS and star ratings) provides conservative estimates of excess risk associated with exposure to a risk factor by incorporating the unexplained between-study heterogeneity that remains after accounting for known heterogeneity in study characteristics, yielding a measure that reflects both the magnitude of the association and the consistency of the underlying evidence^13,176^. Finally, converting the BPRF into a star rating is a useful tool for policymakers and practitioners to make decisions about exposure to specific risk factors and to compare the associations that characterize different risk-outcome pairs, increasing the transparency around scientific knowledge about human health risks. It can be used as a standardised method to prioritise research, mainly focusing on one and two-star risk-outcome pairs.

In addition to the strengths mentioned above, this analysis also has limitations that should be considered when interpreting our results. We performed the literature search solely on PubMed, which can result in an unrepresentative sample of the published literature^177^. Besides that, we were able to capture large and well-designed population-based studies and only identified six new studies (4% of the total 147 studies included in our analysis) after assessing all the underlying studies in the meta-analyses that fulfilled our inclusion criteria. Second, we identified potential publication or reporting bias for pancreas and tracheal, bronchus and lung cancers, suggesting a propensity for studies with positive associations within the published literature. In our models, 10% of input data was trimmed to eliminate extreme values and ensure that is robust to spurious observations, but following the general literature, no corrections for publication bias were incorporated to the models. Third, besides using a standardised methodology to statistically account for differences and limitations between studies, the method can be limited by the underlying data reported in published studies, which are used as input data to our relative risk curves. For example, the FPG exposure definition and unit of exposure varied across studies, and almost 65% of the data were based on studies comparing cancer risk in the presence or absence of DM; even studies reporting FPG levels presented their results stratified by FPG categories. In addition, as almost all studies reported exposure data only at one time point, our results may not capture cancer risk in the case of declining blood glucose levels due to the use of pharmacological intervention or increasing blood glucose levels over time. The time component is especially important from a public health standpoint when thinking about when and where to intervene in cancer prevention. Finally, this study only assessed cancers for which the relationship with FPG has already been quantified in previous iterations of GBD, and during the systematic review we identified studies for additional cancers — stomach, kidney, prostate, and esophageal cancer — that are also part of the GBD cause list. This will be a future area of work.

In this study, we found that the association between FPG and cancer varied by type, with our conservative interpretation of the evidence showing a moderate association between FPG exposure and the risk of pancreatic, breast, and colorectal cancer, and a weak but positive association with risk of lung, bladder, liver, and ovarian cancers. These findings should galvanize the global community’s focus and efforts to address the increasing health burden of high blood sugar and to prevent cancer’s development and death. The negative ROS and one-star rating for ovarian cancer may be due to a lack of high-quality data, pointing to the necessity for more quality well-designed prospective cohort studies to evaluate the FPG-ovarian cancer relationship, considering that there is a potential for future changes to the existing findings. Public health and clinical evidence-based interventions focused on preventing and managing DM by reducing FPG levels may be useful in reducing the burden of cancer related to metabolic disease.

## Methods

### Overview

The BPRF methodology is a Bayesian meta-analytic approach to categorize the strength of the evidence for each risk-outcome pair, accounting for many potential biases such as those related to study design and publication bias, detecting outliers using robust statistical trimming methods. This method summarizes this information to calculate risk–outcome scores (ROSs), which are categorized into 5 levels, as a star rating, allowing easier interpretation of the results.

The methodology comprises 6 main steps that have been previously described^13^: (1) perform a systematic review to gather published evidence, extract the data and prepare for analysis; (2) estimate the shape of the relationship of exposure versus relative risk (RR), integrating over ranges of alternative and reference exposures and allowing different reference groups to be considered; (3) test and adjust for systematic biases as a function of study attributes through bias covariates; (4) quantify remaining between-study heterogeneity while adjusting for within-study correlation, as well as the number of studies; (5) assess evidence for small-study effects to evaluate potential risk of publication or reporting bias; and (6) estimate the BPRF, quantifying a interpretation of the average risk increase across the range of exposure supported by the evidence to compute ROS and classify it into five star categories.

The ultimate goal is to estimate the smallest level of excess risk obtained for FPG that is consistent with the data for each cancer type. The star rating of an FPG–cancer pair reflects the average magnitude of the association, where one star indicates that there is no relationship between FPG and the corresponding type of cancer, and five stars indicates a large effect and strong evidence of association. In this classification, ROS lower than 0.000 are rated as one star, scores from 0.000 to 0.1398 as two stars, from 0.1398 to 0.4055 as three stars, from 0.4055 to 0.6152 as four stars and ROS higher than 0.6152 are rated as five stars, as described in Table 1.

### Systematic review

The protocol of this updated systematic review was registered in the International Prospective Registry of Systematic Reviews (PROSPERO, protocol number CRD42023464975) and follows the Preferred Reporting Items for Systematic Reviews and Meta-Analyses (PRISMA) reporting guidelines and the Guidelines on Accurate and Transparent Health Estimate Reporting (GATHER) recommendations. The original search was performed on PubMed as part of the GBD study 2019, including studies from 1970 to 2019, which was then updated to identify articles published from 01/01/2020 to 09/05/2023. There were only two studies published before 1970, neither of which met the inclusion criteria. No language restrictions were applied. The full search string and all eligibility criteria are detailed in Sect. 2 of Supplementary Material.

Studies were included if they had a longitudinal design, including prospective or retrospective cohorts, case-control or nested case-control. Meta-analyses that fulfilled the selection criteria in title and abstract screening were also reviewed in full text to identify primary studies not captured in our literature search. Only studies that reported a continuous measure of glycemic control, such as FPG, glycated hemoglobin (HbA1C) or a glycemic measure during an oral glucose tolerance test (OGTT), or the prevalence of DM diagnosed by a physician or reported by administrative records were included. We accepted results presented as odds ratio (OR), relative risk (RR), hazards ratio (HR), incidence rate ratio (IRR), standardized mortality ratio (SMR), or the number of events for exposed and unexposed groups that enable the calculation of a RR.

Outcomes consisted of incidence and/or mortality of seven types of cancer: Liver Cancer, Tracheal, Bronchus and Lung Cancer, Breast Cancer, Colon and Rectum Cancer, Pancreatic Cancer, Bladder Cancer and Ovarian Cancer. These are the seven cancers that GBD has ever quantified attributable burden from FPG and were identified in 2017^7^. To perform BoP analysis, there must be a minimum of three studies reporting on the relationship between exposure to the risk factor and development of the outcome. Ovarian cancer had initially been excluded from GBD 2021 FPG-cancer estimates due to insufficient data. However, as new studies were identified and allowed us to perform the analysis, it was included after PROSPERO registration. Studies were excluded if they were conducted on animals or including population that could bias the relative risk curves (e.g. subjects with previous cardiovascular or chronic kidney diseases or with a high risk for developing cancer independent of FPG levels), and studies that did not include data on incidence or mortality of one of the seven cancers selected for this analysis.

We used Distiller Systematic Review software (Evidence Partners Inc., Ottawa, Ont, Canada) to screen titles and abstracts and conduct full text analysis. In both phases, two independent reviewers assessed 10% of references in a consensus-building exercise to standardize the selection process and to obtain a minimum kappa coefficient of 0.9. A third reviewer who is the GBD expert in FPG or cancer estimation served to resolve any inconsistencies. The remaining references were single screened. Based on the results of the first 1000 titles and abstract articles that were screened, artificial intelligence was used to generate scores based on the probability of inclusion, and all studies with scores lower than 0.25 were automatically excluded. Potentially eligible references were assessed in full text to confirm eligibility, and then data were manually extracted using a standardized spreadsheet. In the case of different studies assessing the same cancer outcome and using data from the same cohort or database, the included study was selected based on three factors: (1) studies with continuous exposures were preferred over categorical exposures (e.g. FPG levels over DM diagnosis); (2) inclusion of cohorts with a wider age range of population; and (3) longer follow-up period. Extracted data included study characteristics such as range of years for which the cohort was being assessed, study design; characteristics of the population, including age, sex, and eligibility criteria; exposure and outcome measurements, with definitions and methods; the effect size, confidence interval, confounders considered in each study and other potential sources of study bias that correspond to BoP covariates included the statistical analysis. When multiple effect sizes were available and obtained from differently adjusted models, we extracted the fully adjusted one. A full list and description of all variables extracted is available on Table S4 of Supplementary Material.

### Exposure and outcome definitions

The standard exposure definition consisted of FPG as a continuum variable, in millimoles per liter (mmol/L), so studies providing HbA1c or plasma glucose after 2 h-OGTT were converted to FPG using FPG/HbA1c or FPG/OGTT as a conversion factor assuming both 6.5% HbA1c and 2 h plasma glucose of 11.1 mmol/L after OGTT as an equivalent to 7 mmol/L of FPG^165^. For studies with dichotomous data (DM vs. no DM) with no reported FPG thresholds, we assumed DM as FPG *≥* 7.0 mmol/L and no DM as FPG < 7 mmol/L^166^. If the lower or upper end of a categorical exposure range was not specified, we imputed values based on regional distribution of FPG values among individual-level microdata. Studies involving self-reported DM with no evidence of physician diagnosis, with type 1 diabetes and gestational diabetes were excluded, as well as those with measures of glycemic variability assessed by continuous glucose monitoring. Standard cancer definitions comprised reporting International Classification of Diseases (ICD) codes for each cancer type, available in Sect. 2.3 of Supplementary Material. Studies were not excluded if cancer diagnosis was self-reported.

### Estimating the shape of the exposure-relative risk relationship

A mean risk curve across the range of FPG was modeled using a meta-analytic tool, MR-BRT (Meta Regression—Bayesian, regularized, trimmed), for each cancer type. In this model, the risk for cancer incidence and mortality was the dependent variable and FPG was the continuous independent variable. To ensure that the risk association is robust to potential outliers in the data, a least trimmed squares (LTS) approach is applied, decreasing the potential of publication or reporting bias. As a result, risk associations were modeled with 10% of the relative risks trimmed as potential outliers.

### Testing and adjusting for biases across study designs and characteristics

Systematic sources of bias of each individual study were assessed as binary covariates based on the principles of Grading of Recommendations, Assessment, Development and Evaluations (GRADE) approach: (1) quality of exposure measurement (2) quality of outcome measurement; (3) representativeness of study population; (4) confounder quality; (5) risk of selection bias; and (6) risk of reverse causation. Exposure and outcome measurements were considered as “good quality” when defined based on objective measures (e.g. FPG levels for the exposure) or obtained from administrative medical records or disease registries. Representativeness was quantified by whether the study sample was geographically representative. Confounder quality was defined based on the adjusted variables in the studies and on GBD covariates for each cancer type. A good quality adjustment was considered when including age, sex, smoking, alcohol use and BMI for all types of cancers, and for breast cancer, family history of breast cancer was also considered. Detailed definitions of exposure, outcome and confounder quality are available on Supplementary Table S7. All covariates that significantly biased the estimated RR function were then included in the adjusted meta-regression models.

### Quantifying between-study heterogeneity

Between-study heterogeneity is the degree of variation in the RRs among studies and reflects consistency across results that can be affected by methodological and random variations between studies. Relevant study-level covariates were selected to account for heterogeneity through LASSO algorithm, and a final risk-outcome association was estimated using a linear mixed-effects model considering all these bias covariates. Remaining unexplained between-study heterogeneity was captured by gamma (γ) and contributes to the overall rating of effect size and evidence strength. Gamma (γ) was estimated using the Fisher Information Matrix, a method that is used to account for both data sparsity due to few studies and the presence of within-study correlation^13^. The 95% UIs of the final model represent the 2.5th and 97.5th quantiles with the between-study heterogeneity incorporated, so the unexplained variation in results that remained after accounting for study-level covariates contribute to lower precision, a lower risk score and lower star ratings.

### Evaluating publication bias

The potential of publication or reporting bias, even after trimming 10% of data, was detected by Egger’s Regression by a significant correlation between the mean RRs and their standard errors. P-values lower than 0.05 in Egger’s test in addition to an asymmetrical distribution of data in the funnel plot, which shows the residuals of the risk curve against their standard errors, were flagged as evidence potential of publication or reporting biases. If we found evidence of publication bias, it was reported but there was no correction to the risk function.

### Estimating the burden of proof risk functions (BPRF)

The BPRF is defined, in our study, as the 5th quantile curve closest to the line of relative risk equal to 1 (the null) and represents the smallest cancer risk at each level of FPG consistent with the available evidence. It was derived considering the uncertainty estimates addressing between-study heterogeneity described above. The mean risk curve, 95% uncertainty intervals (UIs) with and without between-study heterogeneity, and BPRF uses the midpoint of each exposure range. Those trimmed data points are marked with a red X.

To facilitate interpretation of the strength of evidence between each FPG-cancer pair, the ROS was generated from the average log (RR) of BPRF over the data-dense area of the observed exposure range, which consisted of the signed value of the log (BPRF) averaged between the 15th and 85th percentiles of observed exposure levels for each outcome. Higher positive ROSs correspond to stronger and consistent evidence that the exposure to higher levels of FPG increases the risk of cancer, while a negative ROS suggests that, under an analysis of the evidence, there is no significant association between levels of FPG and risk of a specific type of cancer.

### Model validation

The meta-analytical method used in this study has been previously validated by Zheng and colleagues^13^. The final model parameters were selected based on model fit to data and data availability at the lower and upper exposure ranges. Given the general lack of data at the higher exposure ranges of data, a right linear tail was used for all outcomes and, for those outcomes where the risk curves did not follow the data trends sufficiently, a cubic spline was used (e.g. pancreatic cancer). Given the large amount of data and the large existing heterogeneity, trimming 10% data points was necessary to avoid introducing biases from outlying studies. Risk curves were reviewed and discussed before final collective decisions were made. We also estimated the risk and 95% UI inclusive of between-study heterogeneity of the seven types of cancer at clinically meaningful FPG thresholds: (1) 6.1 mmol/L corresponding to prediabetes; and (2) 7.0 mmol/L corresponding to DM. Finally, to evaluate the robustness of our findings, we undertook sensitivity analyses including only studies with a prospective cohort design for all types of cancers.

## Supplementary Information

Below is the link to the electronic supplementary material.

Supplementary Material 1

## Data Availability

The findings from this study are supported by data from the published literature available in the Supplementary Material. Details on data sources can be found on the GHDx website (https://ghdx.healthdata.org/gbd-2021/sources) and further information can be requested in the GDHx contact form (https://ghdx.healthdata.org/contact) or directly contacting Dr. Liane Ong by the email ongl@uw.edu.
