# Supplementary material for: A burden of proof study of the effects of exposure to high fasting plasma glucose on the risk of seven types of cancer
Source: Sci Rep. 2025 Aug 7;15:28859. doi: 10.1038/s41598-025-13045-9 (PMC12331999; doi:10.1038/s41598-025-13045-9)
Supplement: Supplementary file 1 — Supplementary Material 1 [file 41598_2025_13045_MOESM1_ESM.docx]

Supplementary Material: data sources and supplementary results for “A burden of proof study of the effects of exposure to high fasting plasma glucose on the risk of seven types of cancer"

This appendix provides detailed information on input data sources and supplementary results for “A burden of proof study of the effects of exposure to high fasting plasma glucose on the risk of seven types of cancer."

#

Table of Contents

Summary

[Section 1: GATHER and PRISMA checklists 4](#_Toc170203403)

[Table S1. GATHER checklist 4](#_Toc170203404)

[Table S2. PRISMA 2020 checklist 5](#_Toc170203405)

[Section 2: Data source identification and assessment 7](#_Toc170203406)

[Section 2.1: Literature studies 7](#_Toc170203407)

[Section 2.2: Assessing data source eligibility 7](#_Toc170203409)

[Section 2.3: List of ICD-10 codes for cancer definition 8](#_Toc170203410)

[Section 2.3: Studies with high-risk population that were excluded 8](#_Toc170203411)

[Table S4. Causal criteria extraction template 10](#_Toc170203412)

[Section 3: Study characteristics included in the analysis between high FPG levels and the risk of each type of cancer 16](#_Toc170203413)

[Section 4: Study quality and bias covariates 58](#_Toc170203414)

[Table S6. Study quality for every study used in the models 58](#_Toc170203415)

[Table S7. Variables considered for defining good confounder quality for each cancer 58](#_Toc170203416)

[Section 5: Results from individual studies 58](#_Toc170203417)

[Table S8. Summary results from input studies 58](#_Toc170203418)

[Section 6: Main risk curves of outcomes that rated < 2 stars. 76](#_Toc170203419)

[Section 8: Sensitivity Analysis 78](#_Toc170203420)

[Table S9. Strength of the evidence for the relationship between FPG levels and the seven health outcomes analyzed, when including only studies with cohort design (prospective or retrospective). 78](#_Toc170203421)

[Table S10. Relative risks across exposure range 79](#_Toc170203422)

## Section 1: GATHER and PRISMA checklists

### Table S1. GATHER checklist

| Item # | Checklist item | Reported on page 18 |
| --- | --- | --- |
| Objectives and funding | | |
| 1 | Define the indicator(s), populations (including age, sex, and geographic entities), and time period(s) for which estimates were made. | Methods Section, page 18, 19 |
| 2 | List the funding sources for the work. | Page 34 |
| Data Inputs | | |
| For all data inputs from multiple sources that are synthesized as part of the study: | | |
| 3 | Describe how the data were identified and how the data were accessed. | Pages 19 |
| 4 | Specify the inclusion and exclusion criteria. Identify all ad-hoc exclusions. | Page 18, 19 |
| 5 | Provide information on all included data sources and their main characteristics. For each data source used, report reference information or contact name/institution, population represented, data collection method, year(s) of data collection, sex and age range, diagnostic criteria or measurement method, and sample size, as relevant. | Supplemental Information, Section 3 |
| 6 | Identify and describe any categories of input data that have potentially important biases (e.g., based on characteristics listed in item 5). | Supplemental Information, Tables S6 and S7 |
| For data inputs that contribute to the analysis but were not synthesized as part of the study: | | |
| 7 | Describe and give sources for any other data inputs. | N/A |
| For all data inputs: | | |
| 8 | Provide all data inputs in a file format from which data can be efficiently extracted (e.g., a spreadsheet rather than a PDF), including all relevant meta-data listed in item 5. For any data inputs that cannot be shared because of ethical or legal reasons, such as third-party ownership, provide a contact name or the name of the institution that retains the right to the data. | Data Availability, page 23 |
| Data analysis | | |
| 9 | Provide a conceptual overview of the data analysis method. A diagram may be helpful. | Methods Section, pages 21-23 |
| 10 | Provide a detailed description of all steps of the analysis, including mathematical formulae. This description should cover, as relevant, data cleaning, data pre-processing, data adjustments and weighting of data sources, and mathematical or statistical model(s). | Methods Section, pages 21-23 |
| 11 | Describe how candidate models were evaluated and how the final model(s) were selected. | Methods Section, page 23 |
| 12 | Provide the results of an evaluation of model performance, if done, as well as the results of any relevant sensitivity analysis. | Methods Section, page 23 |
| 13 | Describe methods for calculating uncertainty of the estimates. State which sources of uncertainty were, and were not, accounted for in the uncertainty analysis. | Methods Section, pages 21-23 |
| 14 | State how analytic or statistical source code used to generate estimates can be accessed. | Code Availability, Page 23 |
| Results and Discussion | | |
| 15 | Provide published estimates in a file format from which data can be efficiently extracted. | https://vizhub.healthdata.org/burden-of-proof/ |
| 16 | Report a quantitative measure of the uncertainty of the estimates (e.g. uncertainty intervals). | Results section, pages 6-12 |
| 17 | Interpret results in light of existing evidence. If updating a previous set of estimates, describe the reasons for changes in estimates. | Discussion section, pages 13,14 |
| 18 | Discuss limitations of the estimates. Include a discussion of any modelling assumptions or data limitations that affect interpretation of the estimates. | Discussion section, pages 15,16 |

### Table S2. PRISMA 2020 checklist

| **Section and Topic** | **Item #** | **Checklist item** | **Location where item is reported** |
| --- | --- | --- | --- |
| **TITLE** | | |  |
| Title | 1 | Identify the report as a systematic review. | Not in the title |
| **ABSTRACT** | | |  |
| Abstract | 2 | See the PRISMA 2020 for Abstracts checklist. | Page 2 |
| **INTRODUCTION** | | |  |
| Rationale | 3 | Describe the rationale for the review in the context of existing knowledge. | Main Section, page 4 |
| Objectives | 4 | Provide an explicit statement of the objective(s) or question(s) the review addresses. | Page 4 |
| **METHODS** | | |  |
| Eligibility criteria | 5 | Specify the inclusion and exclusion criteria for the review and how studies were grouped for the syntheses. | Methods Section, pages 17,18 |
| Information sources | 6 | Specify all databases, registers, websites, organisations, reference lists and other sources searched or consulted to identify studies. Specify the date when each source was last searched or consulted. | Methods section, pages 17,18 |
| Search strategy | 7 | Present the full search strategies for all databases, registers and websites, including any filters and limits used. | Supplemental Information, page 5 |
| Selection process | 8 | Specify the methods used to decide whether a study met the inclusion criteria of the review, including how many reviewers screened each record and each report retrieved, whether they worked independently, and if applicable, details of automation tools used in the process. | Methods section, pages 18,19 |
| Data collection process | 9 | Specify the methods used to collect data from reports, including how many reviewers collected data from each report, whether they worked independently, any processes for obtaining or confirming data from study investigators, and if applicable, details of automation tools used in the process. | Methods Section, page 19,20 |
| Data items | 10a | List and define all outcomes for which data were sought. Specify whether all results that were compatible with each outcome domain in each study were sought (e.g. for all measures, time points, analyses), and if not, the methods used to decide which results to collect. | Methods Section, page 19,20 |
|  | 10b | List and define all other variables for which data were sought (e.g. participant and intervention characteristics, funding sources). Describe any assumptions made about any missing or unclear information. | Methods Section, pages 19,20 and Supplementary Table S4 |
| Study risk of bias assessment | 11 | Specify the methods used to assess risk of bias in the included studies, including details of the tool(s) used, how many reviewers assessed each study and whether they worked independently, and if applicable, details of automation tools used in the process. | Methods Section, page 21 |
| Effect measures | 12 | Specify for each outcome the effect measure(s) (e.g. risk ratio, mean difference) used in the synthesis or presentation of results. | Methods Section, page 18 |
| Synthesis methods | 13a | Describe the processes used to decide which studies were eligible for each synthesis (e.g. tabulating the study intervention characteristics and comparing against the planned groups for each synthesis (item #5)). | Methods Section, pages 18-20 |
|  | 13b | Describe any methods required to prepare the data for presentation or synthesis, such as handling of missing summary statistics, or data conversions. | Methods Section, pages 18,19 |
|  | 13c | Describe any methods used to tabulate or visually display results of individual studies and syntheses. | Supplementary Information, Section 3 |
|  | 13d | Describe any methods used to synthesize results and provide a rationale for the choice(s). If meta-analysis was performed, describe the model(s), method(s) to identify the presence and extent of statistical heterogeneity, and software package(s) used. | Methods Section, pages 19-21 |
|  | 13e | Describe any methods used to explore possible causes of heterogeneity among study results (e.g. subgroup analysis, meta-regression). | Methods section, page 21 |
|  | 13f | Describe any sensitivity analyses conducted to assess robustness of the synthesized results. | Methods Section, page 23 |
| Reporting bias assessment | 14 | Describe any methods used to assess risk of bias due to missing results in a synthesis (arising from reporting biases). | Methods Section, page 22 |
| Certainty assessment | 15 | Describe any methods used to assess certainty (or confidence) in the body of evidence for an outcome. | Methods Section, page 22 |
| **RESULTS** | | |  |
| Study selection | 16a | Describe the results of the search and selection process, from the number of records identified in the search to the number of studies included in the review, ideally using a flow diagram. | Results Section, pages 4,5 |
|  | 16b | Cite studies that might appear to meet the inclusion criteria, but which were excluded, and explain why they were excluded. | Results Section, pages 4,5 |
| Study characteristics | 17 | Cite each included study and present its characteristics. | Supplemental Information, Section 3 |
| Risk of bias in studies | 18 | Present assessments of risk of bias for each included study. | Not available |
| Results of individual studies | 19 | For all outcomes, present, for each study: (a) summary statistics for each group (where appropriate) and (b) an effect estimate and its precision (e.g. confidence/credible interval), ideally using structured tables or plots. | Results Section, pages 4-10 |
| Results of syntheses | 20a | For each synthesis, briefly summarise the characteristics and risk of bias among contributing studies. | Not available |
|  | 20b | Present results of all statistical syntheses conducted. If meta-analysis was done, present for each the summary estimate and its precision (e.g. confidence/credible interval) and measures of statistical heterogeneity. If comparing groups, describe the direction of the effect. | Results Section, pages 5-13 and Table 1 |
|  | 20c | Present results of all investigations of possible causes of heterogeneity among study results. | Supplementary Information, Section 8 |
|  | 20d | Present results of all sensitivity analyses conducted to assess the robustness of the synthesized results. | Supplementary Figures, Section 8 and Results Section, pages 5-13 |
| Reporting biases | 21 | Present assessments of risk of bias due to missing results (arising from reporting biases) for each synthesis assessed. | Figures 1 to 3, and Supplementary Information, Section 8 |
| Certainty of evidence | 22 | Present assessments of certainty (or confidence) in the body of evidence for each outcome assessed. | Results Section, pages 5-13, Tables 1 and 2 |
| **DISCUSSION** | | |  |
| Discussion | 23a | Provide a general interpretation of the results in the context of other evidence. | Discussion Section, pags 13,14 |
|  | 23b | Discuss any limitations of the evidence included in the review. | Discussion Section, pages 15, 16 |
|  | 23c | Discuss any limitations of the review processes used. | Discussion Section, pages 15, 16 |
|  | 23d | Discuss implications of the results for practice, policy, and future research. | Discussion Section, page 16, 17 |
| **OTHER INFORMATION** | | |  |
| Registration and protocol | 24a | Provide registration information for the review, including register name and registration number, or state that the review was not registered. | Methods section, page 18 |
|  | 24b | Indicate where the review protocol can be accessed, or state that a protocol was not prepared. | Methods section, page 18 |
|  | 24c | Describe and explain any amendments to information provided at registration or in the protocol. | Methods section, page 18 |
| Support | 25 | Describe sources of financial or non-financial support for the review, and the role of the funders or sponsors in the review. | Acknowledgements, Page 36 |
| Competing interests | 26 | Declare any competing interests of review authors. | Page 36 |
| Availability of data, code and other materials | 27 | Report which of the following are publicly available and where they can be found: template data collection forms; data extracted from included studies; data used for all analyses; analytic code; any other materials used in the review. | Page 23 |

## Section 2: Data source identification and assessment

### Section 2.1: Literature studies

Literature search was conducted to obtain data from observational studies assessing the relationship between high fasting plasma glucose and seven types of cancer: ovarian, bladder, breast, lung, pancreatic, liver, and colorectal cancer. The original search was performed in 2019, and the search was updated to include studies published between January, 2020 and September, 2023. The search string was developed for PubMed, as follows:

#### Section 2.1.1: PubMed search

### (((((diabetes[MeSH Terms] OR diabetes[Title/Abstract] OR hyperglycemia[MeSH Terms] OR hyperglycemia[Title/Abstract] OR blood glucose[MeSH Terms] OR blood glucose[Title])) AND (Case-Control Studies[MeSH Terms] OR Cross-Over Studies[MeSH Terms] OR Cohort Studies[MeSH Terms] OR Systematic Review[Publication Type] OR Meta-Analysis[Publication Type] OR systematic review[Title/Abstract] OR Meta-analysis[Title/Abstract] OR cohort[Title/Abstract] OR cross-over[Title/Abstract] OR crossover[Title/Abstract] OR case-control[Title/Abstract] OR prospective[Title/Abstract] OR retrospective[Title/Abstract] OR longitudinal[Title/Abstract] OR follow-up[Title/Abstract] OR Dose-Response Relationship, Drug[MeSH Terms] OR dose-response[Title/Abstract]) AND (Risk[MeSH Terms] OR Odds Ratio[MeSH Terms] OR risk[Title/Abstract] OR odds ratio[Title/Abstract] OR cross-product ratio[Title/Abstract] OR hazards ratio[Title/Abstract] OR hazard ratio[Title/Abstract])) NOT (animals[MeSH Terms] NOT Humans[MeSH Terms])))) AND (cancer[MeSH Terms] OR neoplasm[MeSH Terms] OR cancer[Title/Abstract] OR neoplasm[Title/Abstract])

### Section 2.2: Assessing data source eligibility

See Figure S1 below for details on identifying, screening, and assessing eligibility for records identified through our search.

Studies were included if they:

- Were prospective and retrospective cohorts, case-control, nested case-controls or meta-analysis/systematic reviews;
- Included general representative population or if within specific populations, selection criteria must not include factors considered to be associated with high FPG or the specific cancer;
- Showed at least one of the following fasting plasma glucose measures (serum or blood glucose), HbA1c or plasma glucose after 2h-OGTT, frequency of diabetes as a whole or type 2 diabetes based on ICD codes or doctor diagnosis;
- Cancer outcomes of interest based on ICD code mappings or self-reported cancer;
- Showed a risk measure such as incidence rate ratio, hazard ratio, relative risk, odds ratio or standard mortality ratio.

Studies were excluded if they:

- Cross-sectional, case studies, narrative reviews or pooled cohorts;
- Included only people that are at high risk for developing the type of cancer being assessed;
- Compared type 1 diabetes vs non type 1 or vs no diabetes
- Diabetes was defined by self-reported, without any suggestion of medical diagnosis (i.e “Do you have diabetes?”);
- Reported cancer outcomes not of interest (i.e overall survival, prognosis, etc);

### Section 2.3: List of ICD-10 codes for cancer definition

| GBD Cancer Definition | ICD-10 codes |
| --- | --- |
| Bladder Cancer | C67.0 – C67.9 |
| Breast Cancer | C50.0 - C50.929 |
| Colon and Rectum Cancer | C18.0 – C21.9 |
| Liver Cancer | C22.0 – C22.9 |
| Pancreas Cancer | C25.0 – C25.9 |
| Tracheal, Bronchus and Lung | C34.0 – C34.9 |
| Ovarian Cancer | C56.0 – C56.9 |

### Section 2.3: Studies with high-risk population that were excluded

| **Population - Characteristics not described as covariates** | **Cancer type** |
| --- | --- |
| Patients with history of Helicobacter pylori infection | Colorectal |
| Patients with inflammatory bowel disease | Colorectal |
| Patients with primary sclerosing cholangitis and primary biliary cholangitis | Liver |
| Patients with NAFLD | Liver |
| Patients with type-1 Hereditary hemocromatosis genotypes (C282Y/C282Y or C282Y/H63D) | Liver |
| Hereditary hemochromatosis patients | Liver |
| Patients with COPD | Tracheal, Bronchus and Lung |
| Patients with chronic pancreatitis | Pancreas |
| Patients with 45 autoimmune conditions | Pancreas |

**Figure S1. PRISMA 2020 flow diagram for updated systematic reviews which included searches of databases, registers and other sources**

**Identification of new studies via other methods**

**Identification of new studies via databases and registers**

**Previous studies**

Records obtained from the ***23 identified metanalyses** (n= 910)

No records removed *before screening*

Records identified from:

PubMed (n = 4098)

Studies included in the previous search

(n = 113)

**Identification**

Records excluded (n = 3815)

Artificial Intelligence (n=2642)

Biased/Unrepresentative population (n = 215)

Irrelevant focus/exposure (N=556)

Non-cancer outcomes (N=236)

Other cancer outcomes (N=102)

Irrelevant study design (N=35)

Missing dose response (N=24)

Others (N=4)

GBD study (n=1)

Records screened (n = 4098)

Records not retrieved (n=846)

Published before 2020 (n=845)

Article not found (n=1)

Records sought for retrieval (n = 910)

Records not retrieved (n=2)

Records sought for retrieval

(n = 283)

**Screening**

Records excluded (n=253):

Biased/Unrepresentative population (n = 42)

Irrelevant focus/exposure (N=47)

Non-cancer outcomes (N=17)

Other cancer outcomes (N=53)

Irrelevant study design (N=8)

Missing dose response (N=10)

Missing Uncertainty (N=1)

Others (N=13)

Studies using data from the same cohort (n=22)

Self-reported Type 2 diabetes and Type 1 Diabetes (n=17)

***Metanalysis (n=23)**

Records assessed for eligibility (n = 281)

Records reassessed for eligibility (n=113)

Reports excluded (n=58)

Biased/Unrepresentative population (n =4)

Irrelevant focus/exposure (N=27)

Non-cancer outcomes (N=2)

Other cancer outcomes (N=10)

Duplicates (n=6)

Already included through the search (n=5)

Self-reported diabetes (n=1)

Studies using data from the same cohort (n=3)

Reports assessed for eligibility (n = 64)

Number of studies included from the previous search (n=78)

Number of studies included from the new search (n=63)

Number of studies included from metanalysis (n=6)

**Included**

Total studies included in review (n= 147)

For studies that met the inclusion criteria, data were extracted for the variables listed in the following Table S4.

### Table S4. Causal criteria extraction template

| **NID** | Article identification in GHDx library |
| --- | --- |
| **acause** | Cancer identification according to GBD cause list (E.g neo_pancreas) |
| **sex** | Both, Male or Female |
| **measure** | Description of effect measure (E.g. hazard ratio, relative risk, odds ratio) |
| **location_name** | Study location based on GBD location ID tables |
| **location_id** | Code for GBD location according to ID Tables |
| **year_start** | Year of start of data collection (not necessarily the same of the cohort) |
| **year_end** | Year of end of data collection (not necessarily the same of the cohort) |
| **age_start** | Lower bound of age range (E.g from 40 to 79 years). If no data, we assumed 18 |
| **age_end** | Upper bound of age range (E.g from 40 to 79 years). If no description, we assumed 99 |
| **age_mean** | Mean age of the entire sample |
| **age_sd** | Standard deviation associated with mean age of study sample |
| **design** | According to paper description (prospective, retrospective cohot, case-control, nested case-control) |
| **mean** | Mean effect size |
| **effect_size_measure** | Measure of effect (hazard ratio, odds ratio, incident rate ratio, relative risk) |
| **lower** | Lower bound of confidence interval of the mean effect size |
| **upper** | Upper bound of confidence interval of the mean effect size |
| **cov_representativeness** | Whether a study sample is representative of general population  0: representative of general population  1:  not |
| **cov_exposure_quality** | Quality of exposure definition  0: individual, objective measure, multiple prospective measures  1: population method of reporting, self-report, single baseline prospective measurement |
| **cov_outcome_quality** | Quality of outcome definition  0: Death record/medical record/physician DX  1: self-report |
| **cov_confounder_quality** | See confounder quality table below for cause specific coding |
| **cov_reverse_causation** | Potential for outcome causing the risk factor  0: low potential  1: high potential |
| **cov_selection_bias** | Percentage of study population retained in follow-up  0: 95% or more  1: 85-94%  2: 75-84%  3: less than 75% |
| **notes_exposure_def** | Free text notes on exposure definition |
| **notes_outcome_def** | Free text notes on outcome definition (Can input cancer subtypes) |
| **notes_exposed_group_def** | Free text notes on exposed group definition |
| **notes_unexposed_reference_group_def** | Free text notes on unexposed group definition |
| **notes_general** | Free text notes (General issues, not specific to exposure or outcome) |
| **is_outlier** | Column to flag outliered studies |
| **custom_label_no.** | Column to identify extraction source. All studies from the original search were gbd2019 and from the updated search were gbd2022. |
| **custom_PMID** | PubMed PMID if available |
| **field_citation_value** | Article citation - Use Zotero standard formatting |
| **outcome_alignment** | Outcome ICD code definition based on GBD cause list   - Exact = study used the same ICD codes as GBD code list - Aggregate = study added codes other than GBD code list - Component = study used less codes than GBD list |
| **cov_rep_geography** | Were the study participants representative of the geography?   0 = yes  1 = no |
| **rep_prevalent_disease** | Is the study aiming to evaluate the risk or mortality of people who have already developed the outcome?  1 = mortality  0 = incidence |
| **custom_washout** | If there was a washout period?  0 = no 1 = yes  (E.g. the cohort started in 2000, but the study considered only data collected after 2002; or “excluded the first 3 years of outcomes…)” |
| **age_issue** | Age issues include assuming age start/end, calculating mean age etc  0 = no  1 = yes |
| **percent_male** | Percent of males (number from 0 to 1) |
| **sex_issue** | Any issues related to sex  1= yes 0 = no (If yes, describe why in notes_sr column) |
| **study_name** | Name of the study (e.g., Nurses' Health Study), if provided.  If not available, left blank. |
| **exp_instrument** | Free text of how the exposure was assessed. For self-reported exposures, the name of the questionnaire. |
| **exp_assess_period** | Frequency of exposure assessment  "only at baseline" or  "multiple times at follow-up" |
| **exp_assess_num** | If multiple exposure assessments, the number of times should be specified |
| **exp_method** | Method of exposure assessment. |
| **exp_type** | Which form of the exposure was included in relative risk estimation analysis? "Baseline" or "Cumulative average" |
| **outcome_def** | Outcome definition: Brief description of the outcome as reported in the study. |
| **outcome_type** | Incidence or mortality |
| **outcome_assess_1** | Method of assessment of the study outcome |
| **duration_fup_units** | Units of follow up duration (days, months, years) |
| **duration_fup_measure** | Measure of follow up duration (mean, median, range) |
| **value_of_duration_fup** | Duration of follow-up |
| **confounders_age** | Was the analysis adjusted for age? 0 = no 1 = yes |
| **confounders_sex** | Was the analysis adjusted for sex? 0 = no 1 = yes |
| **confounders_education** | Was the analysis adjusted for education? 0 = no 1 = yes |
| **confounders_income** | Was the analysis adjusted for income? 0 = no 1 = yes |
| **confounders_smoking** | Was the analysis adjusted for smoking status, packs-year? 0 = no 1 = yes |
| **confounders_alcohol_use** | Was the analysis adjusted for alcohol use, grams of alcohol, or anything related to alcohol? 0 = no 1 = yes |
| **confounders_physical_activity** | Was the analysis adjusted for physical activity, exercise, or anything related to PA? 0 = no 1 = yes |
| **confounders_dietary_components** | Was the analysis adjusted for any dietary component or index? 0 = no 1 = yes |
| **confounders_bmi** | Was the analysis adjusted for BMI? 0 = no 1 = yes |
| **confounders_hypertension** | Was the analysis adjusted for hypertension, blood pressure or antihypertensive medication? 0 = no 1 = yes |
| **confounders_diabetes** | Was the analysis adjusted for presence of diabetes, use of antidiabetic medication or insulin? 0 = no 1 = yes |
| **confounders_hypercholesterolemia** | Was the analysis adjusted for presence of dyslipidemia, total or LDL or HDL-cholesterol, or triglycerides? 0 = no 1 = yes |
| **confounders_familyhistory** | Was the analysis adjusted for family history of cancer? 0 = no 1 = yes |
| **confounders_other** | Description of all other confounders |
| **page_num_effect_size** | Page and table number from which the results were extracted |
| **subgroup_analysis** | Is this result of a subgroup analysis (sex or age subgroup)?  0 = no  1 = yes |
| **effect_size_multi_location** | Is this a multicenter study?  0=no  1=yes |
| **cohort_person_years_exp** | Number of persons-year in exposed group |
| **cohort_person_years_unexp** | Number of persons-year in unexposed group |
| **cohort_person_years_total** | Number of total persons-year |
| **cohort_number_events_exp** | Number of cancer events in exposed group |
| **cohort_number_events_unexp** | Number of cancer events in unexposed group |
| **cohort_number_events_total** | Total number of events |
| **cohort_sample_size_exp** | Exposed group sample size |
| **cohort_sample_size_unexp** | Unexposed group sample size |
| **cohort_sample_size_total** | Total sample size |
| **cohort_exposed_def** | Brief description of the exposed group as used in estimation of the relative risk (E.g. diabetes type 2) |
| **glucose_test** | Indicates the glucose test used for exposure   - Diabetes if yes/no - FPG - HbA1C - OGTT - If a combination, input with a “,” as separation: mainly "FPG" or "HbA1c" or "HbA1c, FPG" or "OGTT" or "OGTT, diabetes" |
| **bias_miss_upper_bound_exp** | Is the upper bound missing?  1 = study with no upper bound on exposed group (E.g subjects were considered as having diabetes if FPG > 126 mg/dL) |
| **bias_miss_lower_bound_exp** | Is the lower bound missing  1=study with no lower bound for exposed group  0=study reported lower bound for exposed group |
| **b_0** | Extracted lower bound alternative group |
| **b_1** | Extracted upper bound alternative group  (If diabetes yes/no, it was kept blank) |
| **bias_miss_upper_bound_unexp** | 1 = Study with no upper bound for unexposed group  0 = study reported upper bound for unexposed group |
| **bias_miss_lower_bound_unexp** | 1 = Study with no lower bound for unexposed group  0 = study reported lower bound for unexposed group (E.g subjects were considered as normal FPG if < 99 mg/dL) |
| **a_0** | Extracted lower bound reference group |
| **a_1** | Extracted upper bound reference group (If diabetes yes/no, keep it blank) |
| **cohort_exp_unit_rr** | Unit of exposure exposed group:  mg/dL, mmol/L, %, mmol/mol (If diabetes yes/no, data imputed in mmol/L) |
| **cohort_unexp_def** | Brief description of the unexposed group as used in estimation of the relative risk (E.g. undiabetic, normoglycemia, normal fasting plasma glucose) |
| **cohort_unexp_unit_rr** | Unit of exposure unexposed group:  mg/dL, mmol/L, %, mmol/mol (If diabetes yes/no, data imputed in mmol/L) |
| **cc_community** | Were the controls selected from the community?  0 = no  1 = yes |
| **cc_cases** | Number of cancer cases in case-control studies |
| **cc_control** | Number subjects in control group in case-control studies |
| **cc_exposed_def** | Brief description of the exposed group as used in estimation of the relative risk (E.g. diabetes, hyperglycemia, high fasting plasma glucose) |
| **cc_exp_unit_rr** | Unit of exposure  mg/dL, mmol/L, %, mmol/mol (If diabetes yes/no, imput data in mmol/L) |
| **cc_exp_level_rr** | If continuous exposure in the article, description the exposure level in the exposed group. Specified the mean/median level of exposure in the exposed group. |
| **cc_unexposed_def** | Brief description of the unexposed group as used in estimation of the relative risk (E.g. undiabetic, normoglycemia, normal fasting plasma glucose) |
| **cc_unexp_level_rr** | Exposure level in the exposed group (for continuous risks): Specify the mean/median level of exposure in the unexposed group. |
| **note_modeler** | Free text about the models |
| **note_sr** | Space to flag all assumptions and issues during extraction |
| **extractor** | Username of person who extracted the data |
| **bias_exp_duration** | Bias covariate indicating that the study   1. Consider only subjects with an specific duration of diabetes (e.g having diabetes for at least 2 years) 2. Stratified diabetes based on time since diabetes diagnosis   Input 1=yes 0=no |
| **other_cancer** | Description of all other types of cancer available in the paper but not considered for this analysis |
| **flag** | Major issues that should be flagged |
| **subgroup** | Free text to note any subgroups |
| **bc_imputed_fpg** | Bias covariate indicating if data point had upper and/or lower exposure bound imputed because missing (all studies with diabetes yes/no should be filled with 1) |
| **bc_hba1c** | Bias covariate indicating if data point exposure range is in HbA1c values: Input: 1=yes, 0=no |
| **bc_ogtt** | Bias covariate indicating if data point exposure range is in OGTT values: Input: 1=yes, 0=no |
| **bc_exclude_known_dm** | Bias covariate indicating if study excluded people with known/previously diagnosed diabetes from analysis 1=yes, 0=no |
| **bc_tx** | Bias covariate indicating if exposure is defined based on diabetes status and the case definition used includes treatment as part of criteria. Input: 1=yes, 0=no |
| **bc_self_report** | Bias covariate indicating if exposure is defined based on self report diabetes with no suggestion of doctor diagnosis (was decided that these data points should not be included) Input: 1=yes, 0=no |
| **bc_dr_diag** | Bias covariate indicating if exposure is defined based on self report diabetes diagnosed by a doctor/physician ("Has a doctor ever diagnosed you with diabetes”) Input: 1=yes, 0=no |
| **bc_medical_records_dm** | bias covariate indicating if exposure is defined based on diabetes status and the case definition used identifies diabetes cases based on medical records. Input: 1=yes, 0=no |
| **bc_multipletest** | Bias covariate indicating multiple glucose test measures (E.g Was considered as having diabetes those with FPG > 126 mg/dL **and/or** Hba1c > 6.5% **and/or** 2h plasma glucose after OGTT > 11.1 mmol/L)  1=yes 0=no |

## Section 3: Study characteristics included in the analysis between high FPG levels and the risk of each type of cancer

3.1 Bladder Cancer

| Author | Year of publication | Study Name | Location | Study Design | Sex | Follow-up | Age Start | Age End | Exposure Measure | Endpoint | Disease Ascertainment | Events | Sample Size | Cases | Controls |
| --- | --- | --- | --- | --- | --- | --- | --- | --- | --- | --- | --- | --- | --- | --- | --- |
| Jee SH | 2005 | Korean Cancer Prevention Study | Republic of Korea | prospective cohort | Male | 10 years | 30 | 95 | FPG | Mortality | Administrative medical records or disease registries; ; | NI | 829770 | NA | NA |
|  | 2005 | Korean Cancer Prevention Study | Republic of Korea | prospective cohort | Male | 10 years | 30 | 95 | FPG | Incidence | Administrative medical records or disease registries; ; | NI | 829770 | NA | NA |
| Chung H | 2009 | Taiwan MJ Cohort study | Taiwan (Province of China) | case-cohort | Male | 10 years | 40 | 80 | FPG | Mortality | Administrative medical records or disease registries; ; | 22 | 54751 | NA | NA |
| Kuriki K | 2007 |  | Japan | case-control | Male | NI | 40 | 80 | Diabetes | Incidence | Administrative medical records or disease registries; ; | 75 | 14274 | 75 | 14199 |
| Wotton CJ | 2011 | ORLS1 | United Kingdom | prospective cohort | Both | NI | 30 | 99 | Diabetes | Incidence & Mortality | Administrative medical records or disease registries; Death certificates; | 1672 | 291462 | NA | NA |
|  | 2011 | ORLS2 | United Kingdom | prospective cohort | Both | NI | 30 | 99 | Diabetes | Incidence & Mortality | Administrative medical records or disease registries; Death certificates; | 619 | 192894 | NA | NA |
| Ogunleye AA | 2009 |  | Scotland | prospective cohort | Both | 3.9 years | 25 | 99 | Diabetes | Incidence & Mortality | Administrative medical records or disease registries; Death certificates; | 68 | 28731 | NA | NA |
| Tseng C-H | 2009 |  | Taiwan (Province of China) | prospective cohort | Both | 3 years | 18 | 99 | Diabetes | Incidence | Administrative medical records or disease registries; ; | NI | 998947 | NA | NA |
| Atchison EA | 2011 |  | United States of America | prospective cohort | Male | 10.5 years | 18 | 100 | Diabetes | Incidence & Mortality | Administrative medical records or disease registries; ; | 19390 | 371810 | NA | NA |
| Lo S-F | 2013 |  | Taiwan (Province of China) | prospective cohort | Both | 3.5 years | 25 | 99 | Diabetes | Incidence & Mortality | Administrative medical records or disease registries; ; | 4311 | 1790868 | NA | NA |
| Kravchick S | 2001 |  | Israel | case-control | Both | NI | 41 | 90 | Diabetes | Incidence | Administrative medical records or disease registries; ; | 16 | 51 | 16 | 35 |
| Ng Y | 2003 |  | United Kingdom | case-control | Both | NI | 60 | 99 | Diabetes | Incidence | Administrative medical records or disease registries; ; | 134 | 214 | 134 | 80 |
| MacKenzie T | 2011 |  | New Hampshire | case-control | Both | NI | 25 | 74 | Diabetes | Incidence | Self-report; Administrative medical records or disease registries; | 331 | 594 | 331 | 263 |
| Attner B | 2012 |  | Sweden | case-cohort | Both | 4 years | 45 | 84 | Diabetes | Incidence | Administrative medical records or disease registries; ; | 1123 | 167080 | NA | NA |
| Risch HA | 1988 |  | Canada | case-control | Both | NI | 35 | 79 | Diabetes | Incidence | Administrative medical records or disease registries; ; | 826 | 1618 | 826 | 792 |
| Chen HF | 2015 |  | Taiwan (Province of China) | case-control | Male | 8.9 years | 18 | 99 | Diabetes | Incidence | Administrative medical records or disease registries; ; | 2380 | 591129 | NA | NA |
|  |  |  |  |  | Female |  | 18 | 99 | Diabetes | Incidence | Administrative medical records or disease registries; ; | 1504 | 638618 | NA | NA |
| Tseng CH | 2009 |  | Taiwan (Province of China) | prospective cohort | Male | 12 years | 25 | 99 | Diabetes | Mortality | Administrative medical records or disease registries; ; | 76 | 40820 | NA | NA |
|  |  |  |  |  | Female | 12 years | 25 | 99 | Diabetes | Mortality |  | 49 | 47874 | NA | NA |
| Swerdlow AJ | 2005 | Diabetes UK cohort | United Kingdom | prospective cohort | Both | NI | 30 | 49 | Diabetes | Mortality | Administrative medical records or disease registries; ; | 3 | 5066 | NA | NA |
|  |  |  |  |  |  |  | 30 | 49 | Diabetes | Incidence | Administrative medical records or disease registries | 20 | 5066 | NA | NA |
| Coughlin SS | 2004 | Cancer Prevention Study II | United States of America | prospective cohort | Male | 16 years | 30 | 99 | Diabetes | Mortality | Administrative medical records or disease registries; Death certificates; | 1359 | 1056243 | NA | NA |
| Wideroff L | 1997 |  | Denmark | prospective cohort | Both | NI | 18 | 99 | Diabetes | Incidence | Administrative medical records or disease registries; ; | 493 | 109581 | NA | NA |
| Verlato G | 2003 | Verona Diabetes study | Italy | prospective cohort | Male | 10 years | 25 | 99 | Diabetes | Mortality | Administrative medical records or disease registries; Death certificates; | 22 | 3366 | NA | NA |
|  |  |  |  |  | Female | 10 years | 25 | 99 | Diabetes | Mortality | Administrative medical records or disease registries; Death certificates; | 6 | 3782 | NA | NA |
| Liu X | 2012 |  | Sweden | prospective cohort | Both | 7 years | 40 | 99 | Diabetes | Mortality | Administrative medical records or disease registries; ; | 294 | 849 | NA | NA |
| Ulcickas Yood M | 2009 |  | United States of America | retrospective cohort | Both | 4 years | 18 | 99 | Diabetes | Incidence | Administrative medical records or disease registries; ; | 243 | 191223 | NA | NA |
| Kim SK | 2020 |  | Republic of Korea | retrospective cohort | Both | 8.6 years | 30 | 99 | Diabetes | Incidence | Administrative medical records or disease registries; ; | NI | 25,709,497 | NA | NA |
| Choi YH | 2022 | Korean National Health Insurance System | Republic of Korea | prospective cohort | Both | 7.3 years | 18 | 99 | FPG | Incidence | Medical records; ; | 13769 | 9773462 | NA | NA |
| Barone, B | 2023 |  | Italy | retrospective cohort | Both | 2.11 years | 18 | 99 | Diabetes | Incidence | Medical records; Self-report; | 1638 | 2050 | NA | NA |
| Yilmaz Kavcar SR | 2022 | Balcova Heart Study (BHS) | Turkey | prospective cohort | Male | 6 years | 30 | 99 | FPG | Incidence | Cancer registries; ; | 16 | 15869 | NA | NA |
|  |  |  |  |  | Female | 6 years | 30 | 99 | FPG | Incidence | Cancer registries; ; | 9 | 15869 | NA | NA |
| Pradhan R | 2022 | Clinical Practice Research Datalink (CPRD) | United Kingdom | prospective cohort | Both | 8.4 years | 18 | 99 | Diabetes | Incidence | National database; ; | 10408 | 1780428 | NA | NA |
| Vicentini M | 2022 | Diabetes Registry and Population-based Cancer Registry | Italy | prospective cohort | Both | NI | 20 | 84 | Diabetes | Incidence | Cancer registries; ; | 1062 | 401051 | NA | NA |
| Gurney J | 2022 | Statistics New Zealanda's Integrated Data Infrastructure (IDI) | New Zealand | prospective cohort | Both | NI | 18 | 99 | Diabetes | Incidence | Cancer registries; ; | 3633 | NI | NA | NA |
| Campbell PT | 2022 | Cancer Prevention Study-II (CPS-II) Nutrition Cohort | United States of America | case-control | Both | 10.4 years | 18 | 99 | HbA1C | Incidence | Cancer registries; medical records; | 108 | 870 | 108 | 762 |
| Hu Y | 2021 | Health Professionals Follow-up Study | United States of America | prospective cohort | Male | NI | 40 | 75 | FPG | Incidence | Medical records; ; | 781 | 45604 | NA | NA |
|  |  | Nurses Health Study (NHS) |  |  | Female | NI | 30 | 55 | FPG | Incidence | Medical records; ; | 637 | 113429 | NA | NA |
| Park SH | 2023 |  | New Zealand | prospective cohort | Both | NI | 18 | 99 | Diabetes | Incidence | Cancer registries; ; | 3579 | 4236350 | NA | NA |
| Bogumil D | 2023 | Multiethnic Cohort Study (MEC) | United States of America | prospective cohort | Both | 19.7 years | 45 | 75 | Diabetes | Incidence | Cancer registries; ; | 1890 | 185059 | NA | NA |
| Bjornsdottir HH | 2020 | The Swedish National Diabetes Register (NDR) | Sweden | prospective cohort | Both | 6.6 years | 18 | 99 | Diabetes | Incidence | Death registries; ; | NI | 2744838 | NA | NA |
|  |  |  |  |  |  |  | 18 | 99 | Diabetes | mortality | Death registries; ; | NI | 2744838 | NA | NA |
| Peila R | 2020 | UK Biobank cohort | United Kingdom | prospective cohort | Both | 7.1 years | 40 | 69 | HbA1C | Incidence | Medical records; National Database; | 695 | 476517 | NA | NA |

3.2 Colorectal Cancer

| Author | Year of publication | Study Name | Location | Study Design | Sex | Follow-up | Age Start | Age End | Exposure Measure | Endpoint | Disease Ascertainment | Events | Sample Size | Cases | Controls |
| --- | --- | --- | --- | --- | --- | --- | --- | --- | --- | --- | --- | --- | --- | --- | --- |
| Jee SH | 2005 | Korean Cancer Prevention Study | Republic of Korea | prospective cohort | Male | 10 years | 30 | 95 | FPG | Mortality | Administrative medical records or disease registries; ; | NI | 829770 | NA | NA |
|  | 2005 | Korean Cancer Prevention Study | Republic of Korea | prospective cohort | Female | 10 years | 30 | 95 | FPG | Mortality | Administrative medical records or disease registries; ; | NI | 468615 | NA | NA |
|  | 2005 | Korean Cancer Prevention Study | Republic of Korea | prospective cohort | Male | 10 years | 30 | 95 | FPG | Incidence | Administrative medical records or disease registries; ; | NI | 829770 | NA | NA |
|  | 2005 | Korean Cancer Prevention Study | Republic of Korea | prospective cohort | Female | 10 years | 30 | 95 | FPG | Incidence | Administrative medical records or disease registries; ; | NI | 468615 | NA | NA |
| Limburg PJ | 2006 | ATBC study cohort | Finland | case-cohort | Male | 9 years | 50 | 69 | FPG | Incidence | Administrative medical records or disease registries; Physician diagnosis; | 134 | 29133 | NA | NA |
| Kabat GC | 2012 | Women's Health Initiative | United States of America | prospective cohort | Female | 12 years | 50 | 79 | FPG | Incidence | Self-report; Administrative medical records or disease registries; | 81 | 4902 | NA | NA |
| Wulaningsih W | 2012 | AMORIS study | Sweden | prospective cohort | Both | 12 years | 20 | 99 | FPG | Incidence & Mortality | Administrative medical records or disease registries; ; | 2810 | 540309 | NA | NA |
| Vulcan A | 2017 | Malmo Diet and Cancer Study cardiovascular cohort | Sweden | prospective cohort | Male | 16.7 years | 40 | 73 | FPG | Incidence | Administrative medical records or disease registries; ; | 71 | 1992 | NA | NA |
|  |  |  |  |  | Female | 16.7 years | 40 | 73 | FPG | Incidence | Administrative medical records or disease registries; ; | 74 | 2981 | NA | NA |
| Nilsen TL | 2001 | Nord-Trondelag cohort | Norway | prospective cohort | Male | 12 years | 20 | 101 | FPG | Incidence & Mortality | Administrative medical records or disease registries; Death certificates; | 362 | 36975 | NA | NA |
|  |  |  |  |  | Female | 12 years | 20 | 101 | FPG |  |  | 368 | 38244 | NA | NA |
| Chung YW | 2006 |  | Republic of Korea | case-control | Both | NI | 35 | 75 | FPG | Incidence | Administrative medical records or disease registries; ; | 78 | 135 | 44 | 57 |
| Yamada K | 1998 |  | Japan | case-control | Both | NI | 34 | 73 | FPG | Incidence | Administrative medical records or disease registries; ; | 77 | 232 | 43 | 155 |
| Lund Nilsen TI, | 2002 | HUNT study | Norway | prospective cohort | Male | 12 years | 20 | 99 | FPG | Incidence & Mortality | Administrative medical records or disease registries; ; | 362 | 36975 | NA | NA |
|  |  |  |  |  | Female | 12 years | 20 | 99 | FPG |  |  | 368 | 38244 | NA | NA |
| Shin A | 2011 | Korean National Health System (KNHS) study | Republic of Korea | prospective cohort | Male | 7 years | 30 | 80 | FPG | Incidence | Administrative medical records or disease registries; ; | 3051 | 869725 | NA | NA |
|  |  |  |  |  | Female | 7 years | 30 | 80 | FPG | Incidence | Administrative medical records or disease registries; ; | 1093 | 395501 | NA | NA |
| Pan XF | 2018 | China Kadoorie Biobank Study | China | prospective cohort | Both | 7.1 years | 30 | 79 | FPG | Incidence & Mortality | Administrative medical records or disease registries; Death certificates; | 1787 | 479057 | NA | NA |
| Jung KJ | 2016 | Korean Cancer Prevention Study II | Republic of Korea | case-cohort | Both | 5.3 years | 25 | 99 | FPG | Incidence & Mortality | Administrative medical records or disease registries; Death certificates; | 317 | 1691 | NA | NA |
| Will JC | 1998 | First Cancer Prevention Study | United States of America | prospective cohort | Male | 13 years | 30 | 99 | Diabetes | Incidence | Administrative medical records or disease registries; ; | 2722 | 302625 | NA | NA |
|  |  |  |  |  | Female | 13 years | 30 | 99 | Diabetes | Incidence |  | 2819 | 375796 | NA | NA |
| Yang YX | 2005 |  | United Kingdom | case-control | Both | 0.5 year | 18 | 99 | Diabetes | Incidence | Administrative medical records or disease registries; ; | 10447 | 114876 | 10447 | 104429 |
| Coughlin SS | 2004 | Cancer Prevention Study II | United States of America | prospective cohort | Both | 16 years | 30 | 99 | Diabetes | Mortality | Administrative medical records or disease registries; Death certificates; | 3681 | 1056243 | NA | NA |
| Swerdlow AJ | 2005 | Diabetes UK cohort | United Kingdom | prospective cohort | Both | NI | 30 | 49 | Diabetes | Mortality | Administrative medical records or disease registries; ; | 29 | 5066 | NA | NA |
|  |  |  |  |  |  |  |  |  | Diabetes | Incidence | Administrative medical records or disease registries; ; | 52 | 5066 | NA | NA |
| Kuriki K | 2007 |  | Japan | case-control | Male | NI | 40 | 80 | Diabetes | Incidence | Administrative medical records or disease registries; ; | 1524 | 15723 | 1524 | 14199 |
|  |  |  |  |  | Female | NI | 40 | 80 | Diabetes | Incidence | Administrative medical records or disease registries; ; | 1098 | 34667 | 549 | 33569 |
| Chodick G | 2010 |  | Israel | retrospective cohort | Both | 8 years | 21 | 99 | Diabetes | Incidence | Administrative medical records or disease registries; ; | 9584 | 100595 | NA | NA |
| Johnson JA | 2011 |  | Canada | retrospective cohort | Both | 4.35 years | 30 | 99 | Diabetes | Incidence | Administrative medical records or disease registries; Physician diagnosis; | 3117 | 370200 | NA | NA |
| Wotton CJ | 2011 | ORLS1 | United Kingdom | prospective cohort | Both | NI | 30 | 99 | Diabetes | Incidence & Mortality | Administrative medical records or disease registries; Death certificates; | 3976 | 291462 | NA | NA |
|  |  | ORLS2 |  |  |  |  |  |  |  |  |  | 864 | 192894 | NA | NA |
| Ogunleye AA | 2009 |  | Scotland | prospective cohort | Both | 3.9 years | 25 | 99 | Diabetes | Incidence & Mortality | Administrative medical records or disease registries; Death certificates; | 223 | 9577 | NA | NA |
| Atchison EA | 2011 |  | United States of America | prospective cohort | Male | 10.5 years | 18 | 100 | Diabetes | Incidence & Mortality | Administrative medical records or disease registries; ; | 34814 | 4,501,578 | NA | NA |
| Lo S-F | 2013 |  | Taiwan (Province of China) | prospective cohort | Both | 3.5 years | 25 | 99 | Diabetes | Incidence & Mortality | Administrative medical records or disease registries; ; | 17949 | 1790868 | NA | NA |
| Attner B | 2012 |  | Sweden | case-cohort | Both | 4 years | 45 | 84 | Diabetes | Incidence | Administrative medical records or disease registries; ; | 2394 | 167080 | NA | NA |
| Wideroff L | 1997 |  | Denmark | prospective cohort | Both | NI | 18 | 99 | Diabetes | Incidence | Administrative medical records or disease registries; ; | 1257 | 109581 | NA | NA |
| Verlato G | 2003 | Verona Diabetes study | Italy | prospective cohort | Male | 10 years | 25 | 99 | Diabetes | Mortality | Administrative medical records or disease registries; Death certificates; | 41 | 3366 | NA | NA |
|  | 2003 |  |  |  | Female | 10 years | 25 | 99 | Diabetes | Mortality | Administrative medical records or disease registries; Death certificates; | 24 | 3782 | NA | NA |
| Liu X | 2012 |  | Sweden | prospective cohort | Both | 7 years | 40 | 99 | Diabetes | Mortality | Administrative medical records or disease registries; ; | 752 | 1448 | NA | NA |
|  |  |  |  |  |  |  |  |  |  |  |  | 332 | 799 | NA | NA |
| Ulcickas Yood M | 2009 |  | United States of America | retrospective cohort | Both | 4 years | 18 | 99 | Diabetes | Incidence | Administrative medical records or disease registries; ; | 545 | 191223 | NA | NA |
| Steenland K | 1995 | NHANES I | United States of America | prospective cohort | Male | 7.7 years | 25 | 74 | Diabetes | Incidence & Mortality | Administrative medical records or disease registries; Death certificates; | 94 | 14407 | NA | NA |
|  |  |  |  |  | Female | 7.7 years | 25 | 74 | Diabetes | Incidence & Mortality | Administrative medical records or disease registries; Death certificates; | 82 | 14407 | NA | NA |
| Lee MY | 2012 |  | Taiwan (Province of China) | prospective cohort | Both | 11 years | 18 | 99 | Diabetes | Incidence | Administrative medical records or disease registries; ; | 9254 | 985815 | NA | NA |
| Qiang, J.K | 2020 |  | Canada | retrospective cohort | Both | 8 years | 18 | 99 | Diabetes | mortality | Cancer registries; ; | 14,785 | 44,178 | NA | NA |
| Kim SK | 2020 |  | Republic of Korea | retrospective cohort | Both | 8.6 years | 30 | 99 | Diabetes | Incidence | Administrative medical records or disease registries; ; | NI | 25,709,497 | NA | NA |
| Hidetaka I, et al | 2021 | JMDC Claims Database | Japan | prospective cohort | Both | 1137 days | 20 | 99 | FPG | Incidence | Administrative medical records or disease registries; ; | 5566 | 1441311 | NA | NA |
| Wu, Jingjing Mda | 2021 | Korean Multicenter Cancer Cohort | Republic of Korea | prospective cohort | Both | 11.3 years | 18 | 99 | FPG | Incidence | Administrative medical records or disease registries; ; | 132 | 11632 | NA | NA |
| Hsu, S.-H | 2022 | Nationwide Taiwanese Survey on Hypertension, Hyperglycemia, and Hyperlipidemia (TwSHHH) | Taiwan (Province of China) | prospective cohort | Both | 16.7 years | 30 | 80 | FPG | Incidence | Cancer registries; ; | 93 | 4764 | NA | NA |
| Corcoran NM | 2022 | UK Biobank cohort | United Kingdom | prospective cohort | Both | 7 years | 37 | 73 | Diabetes | Incidence | Cancer registries; ; | 3669 | 500222 | NA | NA |
|  |  |  |  |  |  | 11 years | 37 | 73 | Diabetes | mortality | Cancer registries; ; | 916 | 500222 | NA | NA |
| Yang Z | 2023 | The Yinzhou Health Information System | China | retrospective cohort | Both | 9.2 years | 18 | 99 | Diabetes | Incidence | Administrative medical records or disease registries; ; | NI | 208289 | NA | NA |
|  |  |  |  |  |  | 9.32 years | 18 | 99 | Diabetes | mortality | death certificates; ; | NI | 208289 | NA | NA |
| Wu, Po-Hsien Mda | 2023 | Taiwan National Health Insurance | Taiwan (Province of China) | retrospective cohort | Both | 8.55 years | 18 | 99 | Diabetes | Incidence | Medical records; ; | 160 | 264930 | NA | NA |
| Low EE | 2020 | No name - Veteranâ€™s Health Administration (VHA) data | United States of America | case-control | Both | NI | 18 | 49 | Diabetes | Incidence | Medical records; ; | 651 | 68067 | 651 | 67416 |
| Yilmaz Kavcar SR | 2022 | Balcova Heart Study (BHS) | Turkey | prospective cohort | Male | 6 years | 30 | 99 | FPG | Incidence | Cancer registries; ; | 19 | 15869 | NA | NA |
|  |  |  |  |  | Female | 6 years | 30 | 99 | FPG | Incidence | Cancer registries; ; | 20 | 15869 | NA | NA |
| Pradhan R | 2022 | Clinical Practice Research Datalink (CPRD) | United Kingdom | prospective cohort | Both | 8.4 years | 18 | 99 | Diabetes | Incidence | National database; ; | 17482 | 1780428 | NA | NA |
| Vicentini M | 2022 | Diabetes Registry and Population-based Cancer Registry | Italy | prospective cohort | Both | NI | 20 | 84 | Diabetes | Incidence | Cancer registries; ; | 1476 | 401051 | NA | NA |
| Yoo, T.K | 2022 | Kangbuk Samsung Health Study | Republic of Korea | prospective cohort | Both | 6.99 years | 18 | 99 | HbA1C | Mortality | Death certificate; ; | 118 | 550931 | NA | NA |
| Gurney J | 2022 | Statistics New Zealand's Integrated Data Infrastructure (IDI) | New Zealand | prospective cohort | Both | NI | 18 | 99 | Diabetes | Incidence | Cancer registries; ; | 28227 | NI | NA | NA |
| Campbell PT | 2022 | Cancer Prevention Study-II (CPS-II) Nutrition Cohort | United States of America | case-control | Both | 10.4 years | 18 | 99 | HbA1C | Incidence | Cancer registries; medical records; | 145 | 904 | 90 | 759 |
| Goto A | 2020 | Japan Public Health Center-based Prospective (JPHC) Study. | Japan | case-control | Both | 15.9 years | 40 | 69 | FPG | Incidence | Cancer registries; death certificates; | 664 | 10,536 | NA | NA |
| Hu Y | 2021 | Health Professionals Follow-up Study | United States of America | prospective cohort | Male | NI | 40 | 75 | FPG | Incidence | Medical records; ; | 1139 | 45604 | NA | NA |
|  |  | Nursesâ€™ Health Study (NHS) |  |  | Female | NI | 30 | 55 | FPG | Incidence | Medical records; ; | 2474 | 113429 | NA | NA |
| Schumacher AJ | 2021 | Kaiser Permanente Southern California (KPSC) | United States of America | case-control | Both | NI | 15 | 49 | HbA1C | Incidence | Cancer registries; ; | 1032 | 6160 | 1032 | 5128 |
| Park SH | 2023 |  | New Zealand | prospective cohort | Both | NI | 18 | 99 | Diabetes | Incidence | Cancer registries; ; | 23148 | 4236350 | NA | NA |
| Herold, M | 2023 |  | Hungary | retrospective cohort | Both | NI | 18 | 99 | Diabetes | mortality | Medical records; ; | NI | 817 | NA | NA |
| Kim, J | 2020 | Korean National Cancer Center Community (KNCCC) Cohort | Republic of Korea | prospective cohort | Both | NI | 30 | 99 | FPG | Incidence | Cancer registries; ; | 111 | 6985 | NA | NA |
| Rothwell JA | 2022 | UK Biobank cohort | United Kingdom | prospective cohort | Both | 7.1 years | 18 | 99 | HbA1C | Incidence | Cancer registries; death certificates; | 478 | 15152 | NA | NA |
| Chang VCs | 2021 | Ontario Cancer Registry (OCR) | Canada | case-control | Both | NI | 20 | 49 | Diabetes | Incidence | Cancer registries; ; | 175 | 428 | 175 | 253 |
| Mikaeel RR | 2021 | The South Australian Young Onset Colorectal Polyp and Cancer Study (SAYO) | Australia | case-control | Both | NI | 18 | 54 | Diabetes | Incidence | Medical records; ; | 90 | 330 | 90 | 240 |
| Bjornsdottir HH | 2020 | The Swedish National Diabetes Register (NDR) | Sweden | case-control | Both | 6.6 years | 18 | 99 | Diabetes | Incidence | Death registries; ; | NI | 2744838 | NA | NA |
|  |  |  |  |  |  |  |  |  |  | mortality | Death registries; ; | NI | 2744838 | NA | NA |
| Kim DB | 2021 | Korean National Health Insurance System | Republic of Korea | prospective cohort | Both | 5.4 years | 20 | 99 | FPG | Incidence | Cancer registries; ; | 120 579 | 23121360 | NA | NA |
| Boustany A | 2023 | Explorys Inc., Cleveland, OH, USA database | United States of America | case-control | Both | 20 years | 18 | 65 | Diabetes | Incidence | Medical records; ; | 82420 | 47714750 | 82420 | 47632330 |
| Joseph DF | 2021 |  | United States of America | Retrospective cohort | Both | NI | 40 | 75 | Diabetes | Incidence | Endoscopic exam; ; | 7 | 2599 |  |  |

3.3 Breast Cancer

| Author | Year of publication | Study Name | Location | Study Design | Sex | Follow-up | Age Start | Age End | Exposure Measure | Endpoint | Disease Ascertainment | Events | Sample Size | Cases | Controls |
| --- | --- | --- | --- | --- | --- | --- | --- | --- | --- | --- | --- | --- | --- | --- | --- |
| Jee SH | 2005 | Korean Cancer Prevention Study | Republic of Korea | prospective cohort | Female | 10 years | 30 | 95 | FPG | Mortality | Administrative medical records or disease registries; ; | NI | 468615 | NA | NA |
|  | 2005 | Korean Cancer Prevention Study | Republic of Korea | prospective cohort | Female | 10 years | 30 | 95 | FPG | Incidence | Administrative medical records or disease registries; ; | NI | 468615 | NA | NA |
| Mink PJ | 2002 | Atherosclerosis Risk in Communities study (ARIC) | United States of America | prospective cohort | Female | 7.1 years | 45 | 64 | FPG | Incidence | Administrative medical records or disease registries; ; | 187 | 7894 | NA | NA |
| Haseen SD | 2015 | no name - Pakistan 2014 study | Pakistan | case-control | Female | NI | 25 | 99 | FPG | Incidence | Administrative medical records or disease registries; ; | 175 | 350 | 175 | 175 |
| Melvin JC | 2017 | AMORIS study | Stockholm | prospective cohort | Female | 11.6 years | 20 | 99 | FPG | Incidence | Administrative medical records or disease registries; ; | 1824 | 812,073 | NA | NA |
| Kabat GC | 2009 | Women's Health Initiative | United States of America | prospective cohort | Female | 8 years | 50 | 79 | FPG | Incidence | Administrative medical records or disease registries; ; | 190 | 5450 | NA | NA |
| Pan XF | 2018 | China Kadoorie Biobank Study | China | prospective cohort | Female | 7.1 years | 30 | 79 | FPG | Incidence & Mortality | Administrative medical records or disease registries; Death certificates; | 1472 | 508,892 | NA | NA |
| Kuriki K | 2007 | no name - Japan 2000 study | Japan | case-control | Female | NI | 40 | 80 | Diabetes | Incidence | Administrative medical records or disease registries; ; | 2517 | 36086 | 2517 | 33569 |
| Chodick G | 2010 | no name - Israel 2008 study | Israel | retrospective cohort | Male | 8 years | 21 | 99 | Diabetes | Incidence | Administrative medical records or disease registries; ; | 14 | 100595 | NA | NA |
|  |  |  |  |  | Female | 9 years | 21 | 99 | Diabetes | Incidence | Administrative medical records or disease registries; ; | 1167 |  | NA | NA |
| Johnson JA | 2011 | no name - Canada 2006 cohort study | Canada | retrospective cohort | Female | 4.35 years | 30 | 99 | Diabetes | Incidence | Administrative medical records or disease registries; Physician diagnosis; | 2381 | 169012 | NA | NA |
| Wotton CJ. | 2011 | ORLS1 | United Kingdom | prospective cohort | Female | NI | 30 | 99 | Diabetes | Incidence & Mortality | Administrative medical records or disease registries; Death certificates; | 2412 | 132271 | NA | NA |
|  |  | ORLS2 |  |  |  |  | 30 | 99 | Diabetes | Incidence & Mortality |  | 1208 | 90427 | NA | NA |
| Ogunleye AA | 2009 | no name - United Kingdom 2004 study | Scotland | prospective cohort | Both | 3.9 years | 25 | 99 | Diabetes | Incidence & Mortality | Administrative medical records or disease registries; Death certificates; | 155 | 9577 | NA | NA |
| Lo S-F | 2013 | no name - Taiwan 2009 another study | Taiwan (Province of China) | prospective cohort | Female | 3.5 years | 25 | 99 | Diabetes | Incidence & Mortality | Administrative medical records or disease registries; ; | 7424 | 913343 | NA | NA |
| Attner B | 2012 | no name - Sweden 2007 study | Sweden | case-cohort | Female | 4 years | 45 | 84 | Diabetes | Incidence | Administrative medical records or disease registries; ; | 2724 | 167080 | NA | NA |
| Swerdlow AJ | 2005 | Diabetes UK cohort | United Kingdom | prospective cohort | Female | NI | 30 | 49 | Diabetes | Mortality | Administrative medical records or disease registries; ; | 17 | 2122 | NA | NA |
|  |  |  |  |  |  |  |  |  |  | Incidence | Administrative medical records or disease registries; ; | 41 | 2122 | NA | NA |
| Coughlin SS | 2004 | Cancer Prevention Study II | United States of America | prospective cohort | Female | 16 years | 30 | 99 | Diabetes | Mortality | Administrative medical records or disease registries; Death certificates; | 4346 | 588321 | NA | NA |
| Wideroff L | 1997 | no name - Denmark 1969 study | Denmark | prospective cohort | Female | NI | 18 | 99 | Diabetes | Incidence | Administrative medical records or disease registries; ; | 777 | 55010 | NA | NA |
| Verlato G | 2003 | Verona Diabetes study | Italy | prospective cohort | Female | 10 years | 25 | 99 | Diabetes | Mortality | Administrative medical records or disease registries; Death certificates; | 57 | 3782 | NA | NA |
| Liu X | 2012 | no name - Sweden 2008 study | Sweden | prospective cohort | Female | 7 years | 40 | 99 | Diabetes | Mortality | Administrative medical records or disease registries; ; | 475 | 1495 | NA | NA |
| Lipscombe LL | 2006 | no name - Canada 2002 study | Canada | retrospective cohort | Female | 4.5 years | 55 | 79 | Diabetes | Incidence | Administrative medical records or disease registries; Death certificates; | 1655 | 494340 | NA | NA |
| Baron JA | 2001 | no name - United States 1994 study | United States of America | case-control | Female | NI | 50 | 75 | Diabetes | Incidence | Administrative medical records or disease registries; ; | 422 | 890 | 422 | 468 |
| Weiss HA | 1999 | no name - United States 1992 study | United States of America | case-control | Female | NI | 20 | 54 | Diabetes | Incidence | Administrative medical records or disease registries; ; | 33 | 64 | 33 | 31 |
| Goodman MT | 197 | Life Span study | Japan | prospective cohort | Female | 8.31 years | 18 | 99 | Diabetes | Incidence | Administrative medical records or disease registries; ; | 161 | 22200 | NA | NA |
| Steenland K | 1995 | NHANES I | United States of America | prospective cohort | Female | 7.7 years | 25 | 74 | Diabetes | Incidence & Mortality | Administrative medical records or disease registries; Death certificates; |  | 14407 | NA | NA |
| Sanderson M. | 2010 | South Texas Women's Health project | Texas | case-control | Female | NI | 30 | 79 | Diabetes | Incidence | Administrative medical records or disease registries; ; | 65 | 383 | 65 | 318 |
| Wu AH | 2007 | no name - United States 2001 other study | California | case-control | Female | NI | 25 | 74 | Diabetes | Incidence | Administrative medical records or disease registries; ; | 1248 | 2396 | 1248 | 1148 |
| Crispo A | 2017 | no name - Italy 2013 study | Italy | case-control | Female | NI | 25 | 99 | Diabetes | Incidence | Administrative medical records or disease registries; ; | 557 | 1149 | 557 | 592 |
| Bosco JL | 2012 | Black Womens Health Study | United States of America | prospective cohort | Female | 10.5 years | 21 | 69 | Diabetes | Incidence | Administrative medical records or disease registries; ; | 1228 | 49172 | NA | NA |
| Ronco AL | 2012 | no name - Uruguay 2010 study | Uruguay | case-control | Female | NI | 18 | 99 | Diabetes | Incidence | Administrative medical records or disease registries; ; | 253 | 750 | 253 | 497 |
| Garmendia ML | 2007 | no name - Chile 2005 study | Chile | case-control | Female | NI | 33 | 86 | Diabetes | Incidence | Administrative medical records or disease registries; ; | 170 | 340 | 170 | 170 |
|  | 2007 | no name - Chile 2005 study | Chile | case-control | Female | NI | 33 | 86 | Diabetes | Incidence | Administrative medical records or disease registries; ; | 122 | 238 | 122 | 116 |
| Tseng CH | 2009 | no name - Taiwan 2006 other study | Taiwan (Province of China) | prospective cohort | Female | 12 years | 25 | 99 | Diabetes | Mortality | Administrative medical records or disease registries; ; | 14230 | 131573 | NA | NA |
| Lee MY | 2009 | no name - Taiwan 2009 other study | Taiwan (Province of China) | prospective cohort | Female | 11 years | 18 | 99 | Diabetes | Incidence | Administrative medical records or disease registries; ; | 3911 | 497037 | NA | NA |
| Kim SK | 2007 | No name - Korea study 2007 | Republic of Korea | retrospective cohort | Female | 8.6 years | 30 | 99 | Diabetes | Incidence | Administrative medical records or disease registries; ; | NI | 25,709,497 | NA | NA |
| Lee, S.H | 2020 | Korean National Health Insurance Service (NHIS) - Health Examination Cohort | Republic of Korea | prospective cohort | Female | 10 years | 40 | 79 | FPG | Incidence | Medical records; ; | NI | 165,886 | NA | NA |
| Zimbalist, A.S | 2022 | Kaiser Permanente Northern California (KPNC) | United States of America | retrospective cohort | Female | 8.6 years | 18 | 99 | FPG | mortality | Medical records; ; | 1,080 | 13,434 | NA | NA |
| Zhang F | 2023 | No name - Germany study | Germany | prospective cohort | Female | 4.45 years | 30 | 80 | HbA1C | Incidence | Medical records; ; | 183 | 10183 | NA | NA |
| Yang Z | 2023 | The Yinzhou Health Information System | China | retrospective cohort | Female | 9.2 years | 18 | 99 | Diabetes | Incidence | Administrative medical records or disease registries; ; | 951 | 208289 | NA | NA |
| Yilmaz Kavcar SR | 2022 | Balcova Heart Study (BHS) | TÃ¼rkiye | prospective cohort | Female | 6 years | 30 | 99 | FPG | Incidence | Cancer registries; ; | 84 | 15869 | NA | NA |
| Pradhan R | 2022 | Clinical Practice Research Datalink (CPRD) | United Kingdom | prospective cohort | Both | 8.4 years | 18 | 99 | Diabetes | Incidence | National database; ; | NI | 1780428 | NA | NA |
| Vicentini M | 2022 | Diabetes Registry and Population-based Cancer Registry | Italy | prospective cohort | Both | NI | 20 | 84 | Diabetes | Incidence | Cancer registries; ; | NI | 401051 | NA | NA |
| Yoo, T.K | 2022 | Kangbuk Samsung Health Study | Republic of Korea | prospective cohort | Both | 7 years | 18 | 99 | HbA1C | Mortality | Death certificate; ; | 70 | 550931 | NA | NA |
| Gurney J | 2022 | Statistics New Zealandâ€™s Integrated Data Infrastructure (IDI) | New Zealand | prospective cohort | Both | NI | 18 | 99 | Diabetes | Incidence | Cancer registries; ; | 28167 | NI | NA | NA |
| Campbell PT | 2022 | Cancer Prevention Study-II (CPS-II) Nutrition Cohort | United States of America | case-control | Both | 10.4 years | 18 | 99 | HbA1C | Incidence | Cancer registries; medical records; | 190 | 554 | 190 | 364 |
| Tran TXM | 2023 | Korean National Health Insurance System | Republic of Korea | prospective cohort | Female | 10 years | 40 | 75 | FPG | mortality | National database; ; |  | 8309393 | NA | NA |
| Goto A | 2020 | Japan Public Health Center-based Prospective (JPHC) Study. | Japan | case-control | Female | 15.9 years | 40 | 69 | FPG | Incidence | Cancer registries; death certificates; | 239 | 6837 | NA | NA |
| Hu Y | 2021 | Nurses Health Study (NHS) | United States of America | prospective cohort | Female | NI | 30 | 55 | FPG | Incidence | Medical records; ; | 13077 | 113429 | NA | NA |
| Hossain FM | 2022 | No name - Louisiana study | United States of America | case-control | Female | NI | 20 | 99 | Diabetes | Incidence | Cancer registries; ; | 2295 | 11074 | 2295 | 8779 |
| Park SH | 2023 | No name - New Zealand study | New Zealand | prospective cohort | Female | NI | 18 | 99 | Diabetes | Incidence | Cancer registries; ; | 27312 | 4236350 | NA | NA |
| Bjornsdottir HH | 2020 | The Swedish National Diabetes Register (NDR) | Sweden | case-control | Female | 6.6 years | 18 | 99 | Diabetes | Incidence | Death registries; ; | NI | 2744838 | NA | NA |
|  | 2020 | The Swedish National Diabetes Register (NDR) | Sweden | case-control | Female | 6.6 years | 18 | 99 | Diabetes | mortality | Death registries; ; | NI | 2744838 | NA | NA |
| Peila R | 2020 | UK Biobank cohort | United Kingdom | prospective cohort | Female | 7.1 years | 40 | 69 | HbA1C | Incidence | Medical records; National Database; | 5917 | 476517 | NA | NA |

3.5 Liver Cancer

| Author | Year of publication | Study Name | Location | Study Design | Sex | Follow-up | Age Start | Age End | Exposure Measure | Endpoint | Disease Ascertainment | Events | Sample Size | Cases | Controls |
| --- | --- | --- | --- | --- | --- | --- | --- | --- | --- | --- | --- | --- | --- | --- | --- |
| Jee SH | 2005 | Korean Cancer Prevention Study | Republic of Korea | prospective cohort | Male | 10 years | 30 | 95 | FPG | Mortality | Administrative medical records or disease registries; ; | NI | 829770 | NA | NA |
|  | 2005 | Korean Cancer Prevention Study | Republic of Korea | prospective cohort | Female | 10 years | 30 | 95 | FPG | Mortality | Administrative medical records or disease registries; ; | NI | 468615 | NA | NA |
|  | 2005 | Korean Cancer Prevention Study | Republic of Korea | prospective cohort | Male | 10 years | 30 | 95 | FPG | Incidence | Administrative medical records or disease registries; ; | NI | 829770 | NA | NA |
|  | 2005 | Korean Cancer Prevention Study | Republic of Korea | prospective cohort | Female | 10 years | 30 | 95 | FPG | Incidence | Administrative medical records or disease registries; ; | NI | 468615 | NA | NA |
| Chao LT | 2011 |  | Taiwan (Province of China) | case-control | Male | NI | 30 | 65 | FPG | Incidence | Administrative medical records or disease registries; ; | 124 | 1142 | 124 | 1018 |
| Loftfield E | 2016 | Alpha-Tocopherol Beta-Carotene study | Finland | nested case-control | Male | 22 years | 50 | 69 | FPG | Incidence | Administrative medical records or disease registries; ; | 138 | 391 | 138 | 253 |
| Gupta SP | 2013 |  | Nepal | case-control | Both | NI | 30 | 99 | FPG | Incidence | Administrative medical records or disease registries; ; | 4 | 30 | 4 | 26 |
| Feng X | 2017 | Kailuan cohort | Hebei | prospective cohort | Male | 6.89 years | 19 | 99 | FPG | Incidence | Administrative medical records or disease registries; Death certificates; | 267 | 109 169 | NA | NA |
| Kim K | 2018 | National Health Insurance Service (NHIS) cohort | Republic of Korea | prospective cohort | Male | 8 years | 19 | 99 | FPG | Incidence | Administrative medical records or disease registries; ; | 11241 | 214167 | NA | NA |
| Pan XF | 2018 | China Kadoorie Biobank Study | China | prospective cohort | Both | 7.1 years | 30 | 79 | FPG | Incidence & Mortality | Administrative medical records or disease registries; Death certificates; | 1797 | 479057 | NA | NA |
| Kuriki K | 2007 |  | Japan | case-control | Male | NI | 40 | 80 | Diabetes | Incidence | Administrative medical records or disease registries; ; | 313 | 14512 | 313 | 14199 |
|  | 2007 |  | Japan | case-control | Female | NI | 40 | 80 | Diabetes | Incidence | Administrative medical records or disease registries; ; | 83 | 33652 | 83 | 33569 |
| Chodick G | 2010 |  | Israel | retrospective cohort | Male | 8 years | 21 | 99 | Diabetes | Incidence | Administrative medical records or disease registries; ; | 46 | 52913 | NA | NA |
|  | 2010 |  | Israel | retrospective cohort | Female | 8 years | 21 | 99 | Diabetes | Incidence | Administrative medical records or disease registries; ; | 27 | 47682 | NA | NA |
| Wotton CJ | 2011 | ORLS1 | United Kingdom | prospective cohort | Both | NI | 30 | 99 | Diabetes | Incidence & Mortality | Administrative medical records or disease registries; Death certificates; | 309 | 291462 | NA | NA |
|  |  | ORLS2 |  |  |  |  | 30 | 99 |  |  |  | 116 | 192894 | NA | NA |
| Fujino Yoshihisa | 2001 |  | Japan | prospective cohort | Male | 11.5 years | 40 | 79 | Diabetes | Mortality | Administrative medical records or disease registries; ; | 82 | 7308 | NA | NA |
| El-serag HB | 2004 |  | United States of America | prospective cohort | Both | 10 years | 20 | 99 | Diabetes | Incidence & Mortality | Administrative medical records or disease registries; ; | 7799 | 824263 | NA | NA |
| Lai M-S | 2006 | Keelung Community-Based Integrated Screening (KCIS) study | Taiwan (Province of China) | prospective cohort | Both | 2.78 years | 30 | 99 | Diabetes | Incidence | Imaging/scan; | 85 | 48664 | NA | NA |
| Torisu Yuichi | 2007 |  | Japan | retrospective cohort | Male | 6.8 years | 34 | 80 | Diabetes | Incidence | Imaging/scan; ; | 9 | 47 | NA | NA |
| Ioannou GN | 2007 |  | United States of America | retrospective cohort | Both | 3.6 years | 18 | 99 | Diabetes | Incidence | Administrative medical records or disease registries; ; | 100 | 2126 | NA | NA |
| Costanzo GGD | 2008 |  | Italy | prospective cohort | Both | 7 years | 42 | 82 | Diabetes | Incidence | Physician diagnosis; ; | 63 | 138 | NA | NA |
| Swerdlow AJ | 2005 | Diabetes UK cohort | United Kingdom | prospective cohort | Both | NI | 30 | 49 | Diabetes | Mortality | Administrative medical records or disease registries; ; | 11 | 5066 | NA | NA |
| Coughlin SS | 2004 | Cancer Prevention Study II | United States of America | prospective cohort | Male | 16 years | 30 | 99 | Diabetes | Mortality | Administrative medical records or disease registries; Death certificates; | 751 | 467922 | NA | NA |
|  | 2004 | Cancer Prevention Study II | United States of America | prospective cohort | Female | 16 years | 30 | 99 | Diabetes | Mortality | Administrative medical records or disease registries; Death certificates; | 418 | 588321 | NA | NA |
| Ogunleye AA | 2009 |  | Scotland | prospective cohort | Both | 3.9 years | 25 | 99 | Diabetes | Incidence & Mortality | Administrative medical records or disease registries; Death certificates; | 2025 | 28731 | NA | NA |
| Atchison EA | 2011 |  | United States of America | prospective cohort | Male | 10.5 years | 18 | 100 | Diabetes | Incidence & Mortality | Administrative medical records or disease registries; ; | 5160 | 371810 | NA | NA |
| Lo S-F | 2013 |  | Taiwan (Province of China) | prospective cohort | Both | 3.5 years | 25 | 99 | Diabetes | Incidence & Mortality | Administrative medical records or disease registries; ; | 19207 | 1790868 | NA | NA |
| Attner B | 2012 |  | Sweden | case-cohort | Both | 4 years | 45 | 84 | Diabetes | Incidence | Administrative medical records or disease registries; ; | 283 | 167080 | NA | NA |
| Wideroff L | 1997 |  | Denmark | prospective cohort | Both | NI | 18 | 99 | Diabetes | Incidence | Administrative medical records or disease registries; ; | 258 | 109581 | NA | NA |
| Verlato G | 2003 | Verona Diabetes study | Italy | prospective cohort | Male | 10 years | 25 | 99 | Diabetes | Mortality | Administrative medical records or disease registries; Death certificates; | 40 | 3366 | NA | NA |
|  | 2003 | Verona Diabetes study | Italy | prospective cohort | Female | 10 years | 25 | 99 | Diabetes | Mortality | Administrative medical records or disease registries; Death certificates; | 24 | 3782 | NA | NA |
| Liu X, | 2012 |  | Sweden | prospective cohort | Both | 7 years | 40 | 99 | Diabetes | Mortality | Administrative medical records or disease registries; ; | 744 | 987 | NA | NA |
| Ulcickas Yood M | 2009 |  | United States of America | retrospective cohort | Both | 4 years | 18 | 99 | Diabetes | Incidence | Administrative medical records or disease registries; ; | 61 | 191223 | NA | NA |
| Lee MY | 2012 |  | Taiwan (Province of China) | prospective cohort | Both | 11 years | 18 | 99 | Diabetes | Incidence | Administrative medical records or disease registries; ; | 5964 | 985815 | NA | NA |
| Kim SK | 2020 |  | Republic of Korea | retrospective cohort | Both | 8.6 years | 30 | 99 | Diabetes | Incidence | Administrative medical records or disease registries; ; | NI | 25,709,497 | NA | NA |
| Chen CL | 2020 | KeelungCommunity-Based Integrated Screening program | Taiwan (Province of China) | prospective cohort | Female | 4.47 years | 30 | 99 | FPG | Incidence | Physician diagnosis; Imaging/scan; | 99 | 74782 | NA | NA |
|  | 2020 | KeelungCommunity-Based Integrated Screening program | Taiwan (Province of China) | prospective cohort | Male | 4.47 years | 30 | 99 | FPG | Incidence | Physician diagnosis; Imaging/scan; | 99 | 74782 | NA | NA |
| Ma X | 2021 | The Kailuan study | China | prospective cohort | Both | 11.47 years | 18 | 99 | FPG | Incidence | Medical records; ; | 388 | 98936 | NA | NA |
| Yoo JJ | 2021 | Korean National Health Insurance System | Republic of Korea | prospective cohort | Both | 6.75 years | 40 | 99 | FPG | Incidence | Administrative medical records or disease registries; ; | 5494 | 674178 | NA | NA |
| Xia, B | 2021 | UK Biobank cohort | United Kingdom | prospective cohort | Male | 6.6 years | 40 | 69 | FPG | Incidence | Cancer registries; Death registries; | 175 | 219383 | NA | NA |
|  | 2021 | UK Biobank cohort | United Kingdom | prospective cohort | Female | 6.6 years | 40 | 69 | FPG | Incidence | Cancer registries; Death registries; | 101 | 256276 | NA | NA |
| Zhang L | 2023 | Jinchang Cohort | China | nested case-control | Both | NI | 18 | 99 | FPG | Incidence | Medical records; ; | 162 | 810 | 162 | 648 |
| Yang Z | 2023 | The Yinzhou Health Information System | China | retrospective cohort | Both | 9.2 years | 18 | 99 | Diabetes | Incidence | Administrative medical records or disease registries; ; | NI | 208289 | NA | NA |
|  |  |  |  |  | Both | 9.32 years | 18 | 99 | Diabetes | mortality | death certificates; ; | NI | 208289 | NA | NA |
| HernÃ¡ndez-GarduÃ±o E | 2021 |  | Mexico | prospective cohort | Both | NI | 20 | 99 | Diabetes | mortality | death certificates; ; | 27033 | 710292 | NA | NA |
| Yilmaz Kavcar SR | 2022 | Balcova Heart Study (BHS) | TÃ¼rkiye | prospective cohort | Male | 6 years | 30 | 99 | FPG | Incidence | Cancer registries; ; | 6 | 15869 | NA | NA |
| Hemminki K | 2023 | Swedish Cancer Registry | Sweden | prospective cohort | Both | NI | 18 | 99 | Diabetes | Incidence | Cancer registries; ; | 8674 | 13 567 134 | NA | NA |
| Vicentini M | 2022 | Diabetes Registry and Population-based Cancer Registry | Italy | prospective cohort | Both | NI | 20 | 84 | Diabetes | Incidence | Cancer registries; ; | 411 | 401051 | NA | NA |
| Yoo, T.K | 2022 | Kangbuk Samsung Health Study | Republic of Korea | prospective cohort | Both | 6.99 years | 18 | 99 | HbA1C | Mortality | Death certificate; ; | 215 | 550931 | NA | NA |
| Gurney j | 2022 | Statistics New Zealand's Integrated Data Infrastructure (IDI) | New Zealand | prospective cohort | Both | NI | 18 | 99 | Diabetes | Incidence | Cancer registries; ; | 2859 | NI | NA | NA |
| Campbell PT | 2022 | Cancer Prevention Study-II (CPS-II) Nutrition Cohort | United States of America | case-control | Both | 10.4 years | 18 | 99 | HbA1C | Incidence | Cancer registries; medical records; | 14 | 774 | 7 | 533 |
| Tran TXM | 2023 | Korean National Health Insurance System | Republic of Korea | prospective cohort | Female | 10 years | 40 | 75 | FPG | mortality | National database; ; | 18782 | 8309393 | NA | NA |
| Conway RBN | 2021 | The Southern Community Cohort Study (SCCS) | United States of America | prospective cohort | Both | 10.5 years | 40 | 79 | Diabetes | Incidence | Cancer registries; ; | 320 | 74956 | NA | NA |
| Goto A | 2020 | Japan Public Health Center-based Prospective (JPHC) Study. | Japan | case-control | Both | 15.9 years | 40 | 69 | FPG | Incidence | Cancer registries; death certificates; | 168 | 10536 | NA | NA |
| Hu Y | 2021 | Health Professionals Follow-up Study | United States of America | prospective cohort | Male | NI | 40 | 75 | FPG | Incidence | Medical records; ; | 55 | 45604 | NA | NA |
| Hu Y | 2021 | Nurses Health Study (NHS) | United States of America | prospective cohort | Female | NI | 30 | 55 | FPG | Incidence | Medical records; ; | 114 | 113429 | NA | NA |
| Park SH | 2023 |  | New Zealand | prospective cohort | Both | NI | 18 | 99 | Diabetes | Incidence | Cancer registries; ; | 2727 | 4236350 | NA | NA |
| Hatia RI | 2023 |  | United States of America | case-control | Both | NI | 18 | 99 | Diabetes | Incidence | Imaging; ; | 429 | 1490 | 429 | 1061 |
| Kim K | 20233 | Korean National Health Insurance System | Republic of Korea | prospective cohort | Both | 9.3 years | 65 | 99 | FPG | Incidence | Medical records; ; | 16384 | 1032880 | NA | NA |
| Rothwell JA | 2022 | UK Biobank cohort | United Kingdom | prospective cohort | Both | 7.1 years | 18 | 99 | HbA1C | Incidence | Cancer registries; death certificates; | 220 | 15152 | NA | NA |
| Bjornsdottir HH | 2020 | The Swedish National Diabetes Register (NDR) | Sweden | prospective cohort | Both | 6.6 years | 18 | 99 | Diabetes | Incidence | Death registries; ; | NI | 23334838 | NA | NA |
|  |  |  |  |  |  |  |  |  |  | Mortality | Death registries; ; | NI | 23334838 | NA | NA |

3.6 Pancreatic Cancer

| Author | Year of publication | Study Name | Location | Study Design | Sex | Follow-up | Age Start | Age End | Exposure Measure | Endpoint | Disease Ascertainment | Events | Sample Size | Cases | Controls |
| --- | --- | --- | --- | --- | --- | --- | --- | --- | --- | --- | --- | --- | --- | --- | --- |
| Jee SH | 2005 | Korean Cancer Prevention Study | Republic of Korea | prospective cohort | Male | 10 years | 30 | 95 | FPG | Mortality | Administrative medical records or disease registries; ; | NI | 829770 | NA | NA |
|  |  | Korean Cancer Prevention Study | Republic of Korea | prospective cohort | Female | 10 years | 30 | 95 | FPG | Mortality | Administrative medical records or disease registries; ; | NI | 468615 | NA | NA |
|  |  | Korean Cancer Prevention Study | Republic of Korea | prospective cohort | Male | 10 years | 30 | 95 | FPG | Incidence | Administrative medical records or disease registries; ; | NI | 829770 | NA | NA |
|  |  | Korean Cancer Prevention Study | Republic of Korea | prospective cohort | Female | 10 years | 30 | 95 | FPG | Incidence | Administrative medical records or disease registries; ; | NI | 468615 | NA | NA |
| Stolzenberg-Solomon RZ | 2005 | ATBC study cohort | Finland | nested case-control | Male | 13.8 years | 50 | 69 | FPG | Incidence | Administrative medical records or disease registries; ; | 233 | 624 | 233 | 391 |
| Wu Q | 2012 |  | Beijing | case-control | Both | NI | 25 | 99 | FPG | Incidence | Administrative medical records or disease registries; ; | 98 | 188 | 98 | 90 |
| Yun JE | 2006 | National Health Insurance Corporation (NHIC) cohort | Republic of Korea | prospective cohort | Male | 10 years | 40 | 65 | FPG | Incidence | Administrative medical records or disease registries; ; | 863 | 446407 | NA | NA |
|  |  |  |  |  |  |  |  |  |  | Mortality | Death certificates; ; | 816 |  | NA | NA |
| Kuriki K | 2007 |  | Japan | case-control | Male | NI | 40 | 80 | Diabetes | Incidence | Administrative medical records or disease registries; ; | 120 | 14319 | 120 | 14199 |
|  |  |  |  |  | Female |  |  |  |  |  |  | 71 | 33640 | 71 | 33569 |
| Chodick G | 2010 |  | Israel | retrospective cohort | Male | 8 years | 21 | 99 | Diabetes | Incidence | Administrative medical records or disease registries; ; | 114 | 52913 | NA | NA |
|  |  |  |  |  | Female |  |  |  |  |  |  | 103 | 47682 | NA | NA |
| Wotton CJ | 2011 | ORLS1 | United Kingdom | prospective cohort | Both | NI | 30 | 99 | Diabetes | Incidence & Mortality | Administrative medical records or disease registries; Death certificates; | 853 | 291462 | NA | NA |
|  | 2011 | ORLS2 |  |  |  |  |  |  |  |  |  | 367 | 192894 | NA | NA |
| Ogunleye AA | 2009 |  | Scotland | prospective cohort | Both | 3.9 years | 25 | 99 | Diabetes | Incidence & Mortality | Administrative medical records or disease registries; Death certificates; | 51 | 28731 | NA | NA |
| Atchison EA | 2011 |  | United States of America | prospective cohort | Male | 10.5 years | 18 | 100 | Diabetes | Incidence & Mortality | Administrative medical records or disease registries; ; | 7639 | 371810 | NA | NA |
| Lo S-F | 2013 |  | Taiwan (Province of China) | prospective cohort | Both | 3.5 years | 25 | 99 | Diabetes | Incidence & Mortality | Administrative medical records or disease registries; ; | 2352 | 1790868 | NA | NA |
| Attner B | 2012 |  | Sweden | case-cohort | Both | 4 years | 45 | 84 | Diabetes | Incidence | Administrative medical records or disease registries; ; | 316 | 167080 | NA | NA |
| Swerdlow AJ | 2005 | Diabetes UK cohort | United Kingdom | prospective cohort | Both | NI | 30 | 49 | Diabetes | Mortality | Administrative medical records or disease registries; ; | 16 | 5066 | NA | NA |
|  |  |  |  |  |  |  |  |  |  | Incidence | Administrative medical records or disease registries; ; | 12 | 5066 | NA | NA |
| Coughlin SS | 2004 | Cancer Prevention Study II | United States of America | prospective cohort | Male | 16 years | 30 | 99 | Diabetes | Mortality | Administrative medical records or disease registries; Death certificates; | 2156 | 467922 | NA | NA |
|  |  |  |  |  | Female |  |  |  |  |  |  | 1950 | 588321 | NA | NA |
| Wideroff L | 1997 |  | Denmark | prospective cohort | Both | NI | 18 | 49 | Diabetes | Incidence | Administrative medical records or disease registries; ; | 13 | 109581 | NA | NA |
|  |  |  |  |  |  |  | 50 | 99 |  |  |  | 404 | 109581 | NA | NA |
| Verlato G | 2003 | Verona Diabetes study | Italy | prospective cohort | Male | 10 years | 25 | 99 | Diabetes | Mortality | Administrative medical records or disease registries; Death certificates; | 12 | 3366 | NA | NA |
|  |  |  |  |  | Female |  |  |  |  |  |  | 23 | 3782 | NA | NA |
| Liu X | 2012 |  | Sweden | prospective cohort | Both | 7 years | 40 | 99 | Diabetes | Mortality | Administrative medical records or disease registries; ; | 1065 | 1163 | NA | NA |
| Ulcickas Yood M | 2009 |  | United States of America | retrospective cohort | Both | 4 years | 18 | 99 | Diabetes | Incidence | Administrative medical records or disease registries; ; | 128 | 191223 | NA | NA |
| Larsson SC | 2005 | Cohort of Swedish Men | Sweden | prospective cohort | Male | 7 years | 45 | 79 | Diabetes | Incidence | Administrative medical records or disease registries; ; | 75 | 45906 | NA | NA |
|  |  | Swedish Mammography Cohort | Sweden | prospective cohort | Female | 7 years | 49 | 83 | Diabetes | Incidence | Administrative medical records or disease registries; ; | 61 | 37147 | NA | NA |
| Gupta S | 2006 |  | United States of America | retrospective cohort | Both | 6 years | 50 | 99 | Diabetes | Incidence | Administrative medical records or disease registries; ; | 2630 | 1421794 | NA | NA |
| Stevens Richard J | 2008 |  | United Kingdom | prospective cohort | Female | 8 years | 25 | 99 | Diabetes | Incidence & Mortality | Administrative medical records or disease registries; ; | 780 | 1290000 | NA | NA |
| El-Serag HB | 2009 |  | United States of America | retrospective cohort | Both | 2.3 years | 25 | 99 | Diabetes | Incidence | Administrative medical records or disease registries; ; | 617 | 718687 | NA | NA |
| Shibata Atsuko | 2006 |  | California | prospective cohort | Both | 9 years | 65 | 85 | Diabetes | Incidence & Mortality | Physician diagnosis; ; | 65 | 13976 | NA | NA |
| Er, KC | 2016 |  | Taiwan | prospective cohort | Both | 8.5 years | 18 | 99 | Diabetes | Incidence | Administrative medical records or disease registries; ; | 497 | 699115 | NA | NA |
| Kim SK | 2020 |  | Republic of Korea | retrospective cohort | Both | 8.6 years | 30 | 99 | Diabetes | Incidence | Administrative medical records or disease registries; ; | NI | 25,709,497 | NA | NA |
| Molina-Montes E | 2021 | European Study into Digestive Illnesses and Genetics (PanGenEU) | Western Europe | case-control | Both | NI | 18 | 99 | Diabetes | Incidence | Medical records; ; | 2018 | 3558 | 2018 | 1540 |
| Xia B | 2020 | UK Biobank cohort | United Kingdom | prospective cohort | Both | 6.6 years | 37 | 73 | FPG | Incidence | Medical records; ; | 565 | 475078 | NA | NA |
| Petrusel L | 2020 |  | Romania | case-control | Both | NI | 16 | 91 | Diabetes | Incidence | Imaging; ; | 279 | 591 | 279 | 312 |
| Farias AJ | 2020 | Multiethnic Cohort Study (MEC) | United States of America | nested case-control | Both | NI | 45 | 75 | Diabetes | Incidence | Medical records; ; | 433 | 2161] | 433 | 1728 |
| Kim, NH | 2020 | Kangbuk Samsung Health Study | Republic of Korea | prospective cohort | Both | 8.4 years | 18 | 99 | FPG | mortality | Death certificates; ; | 260 | 572021 | NA | NA |
| Van Tran T | 2021 |  | Viet Nam | prospective cohort | Both | NI | 18 | 99 | Diabetes | Incidence | Physician diagnosis; Imaging/scan; | 196 | 392 | 196 | 196 |
| Jacobson S | 2021 | Northern Sweden Health and Disease Study (NSHDS) | Sweden | nested case-control | Both | NI | 55 | 99 | FPG | Incidence | Administrative medical records or disease registries; ; | 182 | 899 | 182 | 717 |
| Dayem Ullah, A.Z.M | 2021 | EL-PaC-Epidem cohort | United Kingdom | case-control | Both | NI | 28 | 82 | Diabetes | Incidence | Medical records; ; | 140 | 294 | 140 | 154 |
|  | 2021 | EL-PaC-Epidem cohort | United Kingdom | case-control | Both | NI | 28 | 82 | Diabetes | Incidence | Medical records; ; | 44 | 209 | 44 | 165 |
|  | 2021 | EL-PaC-Epidem cohort | United Kingdom | case-control | Both | NI | 28 | 82 | Diabetes | Incidence | Medical records; ; | 176 | 902 | 176 | 726 |
| Chung, H.H | 2022 |  | Republic of Korea | case-control | Both | NI | 18 | 99 | FPG | Incidence | Imaging; ; | 51 | 237 | 51 | 181 |
| Davis TME | 2022 | Fremantle Diabetes Study Phase 1 (FDS1) | Australia | prospective cohort | Both | NI | 40 | 99 | Diabetes | Incidence | Medical records; ; | 67 | 6449 | NA | NA |
| Cai, J | 2022 | Kailuan cohort | China | prospective cohort | Male | 11 years | 18 | 71 | FPG | Incidence | Medical records; ; | 135 | 138870 | NA | NA |
| Ali S | 2022 |  | Australia | case-control | Female | NI | 18 | 88 | Diabetes | Incidence | Medical records; ; | 7267 | 43245 | 7267 | 35978 |
| Kim YJ | 2022 | Korean National Health Insurance System | Republic of Korea | prospective cohort | Both | NI | 18 | 99 | FPG | Incidence | Cancer registries; ; | 381 | 19050 | NA | NA |
| Shen B | 2022 | Shanghai Standardized Diabetes Management System (SSDMS) | China | prospective cohort | Both | 4.5 years | 18 | 99 | FPG | Incidence | Medical records; ; | 1056 | 428362 | NA | NA |
| Koo DH | 2023 | Korean National Health Insurance System | Republic of Korea | prospective cohort | Both | 6.2 years | 20 | 99 | FPG | Incidence | Administrative medical records or disease registries; ; | 3146 | 3138099 | NA | NA |
| Zhao, Q | 2023 | First Hospital of Shanxi Medical University | China | case-control | Both | NI | 18 | 99 | Diabetes | Incidence | Medical records; ; | 385 | 813 | 385 | 428 |
| Yang Z | 2023 | The Yinzhou Health Information System | China | retrospective cohort | Both | 9.2 years | 18 | 99 | Diabetes | Incidence | Administrative medical records or disease registries; ; | 15,729 | 208289 | NA | NA |
|  |  |  |  |  |  | 9.32 years |  |  |  | mortality | death certificates; ; | 5383 | 208289 | NA | NA |
| HernÃ¡ndez-GarduÃ±o E | 2021 |  | Mexico | prospective cohort | Both | NI | 20 | 99 | Diabetes | mortality | death certificates; ; | 17469 | 710292 | NA | NA |
| Pradhan R | 2022 | Clinical Practice Research Datalink (CPRD) | United Kingdom | prospective cohort | Both | 8.4 years | 18 | 99 | Diabetes | Incidence | National database; ; | 5832 | 1780428 | NA | NA |
| KirkegÃ¥rd J, | 2020 | Danish National Patient Registry, the Danish Cancer Registry, the Danish National Prescription Registry, the Danish Civil Registration System, and the Register of Laboratory Results for Research | Denmark | prospective cohort | Both | NI | 18 | 99 | Diabetes | Incidence | Cancer registries; ; | 283 | 28231 | NA | NA |
| Vicentini M | 2022 | Diabetes Registry and Population-based Cancer Registry | Italy | prospective cohort | Both | NI | 20 | 84 | Diabetes | Incidence | Cancer registries; ; | 616 | 401051 | NA | NA |
| Gurney J | 2022 | Statistics New Zealandâ€™s Integrated Data Infrastructure (IDI) | New Zealand | prospective cohort | Both | N | 18 | 99 | Diabetes | Incidence | Cancer registries; ; | 4875 | NI | NA | NA |
| Campbell PT | 2022 | Cancer Prevention Study-II (CPS-II) Nutrition Cohort | United States of America | case-control | Both | 10.4 years | 18 | 99 | HbA1C | Incidence | Cancer registries; medical records; | 62 | 831 | 44 | 530 |
| Tran TXM | 2023 | Korean National Health Insurance System | Republic of Korea | prospective cohort | Female | 10 years | 40 | 75 | FPG | mortality | National database; ; | 14186 | 8309393 | NA | NA |
| Goto A | 2020 | Japan Public Health Center-based Prospective (JPHC) Study. | Japan | case-control | Both | 15.9 years | 40 | 69 | FPG | Incidence | Cancer registries; death certificates; | 129 | 10536 | NA | NA |
| Hu Y | 2021 | Health Professionals Follow-up Study | United States of America | prospective cohort | Male | NI | 40 | 75 | FPG | Incidence | Medical records; ; | 382 | 45604 | NA | NA |
|  |  | Nurses Health Study (NHS) |  |  | Female | NI | 30 | 55 | FPG | Incidence | Medical records; ; | 667 | 113429 | NA | NA |
| MOMAYEZ SANAT, Zahra et al | 2021 |  | Iran (Islamic Republic of) | case-control | Both | NI | 18 | 99 | Diabetes | Incidence | Medical records; ; | 470 | 996 | 470 | 526 |
| Li P | 2023 |  | Finland | case-control | Both | NI | 20 | 99 | Diabetes | Incidence | Cancer registries; ; | 1523 | 4525 | 1523 | 3002 |
| Park SH | 2023 | No name - New Zealand study | New Zealand | prospective cohort | Both | NI | 18 | 99 | Diabetes | Incidence | Cancer registries; ; | 3474 | 4236350 | NA | NA |
| Bjornsdottir HH | 2020 | The Swedish National Diabetes Register (NDR) | Sweden | prospective cohort | Both | 6.6 years | 18 | 99 | Diabetes | Incidence | Death registries; ; | NI | 2744838 | NA | NA |
|  |  |  |  |  |  |  |  |  |  | Mortality | Death registries; ; | NI |  |  |  |
| Huang BZ | 2020 | Kaiser Permanente Southern California (KPSC) | United States of America | retrospective cohort | Both | NI | 45 | 90 | FPG | Incidence | Cancer registries; ; | 2002 | 1499627 | NA | NA |

3.6 Lung Cancer

| Author | Year of publication | Study Name | Location | Study Design | Sex | Follow-up | Age Start | Age End | Exposure Measure | Endpoint | Disease Ascertainment | Events | Sample Size | Cases | Controls |
| --- | --- | --- | --- | --- | --- | --- | --- | --- | --- | --- | --- | --- | --- | --- | --- |
| Jee SH | 2005 | Korean Cancer Prevention Study | Republic of Korea | prospective cohort | Male | 10 years | 30 | 95 | FPG | Mortality | Administrative medical records or disease registries | NI | 829770 | NA | NA |
|  | 2005 | Korean Cancer Prevention Study | Republic of Korea | prospective cohort | Female | 10 years | 30 | 95 | FPG | Mortality | Administrative medical records or disease registries | NI | 468615 | NA | NA |
|  | 2005 | Korean Cancer Prevention Study | Republic of Korea | prospective cohort | Male | 10 years | 30 | 95 | FPG | Incidence | Administrative medical records or disease registries | NI | 829770 | NA | NA |
|  | 2005 | Korean Cancer Prevention Study | Republic of Korea | prospective cohort | Female | 10 years | 30 | 95 | FPG | Incidence | Administrative medical records or disease registries | NI | 468615 | NA | NA |
| Pan XF | 2018 | China Kadoorie Biobank Study | China | prospective cohort | Both | 7.1 years | 30 | 79 | FPG | Incidence & Mortality | Administrative medical records or disease registries; Death certificates | 3282 | 479057 | NA | NA |
| Gathirua-Mwangi WG | 2017 | NHANES III | United States of America | prospective cohort | Both | 18 years | 18 | 99 | FPG | Mortality | Administrative medical records or disease registries; Death certificates; | 279 | 14916 | NA | NA |
| Park HJ | 2019 | National Health Insurance Services (NHIS) cohort | Republic of Korea | retrospective cohort | Both | 10 years | 40 | 99 | FPG | Incidence & Mortality | Administrative medical records or disease registries; ; | NI | 300518 | NA | NA |
| Hall GC | 2005 |  | United Kingdom | case-control | Both | 3.9 years | 25 | 99 | Diabetes | Incidence | Administrative medical records or disease registries; ; | 2659 | 334120 | 66848 | 267272 |
| Swerdlow AJ | 2005 | Diabetes UK cohort | United Kingdom | prospective cohort | Both | NI | 30 | 49 | Diabetes | Mortality | Administrative medical records or disease registries; ; | 67 | 5066 | NA | NA |
|  |  |  |  |  |  |  |  |  |  | Incidence |  | 56 | 5066 | NA | NA |
| Kuriki K, | 2007 |  | Japan | case-control | Male | NI | 40 | 80 | Diabetes | Incidence | Administrative medical records or disease registries; ; | 1101 | 19540 | 1101 | 14199 |
|  |  |  |  |  | Female | NI | 40 | 80 | Diabetes | Incidence |  | 419 | 39900 | 419 | 33569 |
| Johnson JA | 2011 |  | Canada | retrospective cohort | Both | 4.35 years | 30 | 99 | Diabetes | Incidence | Administrative medical records or disease registries; Physician diagnosis; | 3211 | 370200 | NA | NA |
| Wotton CJ | 2011 | ORLS1 | United Kingdom | prospective cohort | Both | NI | 30 | 99 | Diabetes | Incidence & Mortality | Administrative medical records or disease registries; Death certificates; | 4383 | 291462 | NA | NA |
|  |  | ORLS2 |  |  |  |  | 30 | 99 | Diabetes | Incidence & Mortality |  | 1057 | 192894 | NA | NA |
| Ogunleye AA | 2009 |  | Scotland | prospective cohort | Both | 3.9 years | 25 | 99 | Diabetes | Incidence & Mortality | Administrative medical records or disease registries; Death certificates; | 275 | 28731 | NA | NA |
| Atchison EA | 2011 |  | United States of America | prospective cohort | Male | 10.5 years | 18 | 100 | Diabetes | Incidence & Mortality | Administrative medical records or disease registries; ; | 102427 | 4501578 | NA | NA |
| Attner B | 2012 |  | Sweden | case-cohort | Both | 4 years | 45 | 84 | Diabetes | Incidence | Administrative medical records or disease registries; ; | 1623 | 167080 | NA | NA |
| Wideroff L | 1997 |  | Denmark | prospective cohort | Both | NI | 18 | 99 | Diabetes | Incidence | Administrative medical records or disease registries; ; | 963 | 109581 | NA | NA |
| Verlato G, | 2003 | Verona Diabetes study | Italy | prospective cohort | Male | 10 years | 25 | 99 | Diabetes | Mortality | Administrative medical records or disease registries; Death certificates; | 94 | 3366 | NA | NA |
|  |  |  |  |  | Female |  |  |  |  |  |  | 24 | 3782 | NA | NA |
| Liu X | 2012 |  | Sweden | prospective cohort | Both | 7 years | 40 | 99 | Diabetes | Mortality | Administrative medical records or disease registries; ; | 865 | 1093 | NA | NA |
| Campbell PT. | 2012 | Cancer Prevention Study II cohort | United States of America | prospective cohort | Male | 26 years | 30 | 99 | Diabetes | Mortality | Administrative medical records or disease registries; ; | 18605 | 467143 | NA | NA |
|  | 2012 | Cancer Prevention Study II cohort | United States of America | prospective cohort | Female | 26 years | 30 | 99 | Diabetes | Mortality | Administrative medical records or disease registries; ; | 11839 | 586688 | NA | NA |
| Steenland K | 1995 | NHANES I | United States of America | prospective cohort | Male | 7.7 years | 25 | 74 | Diabetes | Incidence & Mortality | Administrative medical records or disease registries; Death certificates; | 151 | 14407 | NA | NA |
|  |  |  |  |  | Female | 7.7 years | 25 | 74 | Diabetes | Incidence & Mortality | Administrative medical records or disease registries; Death certificates; | 59 | 14407 | NA | NA |
| Lee MY | 2012 |  | Taiwan (Province of China) | prospective cohort | Both | 11 years | 18 | 99 | Diabetes | Incidence | Administrative medical records or disease registries; ; | NI | 985815 | NA | NA |
| Kim SK | 2020 |  | Republic of Korea | retrospective cohort | Both | 8.6 years | 30 | 99 | Diabetes | Incidence | Administrative medical records or disease registries; ; | NI | 25,709,497 | NA | NA |
| Leiter A | 2021 | Prostate, Lung, Colorectal, and Ovarian (PLCO) Cancer Screening dataset | United States of America | retrospective cohort | Both | NI | 55 | 74 | Diabetes | Incidence | Cancer registries; medical records; | 3467 | 140935 | NA | NA |
| Yang Z | 2023 | The Yinzhou Health Information System | China | retrospective cohort | Both | 9.2 years | 18 | 99 | Diabetes | Incidence | Administrative medical records or disease registries; ; | 3737 | 208289 | NA | NA |
|  |  |  |  |  |  | 9.32 years | 18 | 99 | Diabetes | Mortality | Death certificates | 1418 | 208289 | NA | NA |
| Pradhan R | 2022 | Clinical Practice Research Datalink (CPRD) | United Kingdom | prospective cohort | Both | 8.4 years | 18 | 99 | Diabetes | Incidence | National database; ; | 18456 | 1780428 | NA | NA |
| Vicentini M | 2022 | Diabetes Registry and Population-based Cancer Registry | Italy | prospective cohort | Both | NI | 20 | 84 | Diabetes | Incidence | Cancer registries; ; | 1661 | 401051 | NA | NA |
| Yoo, T.K | 2022 | Kangbuk Samsung Health Study | Republic of Korea | prospective cohort | Both | 6.99 years | 18 | 99 | HbA1C | Mortality | Death certificates | 372 | 550931 | NA | NA |
| Gurney J | 2022 | Statistics New Zealandâ€™s Integrated Data Infrastructure (IDI) | New Zealand | prospective cohort | Both | NI | 18 | 99 | Diabetes | Incidence | Cancer registries; ; | 19740 | NI | NA | NA |
| Tran TXM | 2023 | Korean National Health Insurance System | Republic of Korea | prospective cohort | Female | 10 years | 40 | 75 | FPG | mortality | National database; ; | 34757 | 8309393 | NA | NA |
| Goto A | 2020 | Japan Public Health Center-based Prospective (JPHC) Study. | Japan | case-control | Both | 15.9 years | 40 | 69 | FPG | Incidence | Cancer registries; death certificates; | 437 | 10536 | NA | NA |
| Hu Y | 2021 | Health Professionals Follow-up Study | United States of America | prospective cohort | Male | NI | 40 | 75 | FPG | Incidence | Medical records; ; | 874 | 45604 | NA | NA |
|  | 2021 | Nurses Health Study (NHS) | United States of America | prospective cohort | Female | NI | 30 | 55 | FPG | Incidence | Medical records; ; | 2940 | 113429 | NA | NA |
| Mulla K | 2023 |  | United Kingdom | retrospective cohort | Both | NI | 18 | 99 | 2h-OGTT | Incidence | Medication use (pharmacy records); ; | 228 | 959 | NA | NA |
| Park SH | 2023 |  | New Zealand | prospective cohort | Both | NI | 18 | 99 | Diabetes | Incidence | Cancer registries; ; | 15663 | 4236350 | NA | NA |
| Bjornsdottir HH | 2020 | The Swedish National Diabetes Register (NDR) | Sweden | prospective cohort | Both | 6.6 years | 18 | 99 | Diabetes | Incidence | Death registries; ; | NI | 2744838 | NA | NA |
|  |  |  |  |  |  |  | 18 | 99 | Diabetes | Mortality | Death registries; ; | NI |  | NA | NA |
| Peila R | 2020 | UK Biobank cohort | United Kingdom | prospective cohort | Both | 7.1 years | 40 | 69 | HbA1C | Incidence | Medical records; National Database; | 2294 | 476517 | NA | NA |

3.7 Ovarian Cancer

| Author | Year of publication | Study Name | Location | Study Design | Sex | Follow-up | Age Start | Age End | Exposure Measure | Endpoint | Disease Ascertainment | Events | Sample Size | Cases | Controls |
| --- | --- | --- | --- | --- | --- | --- | --- | --- | --- | --- | --- | --- | --- | --- | --- |
| Yilmaz Kavcar SR | 2022 | Balcova Heart Study (BHS) | Turkey | prospective cohort | Female | 6 years | 30 | 99 | FPG | Incidence | Cancer registries; ; | 9 | 15869 | NA | NA |
| Pradhan R | 2022 | Clinical Practice Research Datalink (CPRD) | United Kingdom | prospective cohort | Female | 8.4 years | 18 | 99 | Diabetes | Incidence | National database; ; | 2095 | 1780428 | NA | NA |
| Vicentini M | 2022 | Diabetes Registry and Population-based Cancer Registry | Italy | prospective cohort | Both | NI | 20 | 84 | Diabetes | Incidence | Cancer registries; ; | 225 | 401051 | NA | NA |
| Gurney J | 2022 | Statistics New Zealand’s Integrated Data Infrastructure (IDI) | New Zealand | prospective cohort | Both | NI | 18 | 99 | Diabetes | Incidence | Cancer registries; ; | 2640 | NI | NA | NA |
| Campbell PT | 2022 | Cancer Prevention Study-II (CPS-II) Nutrition Cohort | United States of America | case-control | Both | 10.4 years | 18 | 99 | HbA1C | Incidence | Cancer registries; medical records; | 27 | 330 | 27 | 303 |
| Tran TXM | 2023 | Korean National Health Insurance System | Republic of Korea | prospective cohort | Female | 10 years | 40 | 75 | FPG | mortality | National database; ; | 11679 | 8309393 | NA | NA |
| Hu Y | 2021 | Nurses Health Study (NHS) | United States of America | prospective cohort | Female | NI | 30 | 55 | FPG | Incidence | Medical records; ; | 1160 | 113429 | NA | NA |
| Park SH | 2023 | No name - New Zealand study | New Zealand | prospective cohort | Female | NI | 18 | 99 | Diabetes | Incidence | Cancer registries; ; | NI | 4010235 | NA | NA |
| Kim K | 2023 | Korean National Health Insurance System | Republic of Korea | prospective cohort | Female | 9.3 years | 65 | 99 | FPG | Incidence | Medical records; ; | 1791 | 1032880 | NA | NA |
| Bjornsdottir HH | 2020 | The Swedish National Diabetes Register (NDR) | Sweden | case-control | Female | 6.6 years | 18 | 99 | Diabetes | Incidence | Death registries; ; | 457473 | 2744838 | 457473 | 2287365 |
| Peila R | 2020 | UK Biobank cohort | United Kingdom | prospective cohort | Female | 7.1 years | 40 | 69 | HbA1C | Incidence | Medical records; National Database; | 587 | 476517 | NA | NA |
| Attner B | 2012 | no name - Sweden 2007 study | Sweden | case-cohort | Female | 4 years | 45 | 84 | Diabetes | Incidence | Administrative medical records or disease registries; ; | NI | 167080 | NA | NA |
| Lo S-F | 2013 | no name - Taiwan 2009 another study | Taiwan (Province of China) | prospective cohort | Female | 3.5 years | 25 | 99 | Diabetes | Incidence | Administrative medical records or disease registries; ; | 948 | 1790868 | NA | NA |
| Parazzini F | 1997 | no name - italian study | Italy | case-control | Female | NI | 18 | 75 | Diabetes | Incidence | Administrative medical records or disease registries; ; | NI | 3729 | 971 | 2758 |
| Chodick G | 2010 | no name - Israel 2008 study | Israel | retrospective cohort | Female | 8 years | 21 | 99 | HbA1c | Incidence | Administrative medical records or disease registries; ; | 88 | 100585 | NA | NA |
| Johnson JA | 2011 | no name - Canada 2006 cohort study | Canada | retrospective cohort | Female | 4.35 years | 30 | 99 | Diabetes | Incidence | Administrative medical records or disease registries; Physician diagnosis; | 295 | 370200 | NA | NA |
| Lambe M | 2011 | AMORIS study | Stockholm | prospective cohort | Female | 11.7 years | 50 | 99 | FPG | Incidence | Administrative medical records or disease registries; ; | 783 | 230737 | NA | NA |
| Wotton CJ | 2011 | ORLS1 | United Kingdom | prospective cohort | Female | NI | 30 | 99 | Diabetes | Incidence | Administrative medical records or disease registries; Death certificates; | 476 | 132271 | NA | NA |
|  |  | ORLS2 |  |  |  |  | 30 | 99 | Diabetes | Incidence | Administrative medical records or disease registries; Death certificates; | 194 | 90427 | NA | NA |
| Coughlin SS | 2004 | Cancer Prevention Study II | United States of America | prospective cohort | Female | 16 years | 30 | 99 | Diabetes | Mortality | Administrative medical records or disease registries; Death certificates; | 1739 | 588321 | NA | NA |
| Wideroff L | 1997 | no name - Denmark 1969 study | Denmark | prospective cohort | Female | NI | 18 | 49 | Diabetes | Incidence | Administrative medical records or disease registries; ; | 129 | 55010 | NA | NA |
| Swerdlow AJ | 2005 | Diabetes UK cohort | United Kingdom | prospective cohort | Female | NI | 30 | 49 | Diabetes | Mortality | Administrative medical records or disease registries; ; | 6 | 2122 | NA | NA |
|  |  |  |  |  |  |  |  |  |  | Incidence | Administrative medical records or disease registries; ; | 6 | 2122 | NA | NA |
| Kabat GC | 2018 | Women's Health Initiative | United States of America | prospective cohort | Female | 14.7 yeats | 50 | 79 | FPG | Incidence | Self-report; Administrative medical records or disease registries; | 130 | 21103 | NA | NA |

## Section 4: Study quality and bias covariates

The risk of bias of each study was assessed during the extraction process based on a list of bias covariates. All bias covariates were dichotomous variables that assessed 1) representativeness of the study population; 2) exposure and outcome measurement quality; 3) control for confounding; 4) selection bias; and 5) risk of reverse causation. All covariates were tested for significance using the selection algorithm in the MR-BRT tool.

### Table S6. Study quality for every study used in the models

| **Bias Category** | **Description** | **Values** |
| --- | --- | --- |
| Representativeness of study population | Representativeness of general population | 0 = representative  1 = not representative |
| Exposure measurement quality | Study definition of diabetes, description of blood assessments | 0 = individual, objective measure, multiple prospective measures  1 = not clear reporting |
| Outcome quality | Author definition of cancer outcomes, databases and method of diagnosis | 0 = death or medical records/physician diagnosis  1 = self-reported cancer |
| Confounding quality | Variables considered for adjustments for each type of cancer | 0 = not adjusting for confounders  1 = adjusted for confounders |
| Selection bias | Risk of selection bias can be identified through losses to follow-up (cohorts) or percent for whom data not  ascertained (case-controls) >  20%. | 0 = no selection bias  1 = probable selection bias |
| Risk of reverse causation |  |  |

### Table S7. Variables considered for defining good confounder quality for each cancer

| **Cancer Type** | **Variables considered** |
| --- | --- |
| Bladder Cancer | Age, sex, smoking, alcohol use, body mass index |
| Liver Cancer | Age, sex, smoking, alcohol use, body mass index |
| Colorectal Cancer | Age, sex, smoking, alcohol use, body mass index |
| Pancreas Cancer | Age, sex, smoking, alcohol use, body mass index |
| Breast Cancer | Age, sex, smoking, alcohol use, body mass index, family history of breast cancer |
| Lung Cancer | Age, sex, smoking, alcohol use, body mass index |
| Ovarian Cancer | Age, sex, smoking, alcohol use, body mass index |

## Section 5: Results from individual studies

### Table S8. Summary results from input studies, with reference and alternative group exposure for each study

| **Outcome** | **Reference group exposure** | **Alternative group exposure** | **Log effect size** | **Log effect size standard error** | **Study** |
| --- | --- | --- | --- | --- | --- |
| Bladder Cancer | 5.28-6.99 mmol/L | 7-7.77 mmol/L | 0.2 | 0.05 | Gurney J, 2022 |
| Bladder Cancer | 5.28-6.99 mmol/L | 7-7.77 mmol/L | 0.19 | 0.16 | Park SH, 2023 |
| Bladder Cancer | 5.28-6.99 mmol/L | 7-7.77 mmol/L | 0.23 | 0.29 | Park SH 2023 |
| Bladder Cancer | 5.28-6.99 mmol/L | 7-7.77 mmol/L | 0.27 | 0.39 | Park SH 2023 |
| Bladder Cancer | 5.28-6.99 mmol/L | 7-7.77 mmol/L | 0.7 | 0.35 | Park SH 2023 |
| Bladder Cancer | 5.28-6.99 mmol/L | 7-7.77 mmol/L | 0.28 | 0.05 | Park SH 2023 |
| Bladder Cancer | 4.6-5.56 mmol/L | 5.56-6.94 mmol/L | -0.05 | 0.49 | Chung H, 2009 |
| Bladder Cancer | 4.6-5.56 mmol/L | 7-8.18 mmol/L | 0.8 | 0.66 | Chung H, 2009 |
| Bladder Cancer | 5.08-6.99 mmol/L | 7-8.18 mmol/L | 0.31 | 0.11 | Tseng C-H, 2011 |
| Bladder Cancer | 5.08-6.99 mmol/L | 7-8.18 mmol/L | 0.21 | 0.04 | Lo S-F, 2013 |
| Bladder Cancer | 5.08-6.99 mmol/L | 7-8.18 mmol/L | 0.18 | 0.35 | Chen HF, 2015 |
| Bladder Cancer | 5.08-6.99 mmol/L | 7-8.18 mmol/L | 0.09 | 0.07 | Chen HF, 2015 |
| Bladder Cancer | 5.08-6.99 mmol/L | 7-8.18 mmol/L | 0.12 | 0.05 | Chen HF, 2015 |
| Bladder Cancer | 5.08-6.99 mmol/L | 7-8.18 mmol/L | -0.19 | 0.5 | Chen HF, 2015 |
| Bladder Cancer | 5.08-6.99 mmol/L | 7-8.18 mmol/L | 0.08 | 0.09 | Chen HF, 2015 |
| Bladder Cancer | 5.08-6.99 mmol/L | 7-8.18 mmol/L | 0.01 | 0.07 | Chen HF, 2015 |
| Bladder Cancer | 5.08-6.99 mmol/L | 7-8.18 mmol/L | 1.5 | 0.3 | Tseng CH, 2009 |
| Bladder Cancer | 5.08-6.99 mmol/L | 7-8.18 mmol/L | 0.9 | 0.17 | Tseng CH, 2009 |
| Bladder Cancer | 5.08-6.99 mmol/L | 7-8.18 mmol/L | 0.56 | 0.23 | Tseng CH, 2009 |
| Bladder Cancer | 5.08-6.99 mmol/L | 7-8.18 mmol/L | 2.05 | 0.29 | Tseng CH, 2009 |
| Bladder Cancer | 5.08-6.99 mmol/L | 7-8.18 mmol/L | 0.72 | 0.24 | Tseng CH, 2009 |
| Bladder Cancer | 5.08-6.99 mmol/L | 7-8.18 mmol/L | 0.56 | 0.26 | Tseng CH, 2009 |
| Bladder Cancer | 4.2-5 mmol/L | 5-6.06 mmol/L | 0.2 | 0.15 | Jee SH, 2005 |
| Bladder Cancer | 4.2-5 mmol/L | 6.06-6.94 mmol/L | 0.11 | 0.26 | Jee SH, 2005 |
| Bladder Cancer | 4.2-5 mmol/L | 5-6.06 mmol/L | 0.08 | 0.06 | Jee SH, 2005 |
| Bladder Cancer | 4.2-5 mmol/L | 6.06-6.94 mmol/L | 0.06 | 0.18 | Jee SH, 2005 |
| Bladder Cancer | 4.2-5 mmol/L | 7-7.72 mmol/L | 0.31 | 0.17 | Jee SH, 2005 |
| Bladder Cancer | 4.2-5 mmol/L | 7-7.83 mmol/L | 0.45 | 0.23 | Jee SH, 2005 |
| Bladder Cancer | 4.2-5 mmol/L | 7.78-8.5 mmol/L | 0.21 | 0.12 | Jee SH, 2005 |
| Bladder Cancer | 4.8-5.56 mmol/L | 5.56-6.94 mmol/L | 0.04 | 0.02 | Choi YH, 2022 |
| Bladder Cancer | 5.32-6.99 mmol/L | 7-7.83 mmol/L | 0.46 | 0.4 | Kuriki K, 2007 |
| Bladder Cancer | 5.32-6.99 mmol/L | 7-7.83 mmol/L | 0.25 | 0.02 | Kim SK, 2020 |
| Bladder Cancer | 5.32-6.99 mmol/L | 7-7.83 mmol/L | 0.26 | 0.03 | Kim SK, 2020 |
| Bladder Cancer | 5.32-6.99 mmol/L | 7-7.83 mmol/L | 0.21 | 0.04 | Kim SK, 2020 |
| Bladder Cancer | 5.32-7 mmol/L | 7-7.83 mmol/L | 0.19 | 0.02 | Choi YH, 2022 |
| Bladder Cancer | 4.92-6.13 mmol/L | 6.14-7 mmol/L | 0.07 | 0.15 | Campbell PT, 2022 |
| Bladder Cancer | 4.92-6.13 mmol/L | 7-8.93 mmol/L | 0.22 | 0.21 | Campbell PT, 2022 |
| Bladder Cancer | 5.06-6.99 mmol/L | 7-8.93 mmol/L | 0.46 | 0.21 | Risch HA, 1988 |
| Bladder Cancer | 5.06-6.99 mmol/L | 7-8.93 mmol/L | -0.09 | 0.14 | Hu Y, 2021 |
| Bladder Cancer | 5.06-6.99 mmol/L | 7-8.93 mmol/L | 0.19 | 0.17 | Hu Y, 2021 |
| Bladder Cancer | 5.06-6.99 mmol/L | 7-8.93 mmol/L | 0.14 | 0.08 | Bogumil D, 2023 |
| Bladder Cancer | 5.06-6.99 mmol/L | 7-8.93 mmol/L | -0.04 | 0.02 | Atchison EA, 2011 |
| Bladder Cancer | 5.06-6.99 mmol/L | 7-8.93 mmol/L | 0.36 | 0.12 | Coughlin SS, 2004 |
| Bladder Cancer | 5.06-6.99 mmol/L | 7-8.93 mmol/L | 0.26 | 0.22 | Coughlin SS, 2004 |
| Bladder Cancer | 5.06-6.99 mmol/L | 7-8.93 mmol/L | 0.85 | 0.15 | Ulcickas Yood M, 2009 |
| Bladder Cancer | 5.06-6.99 mmol/L | 7-8.93 mmol/L | 0.79 | 0.27 | MacKenzie T,2011 |
| Bladder Cancer | 4.59-7 mmol/L | 7-9.44 mmol/L | 0.11 | 0.67 | Yilmaz Kavcar SR, 2022 |
| Bladder Cancer | 4.59-7 mmol/L | 7-9.44 mmol/L | -0.46 | 1.11 | Yilmaz Kavcar SR, 2022 |
| Bladder Cancer | 5.37-6.16 mmol/L | 6.16-7.05 mmol/L | 0.24 | 0.1 | Peila R, 2020 |
| Bladder Cancer | 5.37-6.16 mmol/L | 5.37-7.04 mmol/L | 0.08 | 0.15 | Peila R, 2020 |
| Bladder Cancer | 4.88-6.99 mmol/L | 7-8.52 mmol/L | 0 | 0.05 | Wideroff L, 1997 |
| Bladder Cancer | 4.88-6.99 mmol/L | 7-8.52 mmol/L | -0.11 | 0.08 | Wideroff L, 1997 |
| Bladder Cancer | 4.88-6.99 mmol/L | 7-8.52 mmol/L | -0.11 | 0.22 | Wideroff L, 1997 |
| Bladder Cancer | 4.88-6.99 mmol/L | 7-8.52 mmol/L | 0 | 0.05 | Wideroff L, 1997 |
| Bladder Cancer | 4.88-6.99 mmol/L | 7-8.52 mmol/L | 0.85 | 0.2 | Kravchick S, 2001 |
| Bladder Cancer | 4.88-6.99 mmol/L | 7-8.52 mmol/L | 0.01 | 0.15 | Barone B, 2023 |
| Bladder Cancer | 4.88-6.99 mmol/L | 7-8.52 mmol/L | 0.25 | 0.08 | Vicentini M, 2022 |
| Bladder Cancer | 4.88-6.99 mmol/L | 7-8.52 mmol/L | 0.29 | 0.23 | Verlato G, 2003 |
| Bladder Cancer | 4.88-6.99 mmol/L | 7-8.52 mmol/L | 0.41 | 0.45 | Verlato G, 2003 |
| Bladder Cancer | 4.88-6.99 mmol/L | 7-8.52 mmol/L | 0.18 | 0.02 | Bjornsdottir HH, 2020 |
| Bladder Cancer | 4.88-6.99 mmol/L | 7-8.52 mmol/L | 0.11 | 0.03 | Bjornsdottir HH, 2020 |
| Bladder Cancer | 4.88-6.99 mmol/L | 7-8.52 mmol/L | 0.19 | 0.13 | Attner B, 2012 |
| Bladder Cancer | 4.88-6.99 mmol/L | 7-8.52 mmol/L | 0.29 | 0.06 | Liu X, 2012 |
| Bladder Cancer | 4.88-6.99 mmol/L | 7-8.52 mmol/L | 0.25 | 0.02 | Pradhan R, 2022 |
| Bladder Cancer | 5.37-6.16 mmol/L | 7.05-8.52 mmol/L | 0.4 | 0.15 | Peila R, 2020 |
| Bladder Cancer | 4.88-6.99 mmol/L | 7-8.52 mmol/L | -0.4 | 0.14 | Wotton CJ, 2011 |
| Bladder Cancer | 4.88-6.99 mmol/L | 7-8.52 mmol/L | 0.04 | 0.18 | Wotton CJ, 2011 |
| Bladder Cancer | 4.88-6.99 mmol/L | 7-8.52 mmol/L | 0.99 | 0.5 | Ng Y, 2003 |
| Bladder Cancer | 4.88-6.99 mmol/L | 7-8.52 mmol/L | -0.94 | 0.68 | Swerdlow AJ, 2005 |
| Bladder Cancer | 4.88-6.99 mmol/L | 7-8.52 mmol/L | 0 | 0.24 | Swerdlow AJ, 2005 |
| Bladder Cancer | 4.88-6.99 mmol/L | 7-8.52 mmol/L | -0.63 | 0.4 | Ogunleye AA, 2009 |
| Breast Cancer | 5.28-6.99 mmol/L | 7-7.77 mmol/L | 0.15 | 0.02 | Gurney J, 2022 |
| Breast Cancer | 5.28-6.99 mmol/L | 7-7.77 mmol/L | 0.27 | 0.04 | Park SH, 2023 |
| Breast Cancer | 5.28-6.99 mmol/L | 7-7.77 mmol/L | 0.23 | 0.07 | Park SH, 2023 |
| Breast Cancer | 5.28-6.99 mmol/L | 7-7.77 mmol/L | 0.17 | 0.11 | Park SH, 2023 |
| Breast Cancer | 5.28-6.99 mmol/L | 7-7.77 mmol/L | 0.2 | 0.13 | Park SH, 2023 |
| Breast Cancer | 5.28-6.99 mmol/L | 7-7.77 mmol/L | 0.17 | 0.03 | Park SH, 2023 |
| Breast Cancer | 4.6-5.5 mmol/L | 5.6-6.9 mmol/L | 0.05 | 0.06 | Pan XF, 2018 |
| Breast Cancer | 4.6-5.5 mmol/L | 7-8.18 mmol/L | 0.26 | 0.08 | Pan XF, 2018 |
| Breast Cancer | 5.08-6.99 mmol/L | 7-8.18 mmol/L | 0.17 | 0.19 | Yang Z, 2023 |
| Breast Cancer | 5.08-6.99 mmol/L | 7-8.18 mmol/L | 0.15 | 0.13 | Yang Z, 2023 |
| Breast Cancer | 5.08-6.99 mmol/L | 7-8.18 mmol/L | -0.26 | 0.18 | Yang Z, 2023 |
| Breast Cancer | 5.08-6.99 mmol/L | 7-8.18 mmol/L | 0.2 | 0.29 | Yang Z, 2023 |
| Breast Cancer | 5.08-6.99 mmol/L | 7-8.18 mmol/L | 0.21 | 0.03 | Lo S-F, 2013 |
| Breast Cancer | 5.08-6.99 mmol/L | 7-8.18 mmol/L | 0.89 | 0.09 | Tseng CH, 2009 |
| Breast Cancer | 5.08-6.99 mmol/L | 7-8.18 mmol/L | 0.31 | 0.09 | Tseng CH, 2009 |
| Breast Cancer | 5.08-6.99 mmol/L | 7-8.18 mmol/L | 0.69 | 0.08 | Tseng CH, 2009 |
| Breast Cancer | 5.08-6.99 mmol/L | 7-8.18 mmol/L | 0.69 | 0.12 | Tseng CH, 2009 |
| Breast Cancer | 5.08-6.99 mmol/L | 7-8.18 mmol/L | 1.18 | 0.16 | Tseng CH, 2009 |
| Breast Cancer | 5.08-6.99 mmol/L | 7-8.18 mmol/L | 0.31 | 0.14 | Tseng CH, 2009 |
| Breast Cancer | 5.08-6.99 mmol/L | 7-8.18 mmol/L | 0.69 | 0.13 | Tseng CH, 2009 |
| Breast Cancer | 5.08-6.99 mmol/L | 7-8.18 mmol/L | 0.9 | 0.2 | Tseng CH, 2009 |
| Breast Cancer | 5.08-6.99 mmol/L | 7-8.18 mmol/L | 0.01 | 0.16 | Lee MY, 2012 |
| Breast Cancer | 3.23-5.49 mmol/L | 5.6-5.71 mmol/L | 0.12 | 0.51 | Yoo, TK 2022 |
| Breast Cancer | 3.23-5.49 mmol/L | 5.83-5.91 mmol/L | 0.25 | 0.54 | Yoo, TK 2022 |
| Breast Cancer | 3.23-5.49 mmol/L | 5.92-6.03 mmol/L | 0.31 | 0.48 | Yoo, TK 2022 |
| Breast Cancer | 3.23-5.49 mmol/L | 6.14-6.89 mmol/L | 0.31 | 0.49 | Yoo, TK 2022 |
| Breast Cancer | 4.2-5 mmol/L | 5-6.06 mmol/L | 0.14 | 0.13 | Jee SH, 2005 |
| Breast Cancer | 4.2-5 mmol/L | 6.06-6.94 mmol/L | -0.12 | 0.3 | Jee SH, 2005 |
| Breast Cancer | 4.2-5 mmol/L | 5-6.06 mmol/L | 0.1 | 0.1 | Jee SH, 2005 |
| Breast Cancer | 4.2-5 mmol/L | 6.06-6.94 mmol/L | 0.11 | 0.1 | Jee SH, 2005 |
| Breast Cancer | 4.2-5 mmol/L | 7-7.72 mmol/L | 0.05 | 0.2 | Jee SH, 2005 |
| Breast Cancer | 4.2-5 mmol/L | 7-7.83 mmol/L | 0.22 | 0.28 | Jee SH, 2005 |
| Breast Cancer | 4.2-5 mmol/L | 7.78-8.5 mmol/L | -0.2 | 0.17 | Jee SH, 2005 |
| Breast Cancer | 4.8-5.56 mmol/L | 5.56-6.94 mmol/L | -0.06 | 0.06 | Lee SH, 2020 |
| Breast Cancer | 4.8-5.56 mmol/L | 5.56-6.94 mmol/L | 0.11 | 0.04 | Tran TXM, 2023 |
| Breast Cancer | 4.8-5.56 mmol/L | 7-7.83 mmol/L | 0.32 | 0.06 | Tran TXM, 2023 |
| Breast Cancer | 5.32-7 mmol/L | 7-7.83 mmol/L | -0.31 | 0.42 | Goto A, 2020 |
| Breast Cancer | 5.32-6.99 mmol/L | 7-7.83 mmol/L | 0.15 | 0.13 | Kuriki K, 2007 |
| Breast Cancer | 5.32-6.99 mmol/L | 7-7.83 mmol/L | 0.72 | 0.25 | Goodman MT, 1997 |
| Breast Cancer | 5.32-6.99 mmol/L | 7-7.83 mmol/L | 0.07 | 0.02 | Kim SK, 2020 |
| Breast Cancer | 3.98-4.97 mmol/L | 4.97-5.53 mmol/L | 0.27 | 0.2 | Kabat GC, 2009 |
| Breast Cancer | 3.98-4.97 mmol/L | 4.97-5.53 mmol/L | 0.03 | 0.26 | Kabat GC, 2009 |
| Breast Cancer | 3.98-4.97 mmol/L | 4.97-5.53 mmol/L | 1.17 | 0.59 | Kabat GC, 2009 |
| Breast Cancer | 3.98-4.97 mmol/L | 4.97-5.53 mmol/L | 0.46 | 0.28 | Kabat GC, 2009 |
| Breast Cancer | 3.98-4.97 mmol/L | 5.53-7.2 mmol/L | 0.34 | 0.23 | Kabat GC, 2009 |
| Breast Cancer | 3.98-4.97 mmol/L | 5.53-7.2 mmol/L | 0.13 | 0.28 | Kabat GC, 2009 |
| Breast Cancer | 3.98-4.97 mmol/L | 5.53-7.2 mmol/L | 1.25 | 0.62 | Kabat GC, 2009 |
| Breast Cancer | 3.98-4.97 mmol/L | 5.53-7.2 mmol/L | 0.17 | 0.38 | Kabat GC, 2009 |
| Breast Cancer | 4.58-5.56 mmol/L | 5.56-6.94 mmol/L | 0.21 | 0.17 | Mink PJ, 2022 |
| Breast Cancer | 4.58-5.56 mmol/L | 5.56-6.94 mmol/L | 0.33 | 0.24 | Mink PJ, 2022 |
| Breast Cancer | 3.33-5.56 mmol/L | 5.56-7.2 mmol/L | 0.17 | 0.07 | Zimbalist, 2022 |
| Breast Cancer | 4.92-6.13 mmol/L | 6.14-7 mmol/L | -0.01 | 0.12 | Campbell PT, 2022 |
| Breast Cancer | 4.92-6.13 mmol/L | 7-8.93 mmol/L | -0.13 | 0.19 | Campbell PT, 2022 |
| Breast Cancer | 5.06-6.99 mmol/L | 7-8.93 mmol/L | 0 | 0.05 | Johnson JA, 2011 |
| Breast Cancer | 5.06-6.99 mmol/L | 7-8.93 mmol/L | 0.07 | 0.04 | Lipscombe LL, 2006 |
| Breast Cancer | 5.06-6.99 mmol/L | 7-8.93 mmol/L | 0.23 | 0.04 | Hu Y, 2021 |
| Breast Cancer | 5.06-6.99 mmol/L | 7-8.93 mmol/L | 0.3 | 0.08 | Hossain FM, 2022 |
| Breast Cancer | 5.06-6.99 mmol/L | 7-8.93 mmol/L | 0.36 | 0.2 | Hossain FM, 2022 |
| Breast Cancer | 5.06-6.99 mmol/L | 7-8.93 mmol/L | 0.59 | 0.14 | Hossain FM, 2022 |
| Breast Cancer | 5.06-6.99 mmol/L | 7-8.93 mmol/L | 0.52 | 0.25 | Hossain FM, 2022 |
| Breast Cancer | 5.06-6.99 mmol/L | 7-8.93 mmol/L | 0.24 | 0.07 | Coughlin SS, 2994 |
| Breast Cancer | 5.06-6.99 mmol/L | 7-8.93 mmol/L | 0.1 | 0.07 | Baron JA, 2001 |
| Breast Cancer | 5.06-6.99 mmol/L | 7-8.93 mmol/L | 0.12 | 0.25 | Weiss HA, 1999 |
| Breast Cancer | 5.06-6.99 mmol/L | 7-8.93 mmol/L | 0.34 | 0.35 | Steenland K, 1995 |
| Breast Cancer | 5.06-6.99 mmol/L | 7-8.93 mmol/L | -0.08 | 0.1 | Bosco JL, 2012 |
| Breast Cancer | 5.06-6.99 mmol/L | 7-8.93 mmol/L | -0.07 | 0.24 | Bosco JL, 2012 |
| Breast Cancer | 5.06-6.99 mmol/L | 7-8.93 mmol/L | -0.07 | 0.12 | Bosco JL, 2012 |
| Breast Cancer | 5.06-6.99 mmol/L | 7-8.93 mmol/L | 0.54 | 0.2 | Wu AH, 2007 |
| Breast Cancer | 5.06-6.99 mmol/L | 7-8.93 mmol/L | -0.14 | 0.21 | Sanderson M, 2010 |
| Breast Cancer | 4.59-7 mmol/L | 7-9.44 mmol/L | -0.3 | 0.39 | Yilmaz Kavcar SR, 2022 |
| Breast Cancer | 4.72-6.11 mmol/L | 6.11-7.74 mmol/L | 0.77 | 0.35 | Haseen SD, 2015 |
| Breast Cancer | 4.72-6.11 mmol/L | 6.11-7.74 mmol/L | 0.16 | 0.29 | Haseen SD, 2015 |
| Breast Cancer | 4.84-6.99 mmol/L | 7-8.73 mmol/L | 0.47 | 0.4 | Garmendia ML, 2007 |
| Breast Cancer | 4.84-6.99 mmol/L | 7-8.73 mmol/L | 0.62 | 0.47 | Garmendia ML, 2007 |
| Breast Cancer | 4.84-6.99 mmol/L | 7-8.73 mmol/L | 0.27 | 0.41 | Ronco AL, 2012 |
| Breast Cancer | 5.37-6.16 mmol/L | 6.16-7.05 mmol/L | -0.31 | 0.14 | Peila R, 2020 |
| Breast Cancer | 5.37-6.16 mmol/L | 6.16-7.05 mmol/L | -0.08 | 0.04 | Peila R, 2020 |
| Breast Cancer | 5.37-6.16 mmol/L | 5.37-7.04 mmol/L | -0.04 | 0.06 | Peila R, 2020 |
| Breast Cancer | 5.37-6.16 mmol/L | 5.37-7.04 mmol/L | 0.12 | 0.04 | Peila R, 2020 |
| Breast Cancer | 4.41-5.6 mmol/L | 5.6-7.04 mmol/L | 0.22 | 0.16 | Melvin JC, 2017 |
| Breast Cancer | 4.88-6.99 mmol/L | 7-8.52 mmol/L | 0.1 | 0.43 | Wideroff L, 1997 |
| Breast Cancer | 4.88-6.99 mmol/L | 7-8.52 mmol/L | 0.1 | 0.02 | Wideroff L, 1997 |
| Breast Cancer | 4.88-6.99 mmol/L | 7-8.52 mmol/L | -0.11 | 0.12 | Wideroff L, 1997 |
| Breast Cancer | 4.88-6.99 mmol/L | 7-8.52 mmol/L | 0.18 | 0.02 | Wideroff L, 1997 |
| Breast Cancer | 4.88-6.99 mmol/L | 7-8.52 mmol/L | 0.26 | 0.69 | Chodick G, 2010 |
| Breast Cancer | 4.88-6.99 mmol/L | 7-8.52 mmol/L | 0 | 0.08 | Chodick G, 2010 |
| Breast Cancer | 4.88-6.99 mmol/L | 7-8.52 mmol/L | -0.03 | 0.09 | Vicentini M, 2022 |
| Breast Cancer | 4.88-6.99 mmol/L | 7-8.52 mmol/L | 0.34 | 0.14 | Verlato G, 2003 |
| Breast Cancer | 4.88-6.99 mmol/L | 7-8.52 mmol/L | 0.9 | 0.29 | Crispo A, 2017 |
| Breast Cancer | 4.88-6.99 mmol/L | 7-8.52 mmol/L | 0.05 | 0.02 | Bjornsdottir HH, 2020 |
| Breast Cancer | 4.88-6.99 mmol/L | 7-8.52 mmol/L | -0.02 | 0.04 | Bjornsdottir HH, 2020 |
| Breast Cancer | 4.88-6.99 mmol/L | 7-8.52 mmol/L | 0.13 | 0.58 | Attner B, 2012 |
| Breast Cancer | 4.88-6.99 mmol/L | 7-8.52 mmol/L | 0.29 | 0.18 | Attner B, 2012 |
| Breast Cancer | 4.88-6.99 mmol/L | 7-8.52 mmol/L | 0.37 | 0.05 | Liu X, 2012 |
| Breast Cancer | 4.88-6.99 mmol/L | 7-8.52 mmol/L | 0.09 | 0.02 | Pradhan R, 2022 |
| Breast Cancer | 5.37-6.16 mmol/L | 7.05-8.52 mmol/L | -0.16 | 0.25 | Peila R, 2020 |
| Breast Cancer | 5.37-6.16 mmol/L | 7.05-8.52 mmol/L | 0.07 | 0.09 | Peila R, 2020 |
| Breast Cancer | 4.88-6.99 mmol/L | 7-8.52 mmol/L | 0.05 | 0.09 | Wotton CJ, 2011 |
| Breast Cancer | 4.88-6.99 mmol/L | 7-8.52 mmol/L | -0.04 | 0.15 | Wotton CJ, 2011 |
| Breast Cancer | 4.88-6.99 mmol/L | 7-8.52 mmol/L | -0.15 | 0.26 | Swerdlow AJ, 2005 |
| Breast Cancer | 4.88-6.99 mmol/L | 7-8.52 mmol/L | -0.14 | 0.16 | Swerdlow AJ, 2005 |
| Breast Cancer | 4.88-6.99 mmol/L | 7-8.52 mmol/L | 0.05 | 0.2 | Ogunleye AA, 2009 |
| Breast Cancer | 5.01-7.53 mmol/L | 7.54-8.62 mmol/L | 0.02 | 0.2 | Zhang F, 2023 |
| Breast Cancer | 5.01-7.53 mmol/L | 8.62-9.17 mmol/L | -0.2 | 0.27 | Zhang F, 2023 |
| Colorectal Cancer | 5.28-6.99 mmol/L | 7-7.77 mmol/L | 1.48 | 0.4 | Mikaeel RR, 2021 |
| Colorectal Cancer | 5.28-6.99 mmol/L | 7-7.77 mmol/L | 0.2 | 0.02 | Gurney J, 2022 |
| Colorectal Cancer | 5.28-6.99 mmol/L | 7-7.77 mmol/L | 0.21 | 0.06 | Park SH, 2023 |
| Colorectal Cancer | 5.28-6.99 mmol/L | 7-7.77 mmol/L | 0.14 | 0.09 | Park SH, 2023 |
| Colorectal Cancer | 5.28-6.99 mmol/L | 7-7.77 mmol/L | 0.29 | 0.16 | Park SH, 2023 |
| Colorectal Cancer | 5.28-6.99 mmol/L | 7-7.77 mmol/L | 0.41 | 0.1 | Park SH, 2023 |
| Colorectal Cancer | 5.28-6.99 mmol/L | 7-7.77 mmol/L | 0.26 | 0.02 | Park SH, 2023 |
| Colorectal Cancer | 4.84-6.99 mmol/L | 7-8.83 mmol/L | 0.05 | 0.02 | Herold M, 2023 |
| Colorectal Cancer | 4.84-6.99 mmol/L | 7-8.83 mmol/L | 0.06 | 0.02 | Herold M, 2023 |
| Colorectal Cancer | 4.84-6.99 mmol/L | 7-8.83 mmol/L | 0.13 | 0.04 | Herold M, 2023 |
| Colorectal Cancer | 4.84-6.99 mmol/L | 7-8.83 mmol/L | 0.13 | 0.04 | Herold M, 2023 |
| Colorectal Cancer | 4.6-5.5 mmol/L | 5.6-6.9 mmol/L | 0.05 | 0.06 | Pan XF, 2018 |
| Colorectal Cancer | 4.6-5.5 mmol/L | 7-8.18 mmol/L | 0.21 | 0.07 | Pan XF, 2018 |
| Colorectal Cancer | 5.08-6.99 mmol/L | 7-8.18 mmol/L | 0.29 | 0.23 | Yang Z, 2023 |
| Colorectal Cancer | 5.08-6.99 mmol/L | 7-8.18 mmol/L | 0.19 | 0.12 | Yang Z, 2023 |
| Colorectal Cancer | 5.08-6.99 mmol/L | 7-8.18 mmol/L | 0.06 | 0.11 | Yang Z, 2023 |
| Colorectal Cancer | 5.08-6.99 mmol/L | 7-8.18 mmol/L | -0.02 | 0.12 | Yang Z, 2023 |
| Colorectal Cancer | 5.08-6.99 mmol/L | 7-8.18 mmol/L | 1.05 | 0.9 | Yang Z, 2023 |
| Colorectal Cancer | 5.08-6.99 mmol/L | 7-8.18 mmol/L | 0.29 | 0.28 | Yang Z, 2023 |
| Colorectal Cancer | 5.08-6.99 mmol/L | 7-8.18 mmol/L | 0.03 | 0.22 | Yang Z, 2023 |
| Colorectal Cancer | 5.08-6.99 mmol/L | 7-8.18 mmol/L | -0.15 | 0.18 | Yang Z, 2023 |
| Colorectal Cancer | 5.08-7 mmol/L | 7-8.18 mmol/L | 0.9 | 0.28 | Hsu S, 2022 |
| Colorectal Cancer | 5.08-6.99 mmol/L | 7-8.18 mmol/L | 0.91 | 0.27 | Wu Po-Hsien Mda, 2023 |
| Colorectal Cancer | 5.08-6.99 mmol/L | 7-8.18 mmol/L | 0.2 | 0.02 | Lo S-F, 2013 |
| Colorectal Cancer | 5.08-6.99 mmol/L | 7-8.18 mmol/L | 0.56 | 0.08 | Lee MY, 2012 |
| Colorectal Cancer | 3.23-5.49 mmol/L | 5.6-5.71 mmol/L | 0.9 | 0.51 | Yoo TK, 2022 |
| Colorectal Cancer | 3.23-5.49 mmol/L | 5.83-5.91 mmol/L | 0.49 | 0.57 | Yoo TK, 2022 |
| Colorectal Cancer | 3.23-5.49 mmol/L | 5.92-6.03 mmol/L | 0.79 | 0.5 | Yoo TK, 2022 |
| Colorectal Cancer | 3.23-5.49 mmol/L | 6.14-6.89 mmol/L | 1.25 | 0.48 | Yoo TK, 2022 |
| Colorectal Cancer | 4.2-5 mmol/L | 5-6.06 mmol/L | 0.07 | 0.06 | Jee SH, 2005 |
| Colorectal Cancer | 4.2-5 mmol/L | 6.06-6.94 mmol/L | 0.24 | 0.1 | Jee SH, 2005 |
| Colorectal Cancer | 4.2-5 mmol/L | 7-7.72 mmol/L | 0.21 | 0.19 | Jee SH, 2005 |
| Colorectal Cancer | 4.2-5 mmol/L | 5-6.06 mmol/L | -0.04 | 0.09 | Jee SH, 2005 |
| Colorectal Cancer | 4.2-5 mmol/L | 6.06-6.94 mmol/L | 0.05 | 0.17 | Jee SH, 2005 |
| Colorectal Cancer | 4.2-5 mmol/L | 5-6.06 mmol/L | 0.08 | 0.03 | Jee SH, 2005 |
| Colorectal Cancer | 4.2-5 mmol/L | 6.06-6.94 mmol/L | 0.13 | 0.06 | Jee SH, 2005 |
| Colorectal Cancer | 4.2-5 mmol/L | 7-7.72 mmol/L | 0.03 | 0.11 | Jee SH, 2005 |
| Colorectal Cancer | 4.2-5 mmol/L | 5-6.06 mmol/L | 0.04 | 0.05 | Jee SH, 2005 |
| Colorectal Cancer | 4.2-5 mmol/L | 6.06-6.94 mmol/L | 0.1 | 0.1 | Jee SH, 2005 |
| Colorectal Cancer | 4.2-5 mmol/L | 7-7.72 mmol/L | -0.22 | 0.21 | Jee SH, 2005 |
| Colorectal Cancer | 4.2-4.94 mmol/L | 5-6.06 mmol/L | 0.69 | 0.4 | Chung YW, 2006 |
| Colorectal Cancer | 4.2-4.94 mmol/L | 6.11-7.13 mmol/L | 1.1 | 0.61 | Chung YW, 2006 |
| Colorectal Cancer | 4.2-5 mmol/L | 7-7.83 mmol/L | -0.16 | 0.19 | Jee SH, 2005 |
| Colorectal Cancer | 4.2-5 mmol/L | 7.78-8.5 mmol/L | 0.27 | 0.12 | Jee SH, 2005 |
| Colorectal Cancer | 4.2-5 mmol/L | 7.78-8.5 mmol/L | 0.12 | 0.07 | Jee SH, 2005 |
| Colorectal Cancer | 4.2-5 mmol/L | 7.78-8.5 mmol/L | 0.07 | 0.12 | Jee SH, 2005 |
| Colorectal Cancer | 4.8-5.56 mmol/L | 5.56-6.06 mmol/L | 0.1 | 0.03 | Hidetaka I, 2021 |
| Colorectal Cancer | 4.8-5.56 mmol/L | 6.11-6.94 mmol/L | 0.22 | 0.05 | Hidetaka I, 2021 |
| Colorectal Cancer | 4.8-5.28 mmol/L | 5.33-5.83 mmol/L | 0 | 0.27 | Yamada K, 1998 |
| Colorectal Cancer | 4.8-5.28 mmol/L | 5.89-6.39 mmol/L | -0.36 | 0.41 | Yamada K, 1998 |
| Colorectal Cancer | 4.8-5.56 mmol/L | 5.56-6.94 mmol/L | 0.08 | 0.01 | Kim DB, 2021 |
| Colorectal Cancer | 4.8-5.56 mmol/L | 5.56-6.06 mmol/L | 0.1 | 0.18 | Jung KJ, 2016 |
| Colorectal Cancer | 4.8-5.56 mmol/L | 6.11-6.94 mmol/L | 0.59 | 0.2 | Jung KJ, 2016 |
| Colorectal Cancer | 4.8-5.56 mmol/L | 5.56-6.06 mmol/L | 0.18 | 0.26 | Jung KJ, 2016 |
| Colorectal Cancer | 4.8-5.56 mmol/L | 6.11-6.94 mmol/L | 0.53 | 0.26 | Jung KJ, 2016 |
| Colorectal Cancer | 4.8-5.56 mmol/L | 5.56-6.06 mmol/L | 0.1 | 0.25 | Jung KJ, 2016 |
| Colorectal Cancer | 4.8-5.56 mmol/L | 6.11-6.94 mmol/L | 0.64 | 0.27 | Jung KJ, 2016 |
| Colorectal Cancer | 4.8-5.28 mmol/L | 6.44-7.13 mmol/L | 0.69 | 0.4 | Yamada K, 1998 |
| Colorectal Cancer | 4.8-5.56 mmol/L | 6.11-7.13 mmol/L | 0.22 | 0.27 | Kim J, 2020 |
| Colorectal Cancer | 4.8-5.56 mmol/L | 7-7.83 mmol/L | 0.31 | 0.07 | Hidetaka I, 2021 |
| Colorectal Cancer | 4.8-5.56 mmol/L | 7-7.83 mmol/L | 0.21 | 0.01 | Kim DB, 2021 |
| Colorectal Cancer | 5.12-6.06 mmol/L | 7-7.83 mmol/L | 0.45 | 0.26 | Wu Jingjing Mda, 2021 |
| Colorectal Cancer | 5.32-7 mmol/L | 7-7.83 mmol/L | 0.05 | 0.16 | Goto A, 2020 |
| Colorectal Cancer | 5.32-6.99 mmol/L | 7-7.83 mmol/L | 0.26 | 0.13 | Kuriki K, 2007 |
| Colorectal Cancer | 5.32-6.99 mmol/L | 7-7.83 mmol/L | 0.28 | 0.17 | Kuriki K, 2007 |
| Colorectal Cancer | 5.32-6.99 mmol/L | 7-7.83 mmol/L | 0.25 | 0.2 | Kuriki K, 2007 |
| Colorectal Cancer | 5.32-6.99 mmol/L | 7-7.83 mmol/L | 0.12 | 0.23 | Kuriki K, 2007 |
| Colorectal Cancer | 5.32-6.99 mmol/L | 7-7.83 mmol/L | -0.06 | 0.3 | Kuriki K, 2007 |
| Colorectal Cancer | 5.32-6.99 mmol/L | 7-7.83 mmol/L | 0.39 | 0.34 | Kuriki K, 2007 |
| Colorectal Cancer | 5.32-6.99 mmol/L | 7-7.83 mmol/L | 0.19 | 0.01 | Kim SK, 2020 |
| Colorectal Cancer | 5.32-6.99 mmol/L | 7-7.83 mmol/L | 0.19 | 0.01 | Kim SK, 2020 |
| Colorectal Cancer | 5.32-6.99 mmol/L | 7-7.83 mmol/L | 0.18 | 0.01 | Kim SK, 2020 |
| Colorectal Cancer | 5.32-7 mmol/L | 7-7.83 mmol/L | 0.51 | 0.26 | Wu Jingjing Mda, 2021 |
| Colorectal Cancer | 5.32-7 mmol/L | 7-7.83 mmol/L | -0.36 | 0.18 | Shin A, 2011 |
| Colorectal Cancer | 5.32-7 mmol/L | 7-7.83 mmol/L | 0.26 | 0.11 | Shin A, 2011 |
| Colorectal Cancer | 5.32-7 mmol/L | 7-7.83 mmol/L | 0.26 | 0.08 | Shin A, 2011 |
| Colorectal Cancer | 5.32-7 mmol/L | 7-7.83 mmol/L | 0.18 | 0.22 | Shin A, 2011 |
| Colorectal Cancer | 5.32-7 mmol/L | 7-7.83 mmol/L | -0.22 | 0.32 | Shin A, 2011 |
| Colorectal Cancer | 5.32-7 mmol/L | 7-7.83 mmol/L | 0.18 | 0.15 | Shin A, 2011 |
| Colorectal Cancer | 3.98-4.97 mmol/L | 4.97-5.53 mmol/L | 0.28 | 0.31 | Kabat GC, 2012 |
| Colorectal Cancer | 3.98-4.97 mmol/L | 4.97-5.53 mmol/L | 0.54 | 0.36 | Kabat GC, 2012 |
| Colorectal Cancer | 3.98-4.97 mmol/L | 5.53-7.2 mmol/L | 0.55 | 0.3 | Kabat GC, 2012 |
| Colorectal Cancer | 3.98-4.97 mmol/L | 5.53-7.2 mmol/L | 0.81 | 0.36 | Kabat GC, 2012 |
| Colorectal Cancer | 4.92-6.13 mmol/L | 6.14-7 mmol/L | 0.05 | 0.13 | Campbell PT, 2022 |
| Colorectal Cancer | 4.92-6.13 mmol/L | 7-8.93 mmol/L | 0.41 | 0.19 | Campbell PT, 2022 |
| Colorectal Cancer | 5.06-6.99 mmol/L | 7-8.93 mmol/L | 0.04 | 0.03 | Qiang JK, 2020 |
| Colorectal Cancer | 5.06-6.99 mmol/L | 7-8.93 mmol/L | -0.03 | 0.06 | Qiang JK, 2020 |
| Colorectal Cancer | 5.06-6.99 mmol/L | 7-8.93 mmol/L | 0.06 | 0.03 | Qiang JK, 2020 |
| Colorectal Cancer | 5.06-6.99 mmol/L | 7-8.93 mmol/L | 0.56 | 0.57 | Chang VC, 2021 |
| Colorectal Cancer | 5.06-6.99 mmol/L | 7-8.93 mmol/L | 0.22 | 0.04 | Johnson JA, 2011 |
| Colorectal Cancer | 5.06-6.99 mmol/L | 7-8.93 mmol/L | -0.05 | 0.13 | Low EE, 2020 |
| Colorectal Cancer | 5.06-6.99 mmol/L | 7-8.93 mmol/L | 0.28 | 0.11 | Hu Y, 2021 |
| Colorectal Cancer | 5.06-6.99 mmol/L | 7-8.93 mmol/L | 0.12 | 0.09 | Hu Y, 2021 |
| Colorectal Cancer | 5.06-6.99 mmol/L | 7-8.93 mmol/L | 1.06 | 0.01 | Boustany A, 2023 |
| Colorectal Cancer | 5.06-6.99 mmol/L | 7-8.93 mmol/L | 0.43 | 0.77 | Joseph DF, 2021 |
| Colorectal Cancer | 5.06-6.99 mmol/L | 7-8.93 mmol/L | 0.26 | 0.12 | Will JC, 1998 |
| Colorectal Cancer | 5.06-6.99 mmol/L | 7-8.93 mmol/L | 0.15 | 0.14 | Will JC, 1998 |
| Colorectal Cancer | 5.06-6.99 mmol/L | 7-8.93 mmol/L | 0.18 | 0.07 | Coughlin SS, 2004 |
| Colorectal Cancer | 5.06-6.99 mmol/L | 7-8.93 mmol/L | 0.22 | 0.07 | Coughlin SS, 2004 |
| Colorectal Cancer | 5.06-6.99 mmol/L | 7-8.93 mmol/L | 0.07 | 0.18 | Coughlin SS, 2004 |
| Colorectal Cancer | 5.06-6.99 mmol/L | 7-8.93 mmol/L | -0.11 | 0.23 | Coughlin SS, 2004 |
| Colorectal Cancer | 5.06-6.99 mmol/L | 7-8.93 mmol/L | 0.18 | 0.02 | Atchison EA, 2011 |
| Colorectal Cancer | 5.06-6.99 mmol/L | 7-8.93 mmol/L | 0.11 | 0.02 | Atchison EA, 2011 |
| Colorectal Cancer | 5.06-6.99 mmol/L | 7-8.93 mmol/L | 0.69 | 0.14 | Ulcickas Yood M, 2009 |
| Colorectal Cancer | 5.06-6.99 mmol/L | 7-8.93 mmol/L | 0.36 | 0.43 | Steenland K, 1995 |
| Colorectal Cancer | 5.06-6.99 mmol/L | 7-8.93 mmol/L | 0.34 | 0.4 | Steenland K, 1995 |
| Colorectal Cancer | 5.35-7.53 mmol/L | 5.35-7.53 mmol/L | -0.29 | 0.19 | Schumacher AJ, 2021 |
| Colorectal Cancer | 5.35-7.53 mmol/L | 7.54-9.27 mmol/L | -0.05 | 0.18 | Schumacher AJ, 2021 |
| Colorectal Cancer | 4.59-7 mmol/L | 7-9.44 mmol/L | -0.03 | 0.66 | Yilmaz Kavcar SR, 2022 |
| Colorectal Cancer | 4.59-7 mmol/L | 7-9.44 mmol/L | -1.14 | 1.06 | Yilmaz Kavcar SR, 2022 |
| Colorectal Cancer | 3.3-4.5 mmol/L | 4.6-4.8 mmol/L | 0.22 | 0.42 | Vulcan A, 2017 |
| Colorectal Cancer | 3.3-4.5 mmol/L | 4.9-5.2 mmol/L | 0.71 | 0.39 | Vulcan A, 2017 |
| Colorectal Cancer | 3.3-4.5 mmol/L | 5.3-16.8 mmol/L | 1.03 | 0.36 | Vulcan A, 2017 |
| Colorectal Cancer | 3.4-4.5 mmol/L | 4.6-4.8 mmol/L | 0.22 | 0.34 | Vulcan A, 2017 |
| Colorectal Cancer | 3.4-4.5 mmol/L | 4.9-5.2 mmol/L | -0.12 | 0.32 | Vulcan A, 2017 |
| Colorectal Cancer | 3.4-4.5 mmol/L | 5.2-12.2 mmol/L | 0.15 | 0.32 | Vulcan A, 2017 |
| Colorectal Cancer | 3.3-4.5 mmol/L | 4.6-4.8 mmol/L | 0.67 | 0.63 | Vulcan A, 2017 |
| Colorectal Cancer | 3.3-4.5 mmol/L | 4.9-5.2 mmol/L | 0.73 | 0.63 | Vulcan A, 2017 |
| Colorectal Cancer | 3.3-4.5 mmol/L | 5.3-16.8 mmol/L | 1.44 | 0.57 | Vulcan A, 2017 |
| Colorectal Cancer | 3.4-4.5 mmol/L | 4.6-4.8 mmol/L | -0.31 | 0.51 | Vulcan A, 2017 |
| Colorectal Cancer | 3.4-4.5 mmol/L | 4.9-5.2 mmol/L | -0.04 | 0.39 | Vulcan A, 2017 |
| Colorectal Cancer | 3.4-4.5 mmol/L | 5.3-12.2 mmol/L | 0.01 | 0.43 | Vulcan A, 2017 |
| Colorectal Cancer | 3.89-5.11 mmol/L | 5.17-5.44 mmol/L | 0.17 | 0.37 | Limburg PJ, 2006 |
| Colorectal Cancer | 3.89-5.11 mmol/L | 5.5-5.94 mmol/L | 0.67 | 0.36 | Limburg PJ, 2006 |
| Colorectal Cancer | 3.89-4.4 mmol/L | 4.4-4.8 mmol/L | 0.13 | 0.07 | Wulaningsih W, 2012 |
| Colorectal Cancer | 3.89-4.4 mmol/L | 4.8-5.2 mmol/L | 0.11 | 0.07 | Wulaningsih W, 2012 |
| Colorectal Cancer | 3.89-4.4 mmol/L | 4.4-4.8 mmol/L | -0.16 | 0.08 | Wulaningsih W, 2012 |
| Colorectal Cancer | 3.89-4.4 mmol/L | 4.8-5.2 mmol/L | -0.26 | 0.09 | Wulaningsih W, 2012 |
| Colorectal Cancer | 3.89-5.11 mmol/L | 5.94-7.04 mmol/L | 0.5 | 0.38 | Limburg PJ, 2006 |
| Colorectal Cancer | 3.89-4.4 mmol/L | 5.2-7.04 mmol/L | 0.19 | 0.07 | Wulaningsih W, 2012 |
| Colorectal Cancer | 3.89-4.4 mmol/L | 5.2-7.04 mmol/L | -0.13 | 0.09 | Wulaningsih W, 2012 |
| Colorectal Cancer | 4.68-6.13 mmol/L | 6.14-7.71 mmol/L | 0.13 | 0.05 | Rothwell JA, 2022 |
| Colorectal Cancer | 4.88-6.99 mmol/L | 7-8.52 mmol/L | 0.26 | 0.06 | Wideroff L, 1997 |
| Colorectal Cancer | 4.88-6.99 mmol/L | 7-8.52 mmol/L | 0.1 | 0.05 | Wideroff L, 1997 |
| Colorectal Cancer | 4.88-6.99 mmol/L | 7-8.52 mmol/L | 0.18 | 0.02 | Wideroff L, 1997 |
| Colorectal Cancer | 4.88-6.99 mmol/L | 7-8.52 mmol/L | 0.1 | 0.07 | Wideroff L, 1997 |
| Colorectal Cancer | 4.88-6.99 mmol/L | 7-8.52 mmol/L | 0 | 0.07 | Wideroff L, 1997 |
| Colorectal Cancer | 4.88-6.99 mmol/L | 7-8.52 mmol/L | 0.18 | 0.22 | Wideroff L, 1997 |
| Colorectal Cancer | 4.88-6.99 mmol/L | 7-8.52 mmol/L | 0 | 0.05 | Wideroff L, 1997 |
| Colorectal Cancer | 4.88-6.99 mmol/L | 7-8.52 mmol/L | 0.13 | 0.12 | Chodick G, 2010 |
| Colorectal Cancer | 4.88-6.99 mmol/L | 7-8.52 mmol/L | 0.42 | 0.13 | Chodick G, 2010 |
| Colorectal Cancer | 4.88-6.99 mmol/L | 7-8.52 mmol/L | 0.1 | 0.2 | Chodick G, 2010 |
| Colorectal Cancer | 4.88-6.99 mmol/L | 7-8.52 mmol/L | 0.08 | 0.23 | Chodick G, 2010 |
| Colorectal Cancer | 4.88-6.99 mmol/L | 7-8.52 mmol/L | 0.17 | 0.08 | Vicentini M, 2022 |
| Colorectal Cancer | 4.88-6.99 mmol/L | 7-8.52 mmol/L | 0.17 | 0.16 | Verlato G, 2003 |
| Colorectal Cancer | 4.88-6.99 mmol/L | 7-8.52 mmol/L | -0.25 | 0.21 | Verlato G, 2003 |
| Colorectal Cancer | 4.88-6.99 mmol/L | 7-8.52 mmol/L | 0.18 | 0.01 | Bjornsdottir HH, 2020 |
| Colorectal Cancer | 4.88-6.99 mmol/L | 7-8.52 mmol/L | 0.09 | 0.02 | Bjornsdottir HH, 2020 |
| Colorectal Cancer | 4.88-6.99 mmol/L | 7-8.52 mmol/L | 0.42 | 0.14 | Attner B, 2012 |
| Colorectal Cancer | 4.88-6.99 mmol/L | 7-8.52 mmol/L | 0.38 | 0.13 | Attner B, 2012 |
| Colorectal Cancer | 4.88-6.99 mmol/L | 7-8.52 mmol/L | 0.49 | 0.32 | Attner B, 2012 |
| Colorectal Cancer | 4.88-6.99 mmol/L | 7-8.52 mmol/L | 0.39 | 0.09 | Attner B, 2012 |
| Colorectal Cancer | 4.88-6.99 mmol/L | 7-8.52 mmol/L | 0.22 | 0.17 | Attner B, 2012 |
| Colorectal Cancer | 4.88-6.99 mmol/L | 7-8.52 mmol/L | 0.29 | 0.04 | Liu X, 2012 |
| Colorectal Cancer | 4.88-6.99 mmol/L | 7-8.52 mmol/L | 0.16 | 0.05 | Liu X, 2012 |
| Colorectal Cancer | 4.88-6.99 mmol/L | 7-8.52 mmol/L | 0.14 | 0.07 | Corcoran NM, 2022 |
| Colorectal Cancer | 4.88-6.99 mmol/L | 7-8.52 mmol/L | 0.28 | 0.13 | Corcoran NM, 2022 |
| Colorectal Cancer | 4.88-6.99 mmol/L | 7-8.52 mmol/L | 0.23 | 0.01 | Pradhan R, 2022 |
| Colorectal Cancer | 4.88-6.99 mmol/L | 7-8.52 mmol/L | 0.31 | 0.08 | Yang YX, 2005 |
| Colorectal Cancer | 4.88-6.99 mmol/L | 7-8.52 mmol/L | 0.38 | 0.1 | Yang YX, 2005 |
| Colorectal Cancer | 4.88-6.99 mmol/L | 7-8.52 mmol/L | 0.19 | 0.15 | Yang YX, 2005 |
| Colorectal Cancer | 4.88-6.99 mmol/L | 7-8.52 mmol/L | 0.32 | 0.1 | Yang YX, 2005 |
| Colorectal Cancer | 4.88-6.99 mmol/L | 7-8.52 mmol/L | 0.31 | 0.13 | Yang YX, 2005 |
| Colorectal Cancer | 4.88-6.99 mmol/L | 7-8.52 mmol/L | 0.19 | 0.15 | Yang YX, 2005 |
| Colorectal Cancer | 4.88-6.99 mmol/L | 7-8.52 mmol/L | 0.01 | 0.2 | Swerdlow AJ, 2005 |
| Colorectal Cancer | 4.88-6.99 mmol/L | 7-8.52 mmol/L | 0.1 | 0.14 | Swerdlow AJ, 2005 |
| Colorectal Cancer | 4.88-6.99 mmol/L | 7-8.52 mmol/L | 0.06 | 0.09 | Wotton CJ, 2011 |
| Colorectal Cancer | 4.88-6.99 mmol/L | 7-8.52 mmol/L | 0.17 | 0.16 | Wotton CJ, 2011 |
| Colorectal Cancer | 4.88-6.99 mmol/L | 7-8.52 mmol/L | 0.08 | 0.12 | Wotton CJ, 2011 |
| Colorectal Cancer | 4.88-6.99 mmol/L | 7-8.52 mmol/L | 0.13 | 0.23 | Wotton CJ, 2011 |
| Colorectal Cancer | 4.88-6.99 mmol/L | 7-8.52 mmol/L | 0.44 | 0.2 | Ogunleye AA, 2009 |
| Colorectal Cancer | 4.88-6.99 mmol/L | 7-8.52 mmol/L | -0.63 | 0.38 | Ogunleye AA, 2009 |
| Colorectal Cancer | 5.01-8 mmol/L | 8-9.17 mmol/L | -0.11 | 0.22 | Nilsen TIL, 2001 |
| Colorectal Cancer | 5.01-8 mmol/L | 8-9.17 mmol/L | 0.68 | 0.21 | Nilsen TIL, 2001 |
| Colorectal Cancer | 5.01-8 mmol/L | 8-9.17 mmol/L | -0.42 | 0.38 | Nilsen TIL, 2001 |
| Colorectal Cancer | 5.01-8 mmol/L | 8-9.17 mmol/L | 0.65 | 0.3 | Nilsen TIL, 2001 |
| Colorectal Cancer | 5.01-8 mmol/L | 8-9.17 mmol/L | -0.11 | 0.22 | Nilsen TIL, 2002 |
| Colorectal Cancer | 5.01-8 mmol/L | 8-9.17 mmol/L | 0.68 | 0.21 | Nilsen TIL, 2002 |
| Liver Cancer | 5.28-6.99 mmol/L | 7-7.77 mmol/L | 1.21 | 0.04 | Gurney J, 2022 |
| Liver Cancer | 5.28-6.99 mmol/L | 7-7.77 mmol/L | 0.84 | 0.09 | Park SH, 2023 |
| Liver Cancer | 5.28-6.99 mmol/L | 7-7.77 mmol/L | 0.43 | 0.14 | Park SH, 2023 |
| Liver Cancer | 5.28-6.99 mmol/L | 7-7.77 mmol/L | 1.39 | 0.42 | Park SH, 2023 |
| Liver Cancer | 5.28-6.99 mmol/L | 7-7.77 mmol/L | 0.65 | 0.18 | Park SH, 2023 |
| Liver Cancer | 5.28-6.99 mmol/L | 7-7.77 mmol/L | 1.28 | 0.05 | Park SH, 2023 |
| Liver Cancer | 4.78-6.99 mmol/L | 7-9.26 mmol/L | 0.46 | 0.01 | HernÃ¡ndez-GarduÃ±o E, 2021 |
| Liver Cancer | 3.9-6.1 mmol/L | 2.5-3.9 mmol/L | -0.01 | 0.39 | Feng X, 2017 |
| Liver Cancer | 3.9-6.1 mmol/L | 6.1-7 mmol/L | 0.47 | 0.2 | Feng X, 2017 |
| Liver Cancer | 4.6-5.5 mmol/L | 5.6-6.9 mmol/L | 0.1 | 0.06 | Pan XF, 2018 |
| Liver Cancer | 4.6-5.56 mmol/L | 5.56-6.94 mmol/L | 0.26 | 0.45 | Chen CL, 2020 |
| Liver Cancer | 4.6-5.56 mmol/L | 5.56-6.94 mmol/L | 0.64 | 0.34 | Chen CL, 2020 |
| Liver Cancer | 4.6-5.5 mmol/L | 7-8.18 mmol/L | 0.36 | 0.06 | Pan XF, 2018 |
| Liver Cancer | 4.6-5.56 mmol/L | 7-8.18 mmol/L | 0.53 | 0.41 | Chen CL, 2020 |
| Liver Cancer | 4.6-5.56 mmol/L | 7-8.18 mmol/L | 1.24 | 0.3 | Chen CL, 2020 |
| Liver Cancer | 4.89-6.09 mmol/L | 6.1-7 mmol/L | 0.65 | 0.31 | Zhang L, 2023 |
| Liver Cancer | 4.89-6.11 mmol/L | 6.11-6.94 mmol/L | 0.34 | 0.29 | Chao LT, 2011 |
| Liver Cancer | 4.89-5.99 mmol/L | 6-7.31 mmol/L | 0.39 | 0.13 | Ma X, 2021 |
| Liver Cancer | 4.89-6.09 mmol/L | 7-8.18 mmol/L | 0.75 | 0.27 | Zhang L, 2023 |
| Liver Cancer | 4.89-6.11 mmol/L | 7-8.18 mmol/L | 0.86 | 0.38 | Chao LT, 2011 |
| Liver Cancer | 5.08-6.99 mmol/L | 7-8.18 mmol/L | 0.59 | 0.2 | Yang Z, 2023 |
| Liver Cancer | 5.08-6.99 mmol/L | 7-8.18 mmol/L | 0.49 | 0.12 | Yang Z, 2023 |
| Liver Cancer | 5.08-6.99 mmol/L | 7-8.18 mmol/L | 0.42 | 0.12 | Yang Z, 2023 |
| Liver Cancer | 5.08-6.99 mmol/L | 7-8.18 mmol/L | 0.48 | 0.16 | Yang Z, 2023 |
| Liver Cancer | 5.08-6.99 mmol/L | 7-8.18 mmol/L | 0.9 | 0.3 | Yang Z, 2023 |
| Liver Cancer | 5.08-6.99 mmol/L | 7-8.18 mmol/L | 0.43 | 0.18 | Yang Z, 2023 |
| Liver Cancer | 5.08-6.99 mmol/L | 7-8.18 mmol/L | 0.36 | 0.16 | Yang Z, 2023 |
| Liver Cancer | 5.08-6.99 mmol/L | 7-8.18 mmol/L | 0.44 | 0.17 | Yang Z, 2023 |
| Liver Cancer | 5.08-6.99 mmol/L | 7-8.18 mmol/L | 0.49 | 0.42 | Lai M-S, 2006 |
| Liver Cancer | 5.08-6.99 mmol/L | 7-8.18 mmol/L | 0.6 | 0.02 | Lo S-F, 2013 |
| Liver Cancer | 5.08-6.99 mmol/L | 7-8.18 mmol/L | 0.51 | 0.09 | Lee MY, 2012 |
| Liver Cancer | 3.9-6.1 mmol/L | 7-8.18 mmol/L | 0.46 | 0.2 | Feng X, 2017 |
| Liver Cancer | 5.56-6.61 mmol/L | 4.44-5.5 mmol/L | 0.06 | 0.06 | Yoo JJ, 2021 |
| Liver Cancer | 5.56-6.61 mmol/L | 6.67-7.72 mmol/L | -0.01 | 0.05 | Yoo JJ, 2021 |
| Liver Cancer | 5.56-6.61 mmol/L | 8.28-8.83 mmol/L | -0.02 | 0.05 | Yoo JJ, 2021 |
| Liver Cancer | 5.56-6.61 mmol/L | 8.89-9.94 mmol/L | 0.08 | 0.06 | Yoo JJ, 2021 |
| Liver Cancer | 3.23-5.49 mmol/L | 5.6-5.71 mmol/L | -0.16 | 0.24 | Yoo TK, 2022 |
| Liver Cancer | 3.23-5.49 mmol/L | 5.83-5.91 mmol/L | -0.25 | 0.27 | Yoo TK, 2022 |
| Liver Cancer | 3.23-5.49 mmol/L | 5.92-6.03 mmol/L | -0.45 | 0.23 | Yoo TK, 2022 |
| Liver Cancer | 3.23-5.49 mmol/L | 6.14-6.89 mmol/L | -0.71 | 0.24 | Yoo TK, 2022 |
| Liver Cancer | 5.56-6.61 mmol/L | 4.2-4.44 mmol/L | -0.01 | 0.1 | Yoo JJ, 2021 |
| Liver Cancer | 4.2-5 mmol/L | 5-6.06 mmol/L | 0.04 | 0.03 | Jee SH, 2005 |
| Liver Cancer | 4.2-5 mmol/L | 6.06-6.94 mmol/L | 0.22 | 0.05 | Jee SH, 2005 |
| Liver Cancer | 4.2-5 mmol/L | 7-7.72 mmol/L | 0.52 | 0.09 | Jee SH, 2005 |
| Liver Cancer | 4.2-5 mmol/L | 5-6.06 mmol/L | -0.16 | 0.08 | Jee SH, 2005 |
| Liver Cancer | 4.2-5 mmol/L | 6.06-6.94 mmol/L | 0.04 | 0.15 | Jee SH, 2005 |
| Liver Cancer | 4.2-5 mmol/L | 7-7.72 mmol/L | 0.2 | 0.25 | Jee SH, 2005 |
| Liver Cancer | 4.2-5 mmol/L | 5-6.06 mmol/L | 0.01 | 0.03 | Jee SH, 2005 |
| Liver Cancer | 4.2-5 mmol/L | 6.06-6.94 mmol/L | 0.15 | 0.04 | Jee SH, 2005 |
| Liver Cancer | 4.2-5 mmol/L | 7-7.72 mmol/L | 0.37 | 0.08 | Jee SH, 2005 |
| Liver Cancer | 4.2-5 mmol/L | 5-6.06 mmol/L | -0.11 | 0.07 | Jee SH, 2005 |
| Liver Cancer | 4.2-5 mmol/L | 6.06-6.94 mmol/L | 0.21 | 0.11 | Jee SH, 2005 |
| Liver Cancer | 4.2-5 mmol/L | 7-7.72 mmol/L | 0.05 | 0.08 | Jee SH, 2005 |
| Liver Cancer | 4.2-5 mmol/L | 5-6.06 mmol/L | 0.03 | 0.02 | Kim K, 2018 |
| Liver Cancer | 4.2-5 mmol/L | 6.11-6.94 mmol/L | 0.14 | 0.03 | Yang Z, 2023 |
| Liver Cancer | 4.2-5 mmol/L | 7-7.72 mmol/L | 0.19 | 0.06 | Yang Z, 2023 |
| Liver Cancer | 4.2-5 mmol/L | 7.78-8.5 mmol/L | 0.45 | 0.06 | Jee SH, 2005 |
| Liver Cancer | 4.2-5 mmol/L | 7.78-8.5 mmol/L | 0.29 | 0.15 | Jee SH, 2005 |
| Liver Cancer | 4.2-5 mmol/L | 7.78-8.5 mmol/L | 0.54 | 0.05 | Jee SH, 2005 |
| Liver Cancer | 4.2-5 mmol/L | 7.78-8.5 mmol/L | 0.2 | 0.15 | Jee SH, 2005 |
| Liver Cancer | 4.2-5 mmol/L | 7.78-8.5 mmol/L | 0.38 | 0.04 | Kim K, 2018 |
| Liver Cancer | 4.8-5.56 mmol/L | 5.56-6.94 mmol/L | 0.03 | 0.03 | Tran TXM,2023 |
| Liver Cancer | 4.8-5.56 mmol/L | 5.56-6.94 mmol/L | 0.07 | 0.02 | Kim K, 2023 |
| Liver Cancer | 4.8-5.56 mmol/L | 7-7.83 mmol/L | 0.56 | 0.03 | Tran TXM, 2023 |
| Liver Cancer | 4.8-5.56 mmol/L | 7-7.83 mmol/L | 0.36 | 0.03 | Kim K, 2023 |
| Liver Cancer | 5.32-7 mmol/L | 7-7.83 mmol/L | 0.34 | 0.26 | Goto A, 2020 |
| Liver Cancer | 5.32-6.99 mmol/L | 7-7.83 mmol/L | 0.78 | 0.17 | Kuriki K, 2007 |
| Liver Cancer | 5.32-6.99 mmol/L | 7-7.83 mmol/L | 0.82 | 0.39 | Kuriki K, 2007 |
| Liver Cancer | 5.32-6.99 mmol/L | 7-7.83 mmol/L | 0.44 | 0.44 | Yoshihisa F, 2001 |
| Liver Cancer | 5.32-6.99 mmol/L | 7-7.83 mmol/L | 0.56 | 0.87 | Yoshihisa F, 2001 |
| Liver Cancer | 5.32-6.99 mmol/L | 7-7.83 mmol/L | 0.33 | 0.77 | Yoshihisa F, 2001 |
| Liver Cancer | 5.32-6.99 mmol/L | 7-7.83 mmol/L | 0.8 | 0.5 | Yoshihisa F, 2001 |
| Liver Cancer | 5.32-6.99 mmol/L | 7-7.83 mmol/L | 0.89 | 0.82 | Yoshihisa F, 2001 |
| Liver Cancer | 5.32-6.99 mmol/L | 7-7.83 mmol/L | 3.08 | 1.12 | Yuichi T, 2007 |
| Liver Cancer | 5.32-6.99 mmol/L | 7-7.83 mmol/L | 0.36 | 0.01 | Kim SK, 2020 |
| Liver Cancer | 5.32-6.99 mmol/L | 7-7.83 mmol/L | 0.4 | 0.01 | Kim SK, 2020 |
| Liver Cancer | 5.32-6.99 mmol/L | 7-7.83 mmol/L | 0.29 | 0.02 | Kim SK, 2020 |
| Liver Cancer | 5.56-6.61 mmol/L | 10-15 mmol/L | 0.17 | 0.05 | Yoo JJ, 2021 |
| Liver Cancer | 4.92-6.13 mmol/L | 6.14-7 mmol/L | -0.01 | 0.44 | Campbell PT, 2022 |
| Liver Cancer | 4.92-6.13 mmol/L | 7-8.93 mmol/L | 0.7 | 0.53 | Campbell PT, 2022 |
| Liver Cancer | 5.06-6.99 mmol/L | 7-8.93 mmol/L | 0.86 | 0.14 | Conway RBN, 2021 |
| Liver Cancer | 5.06-6.99 mmol/L | 7-8.93 mmol/L | 1.02 | 0.35 | Hu Y, 2021 |
| Liver Cancer | 5.06-6.99 mmol/L | 7-8.93 mmol/L | 1.34 | 0.26 | Hu Y, 2021 |
| Liver Cancer | 5.06-6.99 mmol/L | 7-8.93 mmol/L | 0.75 | 0.2 | Hatia RI, 2023 |
| Liver Cancer | 5.06-6.99 mmol/L | 7-8.93 mmol/L | 0.74 | 0.28 | Hatia RI, 2023 |
| Liver Cancer | 5.06-6.99 mmol/L | 7-8.93 mmol/L | 0.77 | 0.08 | El-serag HB, 2004 |
| Liver Cancer | 5.06-6.99 mmol/L | 7-8.93 mmol/L | 0.34 | 0.28 | Ioannou GN, 2007 |
| Liver Cancer | 5.06-6.99 mmol/L | 7-8.93 mmol/L | 0.78 | 0.11 | Coughlin SS, 2004 |
| Liver Cancer | 5.06-6.99 mmol/L | 7-8.93 mmol/L | 0.31 | 0.19 | Coughlin SS, 2004 |
| Liver Cancer | 5.06-6.99 mmol/L | 7-8.93 mmol/L | 0.67 | 0.14 | Atchison EA, 2011 |
| Liver Cancer | 5.06-6.99 mmol/L | 7-8.93 mmol/L | 0.33 | 0.28 | Ulcickas Yood M, 2009 |
| Liver Cancer | 4.59-7 mmol/L | 7-9.44 mmol/L | 0.94 | 0.94 | Yilmaz Kavcar SR, 2022 |
| Liver Cancer | 4.72-6.11 mmol/L | 6.11-6.94 mmol/L | 0.34 | 0.29 | Gupta SP, 2013 |
| Liver Cancer | 4.72-6.11 mmol/L | 7-8.83 mmol/L | 0.86 | 0.38 | Gupta SP, 2013 |
| Liver Cancer | 4.78-5.11 mmol/L | 5.28-5.44 mmol/L | 0.25 | 0.34 | Loftfield E, 2016 |
| Liver Cancer | 4.78-5.11 mmol/L | 5.61-5.78 mmol/L | 0.63 | 0.31 | Loftfield E, 2016 |
| Liver Cancer | 4.78-5.11 mmol/L | 6.11-6.89 mmol/L | 0.88 | 0.3 | Loftfield E, 2016 |
| Liver Cancer | 4.41-5.56 mmol/L | 5.56-6.94 mmol/L | 0.53 | 0.19 | Xia B, 2021 |
| Liver Cancer | 4.41-5.56 mmol/L | 5.56-6.94 mmol/L | 0.25 | 0.32 | Xia B, 2021 |
| Liver Cancer | 4.41-5.56 mmol/L | 7-8.52 mmol/L | 1.06 | 0.21 | Xia B, 2021 |
| Liver Cancer | 4.41-5.56 mmol/L | 7-8.52 mmol/L | 0.89 | 0.36 | Xia B, 2021 |
| Liver Cancer | 4.68-6.13 mmol/L | 6.14-7.71 mmol/L | 0.78 | 0.2 | Rothwell JA, 2022 |
| Liver Cancer | 4.68-6.13 mmol/L | 6.14-7.71 mmol/L | 0.62 | 0.21 | Rothwell JA, 2022 |
| Liver Cancer | 4.88-6.99 mmol/L | 7-8.52 mmol/L | 1.39 | 0.07 | Wideroff L, 1997 |
| Liver Cancer | 4.88-6.99 mmol/L | 7-8.52 mmol/L | 0.74 | 0.13 | Wideroff L, 1997 |
| Liver Cancer | 4.88-6.99 mmol/L | 7-8.52 mmol/L | 1.57 | 0.26 | Wideroff L, 1997 |
| Liver Cancer | 4.88-6.99 mmol/L | 7-8.52 mmol/L | 1.16 | 0.06 | Wideroff L, 1997 |
| Liver Cancer | 4.88-6.99 mmol/L | 7-8.52 mmol/L | 0.6 | 0.39 | Chodick G, 2010 |
| Liver Cancer | 4.88-6.99 mmol/L | 7-8.52 mmol/L | 0.88 | 0.45 | Chodick G, 2010 |
| Liver Cancer | 4.88-6.99 mmol/L | 7-8.52 mmol/L | 0.94 | 0.12 | Vicentini M, 2022 |
| Liver Cancer | 4.88-6.99 mmol/L | 7-8.52 mmol/L | 0.06 | 0.21 | Costanzo GGD, 2008 |
| Liver Cancer | 4.88-6.99 mmol/L | 7-8.52 mmol/L | -0.04 | 0.38 | Costanzo GGD, 2008 |
| Liver Cancer | 4.88-6.99 mmol/L | 7-8.52 mmol/L | 0.59 | 0.16 | Verlato G, 2003 |
| Liver Cancer | 4.88-6.99 mmol/L | 7-8.52 mmol/L | 0.68 | 0.21 | Verlato G, 2003 |
| Liver Cancer | 4.88-6.99 mmol/L | 7-8.52 mmol/L | 1.4 | 0.02 | Hemminki K, 2023 |
| Liver Cancer | 4.88-6.99 mmol/L | 7-8.52 mmol/L | 1.29 | 0.05 | Hemminki K, 2023 |
| Liver Cancer | 4.88-6.99 mmol/L | 7-8.52 mmol/L | 1.2 | 0.04 | Bjornsdottir HH, 2020 |
| Liver Cancer | 4.88-6.99 mmol/L | 7-8.52 mmol/L | -0.02 | 0.16 | Bjornsdottir HH, 2020 |
| Liver Cancer | 4.88-6.99 mmol/L | 7-8.52 mmol/L | 1.48 | 0.22 | Attner B, 2012 |
| Liver Cancer | 4.88-6.99 mmol/L | 7-8.52 mmol/L | 0.89 | 0.26 | Attner B, 2012 |
| Liver Cancer | 4.88-6.99 mmol/L | 7-8.52 mmol/L | 2.19 | 0.47 | Attner B, 2012 |
| Liver Cancer | 4.88-6.99 mmol/L | 7-8.52 mmol/L | 1.1 | 0.18 | Attner B, 2012 |
| Liver Cancer | 4.88-6.99 mmol/L | 7-8.52 mmol/L | 0.22 | 0.04 | Liu X, 2012 |
| Liver Cancer | 4.88-6.99 mmol/L | 7-8.52 mmol/L | 0.7 | 0.18 | Wotton CJ, 2011 |
| Liver Cancer | 4.88-6.99 mmol/L | 7-8.52 mmol/L | 0.9 | 0.3 | Wotton CJ, 2011 |
| Liver Cancer | 4.88-6.99 mmol/L | 7-8.52 mmol/L | 0.01 | 0.58 | Swerdlow AJ, 2005 |
| Liver Cancer | 4.88-6.99 mmol/L | 7-8.52 mmol/L | 0.9 | 0.42 | Swerdlow AJ, 2005 |
| Liver Cancer | 4.88-6.99 mmol/L | 7-8.52 mmol/L | 1.25 | 0.48 | Ogunleye AA, 2009 |
| Lung Cancer | 5.28-6.99 mmol/L | 7-7.77 mmol/L | 0.39 | 0.02 | Gurney J, 2022 |
| Lung Cancer | 5.28-6.99 mmol/L | 7-7.77 mmol/L | 0.36 | 0.04 | Park SH, 2023 |
| Lung Cancer | 5.28-6.99 mmol/L | 7-7.77 mmol/L | 0.1 | 0.08 | Park SH, 2023 |
| Lung Cancer | 5.28-6.99 mmol/L | 7-7.77 mmol/L | 0.3 | 0.2 | Park SH, 2023 |
| Lung Cancer | 5.28-6.99 mmol/L | 7-7.77 mmol/L | 0.02 | 0.12 | Park SH, 2023 |
| Lung Cancer | 5.28-6.99 mmol/L | 7-7.77 mmol/L | 0.29 | 0.02 | Park SH, 2023 |
| Lung Cancer | 4.6-5.5 mmol/L | 5.6-6.9 mmol/L | 0.06 | 0.04 | Pan XF, 2018 |
| Lung Cancer | 4.6-5.5 mmol/L | 7-8.18 mmol/L | 0.1 | 0.05 | Pan XF, 2018 |
| Lung Cancer | 5.08-6.99 mmol/L | 7-8.18 mmol/L | -0.12 | 0.15 | Yang Z, 2023 |
| Lung Cancer | 5.08-6.99 mmol/L | 7-8.18 mmol/L | 0.16 | 0.08 | Yang Z, 2023 |
| Lung Cancer | 5.08-6.99 mmol/L | 7-8.18 mmol/L | -0.16 | 0.07 | Yang Z, 2023 |
| Lung Cancer | 5.08-6.99 mmol/L | 7-8.18 mmol/L | -0.12 | 0.1 | Yang Z, 2023 |
| Lung Cancer | 5.08-6.99 mmol/L | 7-8.18 mmol/L | -0.15 | 0.4 | Yang Z, 2023 |
| Lung Cancer | 5.08-6.99 mmol/L | 7-8.18 mmol/L | 0.04 | 0.15 | Yang Z, 2023 |
| Lung Cancer | 5.08-6.99 mmol/L | 7-8.18 mmol/L | -0.19 | 0.12 | Yang Z, 2023 |
| Lung Cancer | 5.08-6.99 mmol/L | 7-8.18 mmol/L | -0.2 | 0.13 | Yang Z, 2023 |
| Lung Cancer | 5.08-6.99 mmol/L | 7-8.18 mmol/L | 0.43 | 0.1 | Lee MY, 2012 |
| Lung Cancer | 3.23-5.49 mmol/L | 5.6-5.71 mmol/L | 0.16 | 0.24 | Yoo TK, 2022 |
| Lung Cancer | 3.23-5.49 mmol/L | 5.83-5.91 mmol/L | 0.39 | 0.25 | Yoo TK, 2022 |
| Lung Cancer | 3.23-5.49 mmol/L | 5.92-6.03 mmol/L | 0.1 | 0.23 | Yoo TK, 2022 |
| Lung Cancer | 3.23-5.49 mmol/L | 6.14-6.89 mmol/L | 0.61 | 0.22 | Yoo TK, 2022 |
| Lung Cancer | 3.33-5.56 mmol/L | 2.5-3.33 mmol/L | -0.27 | 0.38 | Park HJ, 2019 |
| Lung Cancer | 3.33-5.56 mmol/L | 5.56-6.94 mmol/L | 0.01 | 0.04 | Park HJ, 2019 |
| Lung Cancer | 3.33-5.56 mmol/L | 2.5-3.33 mmol/L | 0.34 | 0.45 | Park HJ, 2019 |
| Lung Cancer | 3.33-5.56 mmol/L | 5.56-6.94 mmol/L | -0.01 | 0.05 | Park HJ, 2019 |
| Lung Cancer | 4.2-5 mmol/L | 5-6.06 mmol/L | 0.02 | 0.03 | Jee SH, 2005 |
| Lung Cancer | 4.2-5 mmol/L | 6.06-6.94 mmol/L | 0.09 | 0.06 | Jee SH, 2005 |
| Lung Cancer | 4.2-5 mmol/L | 7-7.72 mmol/L | 0.08 | 0.1 | Jee SH, 2005 |
| Lung Cancer | 4.2-5 mmol/L | 5-6.06 mmol/L | 0.1 | 0.07 | Jee SH, 2005 |
| Lung Cancer | 4.2-5 mmol/L | 6.06-6.94 mmol/L | -0.11 | 0.15 | Jee SH, 2005 |
| Lung Cancer | 4.2-5 mmol/L | 7-7.72 mmol/L | 0.47 | 0.21 | Jee SH, 2005 |
| Lung Cancer | 4.2-5 mmol/L | 5-6.06 mmol/L | -0.02 | 0.03 | Jee SH, 2005 |
| Lung Cancer | 4.2-5 mmol/L | 6.06-6.94 mmol/L | 0.09 | 0.05 | Jee SH, 2005 |
| Lung Cancer | 4.2-5 mmol/L | 7-7.72 mmol/L | 0.13 | 0.09 | Jee SH, 2005 |
| Lung Cancer | 4.2-5 mmol/L | 5-6.06 mmol/L | 0.13 | 0.07 | Jee SH, 2005 |
| Lung Cancer | 4.2-5 mmol/L | 6.06-6.94 mmol/L | -0.01 | 0.12 | Jee SH, 2005 |
| Lung Cancer | 4.2-5 mmol/L | 7-7.72 mmol/L | 0.26 | 0.2 | Jee SH, 2005 |
| Lung Cancer | 4.2-5 mmol/L | 7.78-8.5 mmol/L | 0 | 0.07 | Jee SH, 2005 |
| Lung Cancer | 4.2-5 mmol/L | 7.78-8.5 mmol/L | 0.41 | 0.16 | Jee SH, 2005 |
| Lung Cancer | 4.2-5 mmol/L | 7.78-8.5 mmol/L | 0.01 | 0.06 | Jee SH, 2005 |
| Lung Cancer | 4.2-5 mmol/L | 7.78-8.5 mmol/L | 0.12 | 0.15 | Jee SH, 2005 |
| Lung Cancer | 4.8-5.56 mmol/L | 5.56-6.94 mmol/L | 0.04 | 0.02 | Tran TXM, 2023 |
| Lung Cancer | 4.8-5.56 mmol/L | 7-7.83 mmol/L | 0.12 | 0.01 | Tran TXM, 2023 |
| Lung Cancer | 5.32-7 mmol/L | 7-7.83 mmol/L | 0.34 | 0.17 | Goto A, 2020 |
| Lung Cancer | 5.32-6.99 mmol/L | 7-7.83 mmol/L | 0.43 | 0.12 | Kuriki K, 2007 |
| Lung Cancer | 5.32-6.99 mmol/L | 7-7.83 mmol/L | 0.48 | 0.24 | Kuriki K, 2007 |
| Lung Cancer | 5.32-6.99 mmol/L | 7-7.83 mmol/L | 0.13 | 0.01 | Kim SK, 2020 |
| Lung Cancer | 5.32-6.99 mmol/L | 7-7.83 mmol/L | 0.16 | 0.01 | Kim SK, 2020 |
| Lung Cancer | 5.32-6.99 mmol/L | 7-7.83 mmol/L | 0.08 | 0.02 | Kim SK, 2020 |
| Lung Cancer | 3.33-5.56 mmol/L | 7-7.83 mmol/L | 0.05 | 0.06 | Park HJ, 2019 |
| Lung Cancer | 3.33-5.56 mmol/L | 7-7.83 mmol/L | 0.09 | 0.1 | Park HJ, 2019 |
| Lung Cancer | 3.98-4.72 mmol/L | 4.78-5.11 mmol/L | -0.3 | 0.22 | Gathirua-Mwangi WG, 2017 |
| Lung Cancer | 3.98-4.72 mmol/L | 5.17-5.56 mmol/L | -0.43 | 0.22 | Gathirua-Mwangi WG, 2017 |
| Lung Cancer | 3.98-4.72 mmol/L | 5.61-7.2 mmol/L | -0.17 | 0.21 | Gathirua-Mwangi WG, 2017 |
| Lung Cancer | 5.06-6.99 mmol/L | 7-8.93 mmol/L | 0.12 | 0.04 | Johnson JA, 2011 |
| Lung Cancer | 5.06-6.99 mmol/L | 7-8.93 mmol/L | 0.03 | 0.06 | Leiter A, 2021 |
| Lung Cancer | 5.06-6.99 mmol/L | 7-8.93 mmol/L | 0.35 | 0.11 | Hu Y, 2021 |
| Lung Cancer | 5.06-6.99 mmol/L | 7-8.93 mmol/L | 0.18 | 0.08 | Hu Y, 2021 |
| Lung Cancer | 5.06-6.99 mmol/L | 7-8.93 mmol/L | -0.24 | 0.01 | Atchison EA, 2011 |
| Lung Cancer | 5.06-6.99 mmol/L | 7-8.93 mmol/L | 0.03 | 0.03 | Campbell PT, 0212 |
| Lung Cancer | 5.06-6.99 mmol/L | 7-8.93 mmol/L | 0.02 | 0.05 | Campbell PT, 0212 |
| Lung Cancer | 5.06-6.99 mmol/L | 7-8.93 mmol/L | 0.08 | 0.39 | Steenland K, 1995 |
| Lung Cancer | 5.06-6.99 mmol/L | 7-8.93 mmol/L | 0.78 | 0.45 | Steenland K, 1995 |
| Lung Cancer | 5.37-6.16 mmol/L | 6.16-7.05 mmol/L | 0.36 | 0.05 | Peila R, 2020 |
| Lung Cancer | 5.37-6.16 mmol/L | 6.16-7.05 mmol/L | -0.2 | 0.19 | Peila R, 2020 |
| Lung Cancer | 5.37-6.16 mmol/L | 5.37-7.04 mmol/L | -0.19 | 0.11 | Peila R, 2020 |
| Lung Cancer | 5.37-6.16 mmol/L | 5.37-7.04 mmol/L | 0.44 | 0.18 | Peila R, 2020 |
| Lung Cancer | 4.88-6.99 mmol/L | 7-8.52 mmol/L | 0 | 0.05 | Wideroff L, 1997 |
| Lung Cancer | 4.88-6.99 mmol/L | 7-8.52 mmol/L | -0.11 | 0.08 | Wideroff L, 1997 |
| Lung Cancer | 4.88-6.99 mmol/L | 7-8.52 mmol/L | 0.26 | 0.12 | Wideroff L, 1997 |
| Lung Cancer | 4.88-6.99 mmol/L | 7-8.52 mmol/L | -0.11 | 0.03 | Wideroff L, 1997 |
| Lung Cancer | 4.88-6.99 mmol/L | 7-8.52 mmol/L | 0.22 | 0.07 | Vicentini M, 2022 |
| Lung Cancer | 4.88-6.99 mmol/L | 7-8.52 mmol/L | -0.09 | 0.1 | Verlato G, 2003 |
| Lung Cancer | 4.88-6.99 mmol/L | 7-8.52 mmol/L | 0.27 | 0.21 | Verlato G, 2003 |
| Lung Cancer | 4.88-6.99 mmol/L | 7-8.52 mmol/L | 0.01 | 0.02 | Bjornsdottir HH, 2020 |
| Lung Cancer | 4.88-6.99 mmol/L | 7-8.52 mmol/L | -0.02 | 0.04 | Bjornsdottir HH, 2020 |
| Lung Cancer | 4.88-6.99 mmol/L | 7-8.52 mmol/L | 0.04 | 0.12 | Attner B, 2012 |
| Lung Cancer | 4.88-6.99 mmol/L | 7-8.52 mmol/L | 0.1 | 0.03 | Liu X, 2012 |
| Lung Cancer | 4.88-6.99 mmol/L | 7-8.52 mmol/L | 0.15 | 0.02 | Pradhan R, 2022 |
| Lung Cancer | 4.88-6.94 mmol/L | 7-8.52 mmol/L | 0.74 | 0.13 | Mulla K, 2023 |
| Lung Cancer | 5.37-6.16 mmol/L | 7.05-8.52 mmol/L | 0.17 | 0.1 | Peila R, 2020 |
| Lung Cancer | 5.37-6.16 mmol/L | 7.05-8.52 mmol/L | -0.12 | 0.35 | Peila R, 2020 |
| Lung Cancer | 4.88-6.99 mmol/L | 7-8.52 mmol/L | 0 | 0.14 | Hall GC, 2005 |
| Lung Cancer | 4.88-6.99 mmol/L | 7-8.52 mmol/L | 0.28 | 0.28 | Hall GC, 2005 |
| Lung Cancer | 4.88-6.99 mmol/L | 7-8.52 mmol/L | 0.02 | 0.27 | Hall GC, 2005 |
| Lung Cancer | 4.88-6.99 mmol/L | 7-8.52 mmol/L | 0.13 | 0.17 | Hall GC, 2005 |
| Lung Cancer | 4.88-6.99 mmol/L | 7-8.52 mmol/L | 0.12 | 0.12 | Hall GC, 2005 |
| Lung Cancer | 4.88-6.99 mmol/L | 7-8.52 mmol/L | 0.22 | 0.43 | Hall GC, 2005 |
| Lung Cancer | 4.88-6.99 mmol/L | 7-8.52 mmol/L | -0.02 | 0.08 | Hall GC, 2005 |
| Lung Cancer | 4.88-6.99 mmol/L | 7-8.52 mmol/L | -0.17 | 0.12 | Swerdlow AJ, 2005 |
| Lung Cancer | 4.88-6.99 mmol/L | 7-8.52 mmol/L | -0.21 | 0.14 | Swerdlow AJ, 2005 |
| Lung Cancer | 4.88-6.99 mmol/L | 7-8.52 mmol/L | 0.04 | 0.07 | Wotton CJ, 2011 |
| Lung Cancer | 4.88-6.99 mmol/L | 7-8.52 mmol/L | 0.03 | 0.13 | Wotton CJ, 2011 |
| Lung Cancer | 4.88-6.99 mmol/L | 7-8.52 mmol/L | -0.36 | 0.18 | Ogunleye AA, 2009 |
| Ovarian Cancer | 5.28-6.99 mmol/L | 7-7.77 mmol/L | 0.16 | 0.07 | Gurney J, 2022 |
| Ovarian Cancer | 5.28-6.99 mmol/L | 7-7.77 mmol/L | 0.07 | 0.77 | Park SH, 2023 |
| Ovarian Cancer | 5.28-6.99 mmol/L | 7-7.77 mmol/L | 0.18 | 0.21 | Park SH, 2023 |
| Ovarian Cancer | 5.28-6.99 mmol/L | 7-7.77 mmol/L | 0.08 | 0.39 | Park SH, 2023 |
| Ovarian Cancer | 5.28-6.99 mmol/L | 7-7.77 mmol/L | 0.13 | 0.08 | Park SH, 2023 |
| Ovarian Cancer | 4.8-5.56 mmol/L | 5.56-6.94 mmol/L | 0.05 | 0.04 | Tran TXM, 2023 |
| Ovarian Cancer | 4.8-5.56 mmol/L | 5.56-6.94 mmol/L | -0.02 | 0.05 | Kim K, 2023 |
| Ovarian Cancer | 4.8-5.56 mmol/L | 7-7.83 mmol/L | 0.12 | 0.06 | Tran TXM, 2023 |
| Ovarian Cancer | 4.8-5.56 mmol/L | 7-7.83 mmol/L | -0.29 | 0.14 | Kim K, 2023 |
| Ovarian Cancer | 4.92-6.13 mmol/L | 6.14-7 mmol/L | 0.36 | 0.28 | Campbell PT, 2022 |
| Ovarian Cancer | 4.92-6.13 mmol/L | 7-8.93 mmol/L | 0.58 | 0.41 | Campbell PT, 2022 |
| Ovarian Cancer | 5.06-6.99 mmol/L | 7-8.93 mmol/L | -0.21 | 0.16 | Hu Y, 2021 |
| Ovarian Cancer | 4.59-7 mmol/L | 7-9.44 mmol/L | 0.22 | 1.14 | Yilmaz Kavcar SR, 2022 |
| Ovarian Cancer | 5.37-6.16 mmol/L | 6.16-7.05 mmol/L | -0.16 | 0.13 | Peila R, 2020 |
| Ovarian Cancer | 5.37-6.16 mmol/L | 5.37-7.04 mmol/L | -0.01 | 0.15 | Peila R, 2020 |
| Ovarian Cancer | 4.88-6.99 mmol/L | 7-8.52 mmol/L | 0.44 | 0.22 | Vicentini M, 2022 |
| Ovarian Cancer | 4.88-6.99 mmol/L | 7-8.52 mmol/L | -0.08 | 0.05 | Bjornsdottir HH, 2020 |
| Ovarian Cancer | 4.88-6.99 mmol/L | 7-8.52 mmol/L | 0.09 | 0.04 | Pradhan R, 2022 |
| Ovarian Cancer | 5.37-6.16 mmol/L | 7.05-8.52 mmol/L | 0.26 | 0.23 | Peila R, 2020 |
| Ovarian Cancer | 4.68-6.1 mmol/L | 6.1-6.9 mmol/L | 0.21 | 0.18 | Lambe M, 2011 |
| Ovarian Cancer | 4.68-6.1 mmol/L | 7-8.52 mmol/L | -0.02 | 0.22 | Lambe M, 2011 |
| Ovarian Cancer | 4.88-6.99 mmol/L | 7-8.52 mmol/L | -0.11 | 0.09 | Wideroff L, 1997. |
| Ovarian Cancer | 4.88-6.99 mmol/L | 7-8.52 mmol/L | 0.44 | 0.22 | Vicentini M, 2022. |
| Ovarian Cancer | 4.88-6.99 mmol/L | 7-8.52 mmol/L | -0.22 | 0.2 | Parazzini F, 1997. |
| Ovarian Cancer | 4.88-6.99 mmol/L | 7-8.52 mmol/L | -0.08 | 0.05 | Bjornsdottir HH, 2020 |
| Ovarian Cancer | 4.88-6.99 mmol/L | 7-8.52 mmol/L | -0.06 | 0.28 | Attner B, 2012. |
| Ovarian Cancer | 4.88-6.99 mmol/L | 7-8.52 mmol/L | 0.09 | 0.04 | Pradhan R, 2022 |
| Ovarian Cancer | 5.37-6.16 mmol/L | 7.05-8.52 mmol/L | 0.26 | 0.23 | Peila R, 2020 |
| Ovarian Cancer | 4.88-6.99 mmol/L | 7-8.52 mmol/L | 0.25 | 0.18 | Wotton CJ, 2011 |
| Ovarian Cancer | 4.88-6.99 mmol/L | 7-8.52 mmol/L | -0.07 | 0.39 | Wotton CJ, 2011. |
| Ovarian Cancer | 4.88-6.99 mmol/L | 7-8.52 mmol/L | -0.15 | 0.45 | Swerdlow AJ, 2005. |
| Ovarian Cancer | 4.88-6.99 mmol/L | 7-8.52 mmol/L | -0.33 | 0.46 | Swerdlow AJ, 2005 |
| Ovarian Cancer | 3.98-4.86 mmol/L | 4.86-5.25 mmol/L | -0.11 | 0.26 | Kabat GC, 2018 |
| Ovarian Cancer | 3.98-4.86 mmol/L | 5.25-5.8 mmol/L | 0.01 | 0.26 | Kabat GC, 2018 |
| Ovarian Cancer | 3.98-4.86 mmol/L | 5.81-7.2 mmol/L | 0.24 | 0.28 | Kabat GC, 2018 |
| Ovarian Cancer | 5.01-7.8 mmol/L | 7.81-9.17 mmol/L | 0.87 | 0.26 | Chodick G, 2010 |
| Pancreatic Cancer | 5.28-6.99 mmol/L | 7-7.77 mmol/L | 0.83 | 0.28 | Davis TME, 2022 |
| Pancreatic Cancer | 5.28-6.99 mmol/L | 7-7.77 mmol/L | 0.75 | 0.04 | Ali S, 2022 |
| Pancreatic Cancer | 5.28-6.99 mmol/L | 7-7.77 mmol/L | 0.85 | 0.03 | Gurney J, 2022 |
| Pancreatic Cancer | 5.28-6.99 mmol/L | 7-7.77 mmol/L | 0.68 | 0.1 | Park SH, 2023 |
| Pancreatic Cancer | 5.28-6.99 mmol/L | 7-7.77 mmol/L | 0.6 | 0.18 | Park SH, 2023 |
| Pancreatic Cancer | 5.28-6.99 mmol/L | 7-7.77 mmol/L | 1.26 | 0.35 | Park SH, 2023 |
| Pancreatic Cancer | 5.28-6.99 mmol/L | 7-7.77 mmol/L | 1.01 | 0.21 | Park SH, 2023 |
| Pancreatic Cancer | 5.28-6.99 mmol/L | 7-7.77 mmol/L | 0.89 | 0.04 | Park SH, 2023 |
| Pancreatic Cancer | 4.84-6.99 mmol/L | 7-8.83 mmol/L | 1.36 | 0.26 | Petrusel L, 2020 |
| Pancreatic Cancer | 4.78-6.99 mmol/L | 7-9.26 mmol/L | 0.55 | 0.01 | HernÃ¡ndez-GarduÃ±o E, 2021 |
| Pancreatic Cancer | 4.6-5.59 mmol/L | 5.6-7 mmol/L | 0.08 | 0.25 | Cai J, 2022 |
| Pancreatic Cancer | 4.6-5.59 mmol/L | 7-8.18 mmol/L | -0.16 | 0.45 | Cai J, 2022 |
| Pancreatic Cancer | 4.89-5.99 mmol/L | 6-7.9 mmol/L | -0.11 | 0.11 | Shen B, 2022 |
| Pancreatic Cancer | 4.89-5.99 mmol/L | 8-9.9 mmol/L | 0.25 | 0.13 | Shen B, 2022 |
| Pancreatic Cancer | 4.89-6.1 mmol/L | 6.1-7.31 mmol/L | 1.44 | 0.22 | Wu Q, 2012 |
| Pancreatic Cancer | 4.89-5.99 mmol/L | 10-15 mmol/L | 0.85 | 0.15 | Shen B, 2022 |
| Pancreatic Cancer | 5.08-6.99 mmol/L | 7-8.18 mmol/L | 1.66 | 0.47 | Zhao Q, 2023 |
| Pancreatic Cancer | 5.08-6.99 mmol/L | 7-8.18 mmol/L | 0.2 | 0.41 | Zhao Q, 2023 |
| Pancreatic Cancer | 5.08-6.99 mmol/L | 7-8.18 mmol/L | 2.2 | 0.59 | Yang Z, 2023 |
| Pancreatic Cancer | 5.08-6.99 mmol/L | 7-8.18 mmol/L | 0.87 | 0.24 | Yang Z, 2023 |
| Pancreatic Cancer | 5.08-6.99 mmol/L | 7-8.18 mmol/L | 0.51 | 0.17 | Yang Z, 2023 |
| Pancreatic Cancer | 5.08-6.99 mmol/L | 7-8.18 mmol/L | 0.1 | 0.23 | Yang Z, 2023 |
| Pancreatic Cancer | 5.08-6.99 mmol/L | 7-8.18 mmol/L | 0.56 | 0.33 | Yang Z, 2023 |
| Pancreatic Cancer | 5.08-6.99 mmol/L | 7-8.18 mmol/L | 0.5 | 0.2 | Yang Z, 2023 |
| Pancreatic Cancer | 5.08-6.99 mmol/L | 7-8.18 mmol/L | -0.05 | 0.26 | Yang Z, 2023 |
| Pancreatic Cancer | 5.08-6.99 mmol/L | 7-8.18 mmol/L | 0.42 | 0.06 | Lo S-F, 2013 |
| Pancreatic Cancer | 5.08-6.99 mmol/L | 7-8.18 mmol/L | 0.93 | 0.13 | Er KC, 2016 |
| Pancreatic Cancer | 4.2-5 mmol/L | 5-5.5 mmol/L | 0.09 | 0.17 | Kim NH, 2020 |
| Pancreatic Cancer | 4.2-5 mmol/L | 5.56-6.94 mmol/L | 0.16 | 0.19 | Kim NH, 2020 |
| Pancreatic Cancer | 4.2-4.89 mmol/L | 4.89-5.39 mmol/L | 0.39 | 0.17 | Kim YJ, 2022 |
| Pancreatic Cancer | 4.2-4.89 mmol/L | 5.39-6.06 mmol/L | 0.48 | 0.17 | Kim YJ, 2022 |
| Pancreatic Cancer | 4.2-5 mmol/L | 5-5.5 mmol/L | 0.08 | 0.05 | Koo DH, 2023 |
| Pancreatic Cancer | 4.2-5 mmol/L | 5.56-6.06 mmol/L | 0.18 | 0.05 | Koo DH, 2023 |
| Pancreatic Cancer | 4.2-5 mmol/L | 6.11-6.94 mmol/L | 0.25 | 0.06 | Koo DH, 2023 |
| Pancreatic Cancer | 4.2-5 mmol/L | 7-8.28 mmol/L | 0.31 | 0.08 | Koo DH, 2023 |
| Pancreatic Cancer | 4.2-5 mmol/L | 5-6.06 mmol/L | 0.08 | 0.07 | Jee SH, 2005 |
| Pancreatic Cancer | 4.2-5 mmol/L | 6.06-6.94 mmol/L | 0.25 | 0.11 | Jee SH, 2005 |
| Pancreatic Cancer | 4.2-5 mmol/L | 7-7.72 mmol/L | 0.37 | 0.2 | Jee SH, 2005 |
| Pancreatic Cancer | 4.2-5 mmol/L | 5-6.06 mmol/L | 0.37 | 0.11 | Jee SH, 2005 |
| Pancreatic Cancer | 4.2-5 mmol/L | 6.06-6.94 mmol/L | 0.53 | 0.19 | Jee SH, 2005 |
| Pancreatic Cancer | 4.2-5 mmol/L | 5-6.06 mmol/L | 0.08 | 0.07 | Jee SH, 2005 |
| Pancreatic Cancer | 4.2-5 mmol/L | 6.06-6.94 mmol/L | 0.29 | 0.1 | Jee SH, 2005 |
| Pancreatic Cancer | 4.2-5 mmol/L | 7-7.72 mmol/L | 0.31 | 0.19 | Jee SH, 2005 |
| Pancreatic Cancer | 4.2-5 mmol/L | 5-6.06 mmol/L | 0.24 | 0.11 | Jee SH, 2005 |
| Pancreatic Cancer | 4.2-5 mmol/L | 6.06-6.94 mmol/L | 0.33 | 0.19 | Jee SH, 2005 |
| Pancreatic Cancer | 4.2-5 mmol/L | 7-7.72 mmol/L | 0.69 | 0.29 | Jee SH, 2005 |
| Pancreatic Cancer | 4.2-4.89 mmol/L | 6.06-7.13 mmol/L | 0.84 | 0.16 | Kim YJ, 2022 |
| Pancreatic Cancer | 4.2-5 mmol/L | 7-7.83 mmol/L | 0.66 | 0.15 | Kim NH, 2020 |
| Pancreatic Cancer | 4.2-5 mmol/L | 7-7.83 mmol/L | 0.72 | 0.18 | Jee SH, 2005 |
| Pancreatic Cancer | 4.2-5 mmol/L | 8.33-8.5 mmol/L | 0.64 | 0.08 | Koo DH, 2023 |
| Pancreatic Cancer | 4.2-5 mmol/L | 7.78-8.5 mmol/L | 0.65 | 0.12 | Jee SH, 2005 |
| Pancreatic Cancer | 4.2-5 mmol/L | 7.78-8.5 mmol/L | 0.74 | 0.11 | Jee SH, 2005 |
| Pancreatic Cancer | 4.2-5 mmol/L | 7.78-8.5 mmol/L | 0.51 | 0.22 | Jee SH, 2005 |
| Pancreatic Cancer | 4.8-5.56 mmol/L | 5.56-6.94 mmol/L | -0.19 | 0.54 | Chung HH, 2022 |
| Pancreatic Cancer | 4.8-5.56 mmol/L | 5.56-6.94 mmol/L | 0.15 | 0.02 | Tran TXM, 2023 |
| Pancreatic Cancer | 4.8-5.56 mmol/L | 5.56-6.94 mmol/L | 0.18 | 0.06 | Yun JE, 2006 |
| Pancreatic Cancer | 4.8-5.56 mmol/L | 5.56-6.94 mmol/L | 0.18 | 0.06 | Yun JE, 2006 |
| Pancreatic Cancer | 4.8-5.56 mmol/L | 7-7.83 mmol/L | 0.24 | 0.34 | Chung HH, 2022 |
| Pancreatic Cancer | 4.8-5.56 mmol/L | 7-7.83 mmol/L | 0.48 | 0.03 | Tran TXM, 2023 |
| Pancreatic Cancer | 4.8-5.56 mmol/L | 7-7.83 mmol/L | 0.64 | 0.11 | Yun JE, 2006 |
| Pancreatic Cancer | 4.8-5.56 mmol/L | 7-7.83 mmol/L | 0.53 | 0.1 | Yun JE, 2006 |
| Pancreatic Cancer | 5.12-6.13 mmol/L | 6.14-6.35 mmol/L | 0.1 | 0.24 | Kim NH, 2020 |
| Pancreatic Cancer | 5.12-6.13 mmol/L | 6.46-7 mmol/L | 0.55 | 0.26 | Kim NH, 2020 |
| Pancreatic Cancer | 5.12-6.13 mmol/L | 7-7.83 mmol/L | 0.82 | 0.36 | Kim NH, 2020 |
| Pancreatic Cancer | 5.32-7 mmol/L | 7-7.83 mmol/L | 0.36 | 0.31 | Goto A, 2020 |
| Pancreatic Cancer | 5.32-6.99 mmol/L | 7-7.83 mmol/L | 0.82 | 0.27 | Kuriki K, 2007 |
| Pancreatic Cancer | 5.32-6.99 mmol/L | 7-7.83 mmol/L | 0.52 | 0.54 | Kuriki K, 2007 |
| Pancreatic Cancer | 5.32-6.99 mmol/L | 7-7.83 mmol/L | 0.68 | 0.01 | Kim SK, 2020 |
| Pancreatic Cancer | 5.32-6.99 mmol/L | 7-7.83 mmol/L | 0.72 | 0.02 | Kim SK, 2020 |
| Pancreatic Cancer | 5.32-6.99 mmol/L | 7-7.83 mmol/L | 0.63 | 0.02 | Kim SK, 2020 |
| Pancreatic Cancer | 4.92-6.13 mmol/L | 6.14-7 mmol/L | 0.4 | 0.19 | Campbell PT, 2022 |
| Pancreatic Cancer | 4.92-6.13 mmol/L | 7-8.93 mmol/L | 0.33 | 0.27 | Campbell PT, 2022 |
| Pancreatic Cancer | 5.06-6.99 mmol/L | 7-8.93 mmol/L | 0.38 | 0.13 | Farias AJ, 2020 |
| Pancreatic Cancer | 5.06-6.99 mmol/L | 7-8.93 mmol/L | 0.85 | 0.15 | Hu Y, 2021 |
| Pancreatic Cancer | 5.06-6.99 mmol/L | 7-8.93 mmol/L | 0.63 | 0.14 | Hu Y, 2021 |
| Pancreatic Cancer | 5.06-7 mmol/L | 7-8.93 mmol/L | 1.15 | 0.07 | Huang BZ, 2020 |
| Pancreatic Cancer | 5.06-7 mmol/L | 7-8.93 mmol/L | 0.62 | 0.05 | Huang BZ, 2020 |
| Pancreatic Cancer | 5.06-6.99 mmol/L | 7-8.93 mmol/L | 0.41 | 0.03 | Atchison EA, 2011 |
| Pancreatic Cancer | 5.06-6.99 mmol/L | 7-8.93 mmol/L | 0.39 | 0.08 | Coughlin SS, 2004 |
| Pancreatic Cancer | 5.06-6.99 mmol/L | 7-8.93 mmol/L | 0.36 | 0.09 | Coughlin SS, 2004 |
| Pancreatic Cancer | 5.06-6.99 mmol/L | 7-8.93 mmol/L | 1.2 | 0.23 | Ulcickas Yood M, 2009 |
| Pancreatic Cancer | 5.06-6.99 mmol/L | 7-8.93 mmol/L | 1.55 | 0.43 | Gupta S, 2006 |
| Pancreatic Cancer | 5.06-6.99 mmol/L | 7-8.93 mmol/L | 1.71 | 0.41 | Gupta S, 2006 |
| Pancreatic Cancer | 5.06-6.99 mmol/L | 7-8.93 mmol/L | 0.57 | 0.6 | Gupta S, 2006 |
| Pancreatic Cancer | 5.06-6.99 mmol/L | 7-8.93 mmol/L | 1.15 | 0.4 | Gupta S, 2006 |
| Pancreatic Cancer | 5.06-6.99 mmol/L | 7-8.93 mmol/L | 0.88 | 0.39 | Gupta S, 2006 |
| Pancreatic Cancer | 5.06-6.99 mmol/L | 7-8.93 mmol/L | 1.24 | 0.37 | Gupta S, 2006 |
| Pancreatic Cancer | 5.06-6.99 mmol/L | 7-8.93 mmol/L | 1.91 | 0.44 | Gupta S, 2006 |
| Pancreatic Cancer | 5.06-6.99 mmol/L | 7-8.93 mmol/L | 2.36 | 0.78 | Gupta S, 2006 |
| Pancreatic Cancer | 5.06-6.99 mmol/L | 7-8.93 mmol/L | 0.22 | 0.09 | El-Serag HB, 2009 |
| Pancreatic Cancer | 5.06-6.99 mmol/L | 7-8.93 mmol/L | 0.86 | 0.38 | Shibata Atsuko, 2006 |
| Pancreatic Cancer | 4.59-6.99 mmol/L | 7-9.44 mmol/L | 0.69 | 0.21 | MOMAYEZ SANA Z, 2021 |
| Pancreatic Cancer | 4.49-6.99 mmol/L | 7-8.9 mmol/L | 0.93 | 0.44 | Van Tran T, 2021 |
| Pancreatic Cancer | 4.41-5.17 mmol/L | 5.17-5.44 mmol/L | 0.14 | 0.29 | Stolzenberg-Solomon RZ, 2005 |
| Pancreatic Cancer | 4.41-5.17 mmol/L | 5.5-5.94 mmol/L | 0.4 | 0.28 | Stolzenberg-Solomon RZ, 2005 |
| Pancreatic Cancer | 4.41-5.17 mmol/L | 5.94-7.04 mmol/L | 0.52 | 0.28 | Stolzenberg-Solomon RZ, 2005 |
| Pancreatic Cancer | 4.41-5.56 mmol/L | 5.56-7.04 mmol/L | 0.47 | 0.1 | Xia B, 2020 |
| Pancreatic Cancer | 4.68-6.1 mmol/L | 6.1-7 mmol/L | 0.57 | 0.14 | Jacobson S, 2021 |
| Pancreatic Cancer | 4.68-6.1 mmol/L | 7-8.52 mmol/L | 0.65 | 0.49 | Jacobson S, 2021 |
| Pancreatic Cancer | 4.88-6.99 mmol/L | 7-8.52 mmol/L | 0.92 | 0.1 | Molina-Montes E, 2021 |
| Pancreatic Cancer | 4.88-6.99 mmol/L | 7-8.52 mmol/L | 0.66 | 0.21 | KirkegÃ¥rd J, 2020 |
| Pancreatic Cancer | 4.88-6.99 mmol/L | 7-8.52 mmol/L | -0.03 | 0.23 | KirkegÃ¥rd J, 2020 |
| Pancreatic Cancer | 4.88-6.99 mmol/L | 7-8.52 mmol/L | 0.53 | 0.07 | Wideroff L, 1997 |
| Pancreatic Cancer | 4.88-6.99 mmol/L | 7-8.52 mmol/L | 0.47 | 0.08 | Wideroff L, 1997 |
| Pancreatic Cancer | 4.88-6.99 mmol/L | 7-8.52 mmol/L | 0.34 | 0.3 | Wideroff L, 1997 |
| Pancreatic Cancer | 4.88-6.99 mmol/L | 7-8.52 mmol/L | 0.53 | 0.06 | Wideroff L, 1997 |
| Pancreatic Cancer | 4.88-6.99 mmol/L | 7-8.52 mmol/L | 0.54 | 0.05 | Li P, 2023 |
| Pancreatic Cancer | 4.88-6.99 mmol/L | 7-8.52 mmol/L | 0.71 | 0.14 | Li P, 2023 |
| Pancreatic Cancer | 4.88-6.99 mmol/L | 7-8.52 mmol/L | 1.19 | 0.08 | Li P, 2023 |
| Pancreatic Cancer | 4.88-6.99 mmol/L | 7-8.52 mmol/L | 2.07 | 0.12 | Li P, 2023 |
| Pancreatic Cancer | 4.88-6.99 mmol/L | 7-8.52 mmol/L | 0.39 | 0.25 | Chodick G, 2010 |
| Pancreatic Cancer | 4.88-6.99 mmol/L | 7-8.52 mmol/L | 0.64 | 0.25 | Chodick G, 2010 |
| Pancreatic Cancer | 4.88-6.99 mmol/L | 7-8.52 mmol/L | 0.55 | 0.1 | Vicentini M, 2022 |
| Pancreatic Cancer | 4.88-6.99 mmol/L | 7-8.52 mmol/L | -0.11 | 0.31 | Verlato G, 2003 |
| Pancreatic Cancer | 4.88-6.99 mmol/L | 7-8.52 mmol/L | 0.58 | 0.22 | Verlato G, 2003 |
| Pancreatic Cancer | 4.88-6.99 mmol/L | 7-8.52 mmol/L | 0.78 | 0.03 | Bjornsdottir HH, 2020 |
| Pancreatic Cancer | 4.88-6.99 mmol/L | 7-8.52 mmol/L | -0.02 | 0.03 | Bjornsdottir HH, 2020 |
| Pancreatic Cancer | 4.88-6.99 mmol/L | 7-8.52 mmol/L | 0.41 | 0.11 | Attner B, 2012 |
| Pancreatic Cancer | 4.88-6.99 mmol/L | 7-8.52 mmol/L | 0.98 | 0.25 | Attner B, 2012 |
| Pancreatic Cancer | 4.88-6.99 mmol/L | 7-8.52 mmol/L | 1.75 | 0.52 | Attner B, 2012 |
| Pancreatic Cancer | 4.88-6.99 mmol/L | 7-8.52 mmol/L | 0.67 | 0.2 | Attner B, 2012 |
| Pancreatic Cancer | 4.88-6.99 mmol/L | 7-8.52 mmol/L | 0.2 | 0.03 | Liu X, 2012 |
| Pancreatic Cancer | 4.88-6.99 mmol/L | 7-8.52 mmol/L | 0.84 | 0.34 | Larsson SC, 2005 |
| Pancreatic Cancer | 4.88-6.99 mmol/L | 7-8.52 mmol/L | 0.27 | 0.5 | Larsson SC, 2005 |
| Pancreatic Cancer | 4.88-6.99 mmol/L | 7-8.52 mmol/L | 1.13 | 0.17 | Dayem Ullah, AZM 2021 |
| Pancreatic Cancer | 4.88-6.99 mmol/L | 7-8.52 mmol/L | 0.67 | 0.23 | Dayem Ullah, AZM 2021 |
| Pancreatic Cancer | 4.88-6.99 mmol/L | 7-8.52 mmol/L | 0.55 | 0.14 | Dayem Ullah, AZM 2021 |
| Pancreatic Cancer | 4.88-6.99 mmol/L | 7-8.52 mmol/L | 1.04 | 0.03 | Pradhan R, 2022 |
| Pancreatic Cancer | 4.88-6.99 mmol/L | 7-8.52 mmol/L | 0.79 | 0.1 | Wotton CJ, 2011 |
| Pancreatic Cancer | 4.88-6.99 mmol/L | 7-8.52 mmol/L | 1.25 | 0.16 | Wotton CJ, 2011 |
| Pancreatic Cancer | 4.88-6.99 mmol/L | 7-8.52 mmol/L | 0.36 | 0.27 | Swerdlow AJ, 2005 |
| Pancreatic Cancer | 4.88-6.99 mmol/L | 7-8.52 mmol/L | 0.26 | 0.31 | Swerdlow AJ, 2005 |
| Pancreatic Cancer | 4.88-6.99 mmol/L | 7-8.52 mmol/L | 0.45 | 0.17 | Stevens Richard J, 2008 |
| Pancreatic Cancer | 4.88-6.99 mmol/L | 7-8.52 mmol/L | 1.05 | 0.41 | Ogunleye AA, 2009 |

## Section 6: Main risk curves of outcomes that rated < 2 stars.


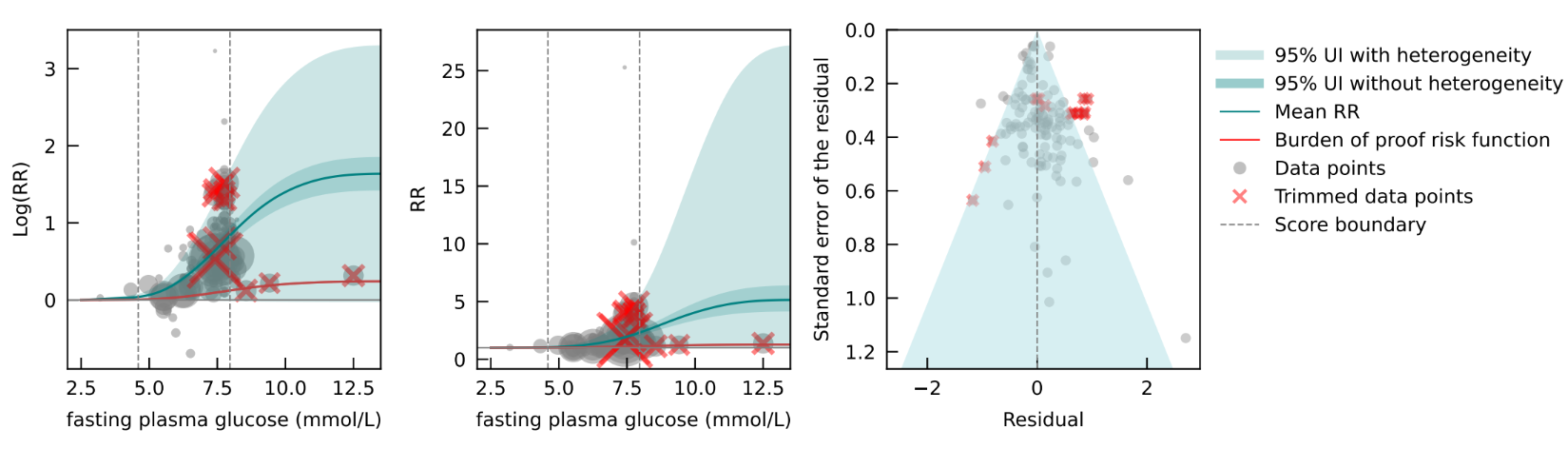


**Fig. S2: Fasting plasma glucose levels and liver cancer log relative risk (a), relative risk (b), and residuals by estimated standard deviation (c)**.


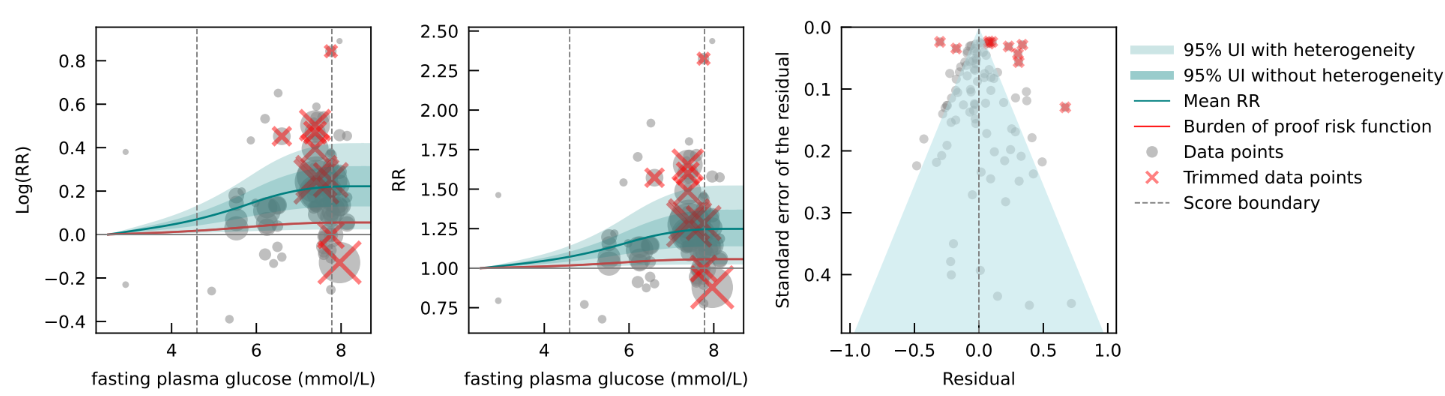


**Fig. S3: Fasting plasma glucose levels and lung cancer log relative risk (a), relative risk (b), and residuals by estimated standard deviation (c)**.


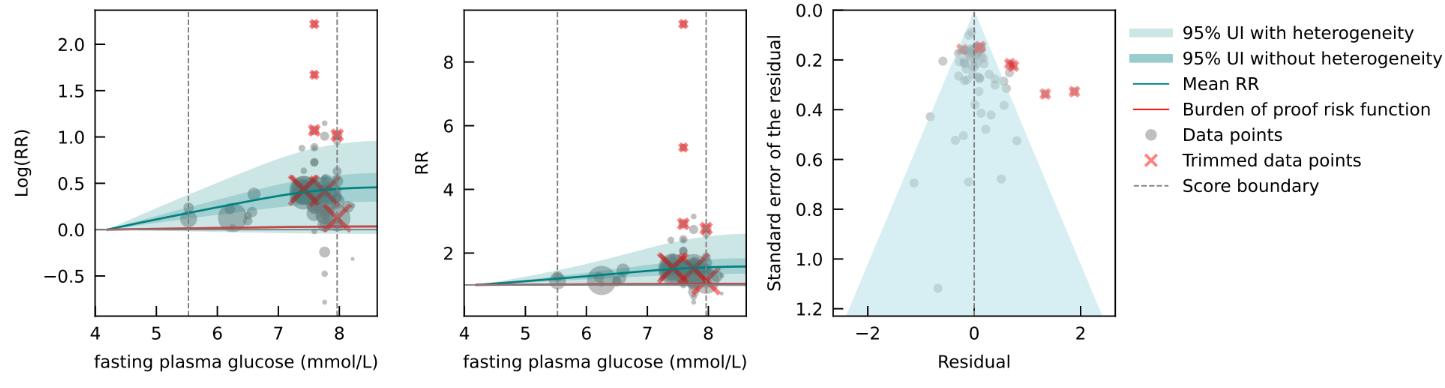


**Fig. S4: Fasting plasma glucose levels and bladder cancer log relative risk (a), relative risk (b), and residuals by estimated standard deviation (c)**.


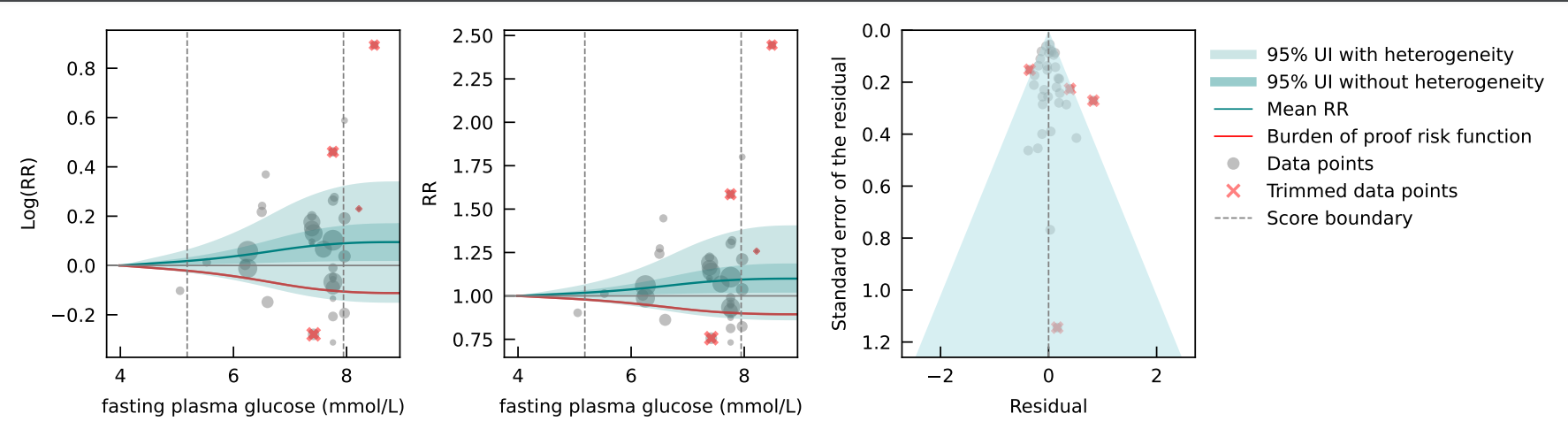


**Fig. S5: Fasting plasma glucose levels and ovarian cancer log relative risk (a), relative risk (b), and residuals by estimated standard deviation (c)**.

## Section 8: Sensitivity Analysis

### Table S9. Strength of the evidence for the relationship between exposure to high FPG levels and the seven health outcomes analyzed, when including only studies with cohort design (prospective or retrospective).

| Health outcome | 85^th^ percentile FPG level (mmol/L) | RR (95% UI) at  85th percentile | Exposure-averaged BPRF | Average  risk increase (%) | ROS | Star rating | Pub. bias | No. of studies | Selected bias covariates |
| --- | --- | --- | --- | --- | --- | --- | --- | --- | --- |
| Colon and rectum cancer | 7.96 | 1.58 (1.29, 1.93) | 1.20 | 20% | 0.2 | ⭐⭐⭐ | No | 45 | None |
| Pancreatic cancer | 7.96 | 2.23 (1.3, 3.83) | 1.16 | 16% | 0.14 | ⭐⭐⭐ | Yes | 39 | Diabetes definitions; Geographically representative sample |
| Breast cancer | 7.78 | 1.45 (1.14, 1.84) | 1.13 | 13% | 0.12 | ⭐⭐ | No | 36 | Geographically representative sample |
| Liver cancer | 7.96 | 2.31 (0.95, 5.62) | 1.04 | 4% | 0.04 | ⭐⭐ | No | 41 | Quality of adjustment, FPG levels imputation; Incident diabetes; Mortlity-speficit analysis; Geographically representative sample; Study outcome consistent with ICD-10 code for liver cancer |
| Ovarian cancer | 7.78 | 1.16 (1, 1.36) | 1.01 | 1% | 0.01 | ⭐⭐ | No | 9 | None |
| Tracheal, bronchus, and lung cancer | 7.94 | 1.31 (0.87, 1.97) | 0.95 | N/A | -0.06 | ⭐ | No | 28 | None |
| Bladder cancer | 7.96 | 1.44 (0.81, 2.56) | 0.92 | N/A | -0.08 | ⭐ | No | 26 | Study sample representative of general population; Diabetes definitions; Mortality-specific analysis; Geographically representative sample. |

The reported relative risk (RR) and its 95% uncertainty interval (UI) reflect the risk an individual who has been exposed to FPG has of developing the outcome of interest relative to that of someone who has not been exposed to FPG. Gamma (γ) is the estimated between-study heterogeneity. We report the 95% UI when not incorporating between-study heterogeneity (γ) − "95% UI without γ" − and when accounting for between-study heterogeneity − "95% UI with γ." The Burden of Proof Risk Function (BPRF) is calculated for risk-outcome pairs that were found to have significant relationships at an 0.05 level of significance when not incorporating between-study heterogeneity (i.e., the lower bound of the 95% UI without γ does not cross the null RR value of one). The BPRF corresponds to the 5th quantile estimate of relative risk, when additionally accounting for between-study heterogeneity closest to the null for each risk–outcome pair, and it reflects the most conservative estimate of excess risk associated with FPG that is consistent with the available data. Negative ROSs indicate that the evidence of the association is very weak and inconsistent. For ease of interpretation, we have transformed the ROS and BPRF into a star rating (0-5) with a higher rating representing a larger effect with stronger evidence. The potential existence of publication bias, which, if present, would affect the validity of the results, was tested using Egger's Regression. Included studies represent all available relevant data identified through our systematic reviews from January 1970 through May 2023. The selected bias covariates were chosen for inclusion in the model using an algorithm that systematically detects bias covariates that correspond to significant sources of bias in the observations included. If selected, the observations were adjusted to better reflect the gold standard values of the covariate. See the Supplementary Information for more information about the definition and how each bias covariates that were extracted for in each model.

### Table S10. Relative risks across exposure range

Below are the relative risks and 95% uncertainty intervals (RR and 95%UI) at clinically meaningful FPG levels when accounting for between-study heterogeneity. A FPG level of 6.1mmol/L is the cutoff for Prediabetes according to the American Diabetes Association, and a FPG of 7 mmol/L is the cutoff for defining *Diabetes Mellitus*.

| Outcome | RR (95%UI) at FPG = 6.1mmol/L  Prediabetes | RR (95%UI) at FPG = 7.0 mmol/L  Diabetes Mellitus |
| --- | --- | --- |
| Colorectal Cancer | 1.32 (1.17, 1.48) | 1.45 (1.23, 1.71) |
| Pancreatic Cancer | 1.30 (1.08, 1.55) | 1.73 (1.18, 2.54) |
| Breast Cancer | 1.34 (1.12, 1.60) | 1.44 (1.15, 1.80) |
| Lung Cancer | 1.17 (1.02, 1.35) | 1.23 (1.02, 1.47) |
| Bladder Cancer | 1.29 (0.97, 1.70) | 1.44 (0.96, 2.15) |
| Liver Cancer | 1.28 (1.00, 1.63) | 1.65 (0.99, 2.75) |
| Ovarian Cancer | 1.04 (0.94, 1.15) | 1.07 (0.90, 1.28) |
